# Evolutionary origins, molecular cloning and expression of carotenoid hydroxylases in eukaryotic photosynthetic algae

**DOI:** 10.1186/1471-2164-14-457

**Published:** 2013-07-08

**Authors:** Hongli Cui, Xiaona Yu, Yan Wang, Yulin Cui, Xueqin Li, Zhaopu Liu, Song Qin

**Affiliations:** 1 Key Laboratory of Coastal Biology and Biological Resources Utilization, Yantai Institute of Coastal Zone Research, Chinese Academy of Sciences, Yantai 264003, People’s Republic of China; 2 University of the Chinese Academy of Sciences, Beijing 100049, People’s Republic of China; 3 College of Resources and Environmental Sciences, Key Laboratory of Marine Biology, Nanjing Agricultural University, Nanjing 210095, People’s Republic of China; 4 Shenzhen Key Laboratory for Marine Bio-resource and Eco-environment, College of Life Sciences, Shenzhen University, Shenzhen 518060, People’s Republic of China

**Keywords:** Carotenoid hydroxylase, Xanthophylls biosynthesis, Structure and evolution, Molecular cloning, Expression profiles, Algae

## Abstract

**Background:**

Xanthophylls, oxygenated derivatives of carotenes, play critical roles in photosynthetic apparatus of cyanobacteria, algae, and higher plants. Although the xanthophylls biosynthetic pathway of algae is largely unknown, it is of particular interest because they have a very complicated evolutionary history. Carotenoid hydroxylase (CHY) is an important protein that plays essential roles in xanthophylls biosynthesis. With the availability of 18 sequenced algal genomes, we performed a comprehensive comparative analysis of *chy* genes and explored their distribution, structure, evolution, origins, and expression.

**Results:**

Overall 60 putative *chy* genes were identified and classified into two major subfamilies (*bch* and *cyp97*) according to their domain structures. Genes in the *bch* subfamily were found in 10 green algae and 1 red alga, but absent in other algae. In the phylogenetic tree, *bch* genes of green algae and higher plants share a common ancestor and are of non-cyanobacterial origin, whereas that of red algae is of cyanobacteria. The homologs of *cyp97a*/*c* genes were widespread only in green algae, while *cyp97b* paralogs were seen in most of algae. Phylogenetic analysis on *cyp97* genes supported the hypothesis that *cyp97b* is an ancient gene originated before the formation of extant algal groups. The *cyp97a* gene is more closely related to *cyp97c* in evolution than to *cyp97b*. The two *cyp97* genes were isolated from the green alga *Haematococcus pluvialis*, and transcriptional expression profiles of *chy* genes were observed under high light stress of different wavelength.

**Conclusions:**

Green algae received a *β*-xanthophylls biosynthetic pathway from host organisms. Although red algae inherited the pathway from cyanobacteria during primary endosymbiosis, it remains unclear in Chromalveolates. The *α*-xanthophylls biosynthetic pathway is a common feature in green algae and higher plants. The origination of *cyp97a*/*c* is most likely due to gene duplication before divergence of green algae and higher plants. Protein domain structures and expression analyses in green alga *H. pluvialis* indicate that various *chy* genes are in different manners response to light. The knowledge of evolution of *chy* genes in photosynthetic eukaryotes provided information of gene cloning and functional investigation of *chy* genes in algae in the future.

## Background

Carotenoids are isoprenoids which derived from the precursor molecule isopentenyl pyrophosphate and its isomer dimethylallyl diphosphate. All photosynthetic organisms including cyanobacteria, algae, and higher plants synthesize carotenoids [1,2]. Carotenoids are divided into two major groups: carotenes, which are enriched in the centers of photosystem reaction [3,4], and xanthophylls (including lutein, zeaxanthin, antheraxanthin, violaxanthin, and neoxanthin), oxygenated carotenoids that serve various functions in photosynthetic organisms and are essential for survival of the organism [5-8]. Of xanthophylls, lutein is the most abundant xanthophyll in all plant photosynthetic tissues, where it plays an important role in assembly and function of light harvesting complexes II (LHC II) [6-8]. Zeaxanthin is a structural isomer of lutein and a mainly component of the non-photochemical quenching (NPQ) mechanism involved in protecting the organism from photo-damage [9-11].

Untangling the phylogenomics of xanthophylls biosynthesis in eukaryotic algae requires basic knowledge of their evolutionary histories. Three algal phyla, the Rhodophyta (red algae), Glaucophyta, and Viridiplantae (land plants and green algae) acquired their plastids during primary endosymbiosis starting a heterotrophic eukaryote as a host and a phototrophic cyanobacterium as an endosymbiont [12-18]. Viridiplantae is comprised of two major evolutionary lineages that split in early: the Chlorophyta (Chlorophyceae, Ulvophyceae, Trebouxiophyceae, and Prasinophyceae) and the Streptophyta (Charophyte and Embryophytes) [19-21]. Subsequently, secondary endosymbiosis from green or red algae occurred, producing the extant diverse algal groups (for reviews, see [22,23]). Algae comprise a paraphyletic and polyphyletic group [24], as they do not include all the descendants of the last universal ancestor nor do they all descend from a common algal ancestor, although their plastids seem to have a single origin [25]. From an evolutionary perspective, carotenoid biosynthesis in photosynthetic organisms has become increasingly more complex and diverse [26]. Some photosynthetic bacteria, e.g., cyanobacteria contain carotenoids with *β*-rings (e.g. *β*-carotene), and many of them produce various mono- or di-hydroxy xanthophyll derivatives (e.g. zeaxanthin) [27,28]. In addition, pathway bifurcation occurred in some red algae and cyanobacteria (i.e. *Acaryochloris* and *Prochlorococcus*), and all green algae, and higher plants at the level of lycopene cyclization, yielding *α*-carotene from which *α*-carotene-derived xanthophylls (e.g. lutein) are synthesized [29-32]. As a consequence, the origins and evolution of xanthophylls biosynthetic pathway in algae lineages is of particular interesting.

Carotenoid hydroxylases (CHYs) comprise an important hydroxylase protein family performing the key enzymatic steps, hydroxylation reactions for *α*- and *β*-branch xanthophylls biosynthesis in photosynthetic organisms [33]. In higher plants, production of *α*-xanthophylls from *α*-carotene requires one *β*- and one *ϵ*-ring hydroxylation, while synthesis of *β*-xanthophylls from *β*-carotene requires two *β*-ring hydroxylations. Two classes of structurally unrelated enzymes catalyze these ring hydroxylations: two heme-containing cytochrome P450 hydroxylases (CYP97A3 and CYP97C1) and a pair of non-heme/di-iron hydroxylases (BCH1 and BCH2) [34-37]. Two *β*-ring hydroxylations in *β*-carotene are mediated by either P450-type CYP97A or BCH enzyme, during which zeaxanthin is produced [34-36]. Hydroxylations of *β*-ring and *ϵ*-ring in *α*-carotene are performed by CYP97A and CYP97C, respectively, producing lutein [37]. It is unknown why two different *β*-ring hydroxylases could have been maintained throughout the evolution. Probably, their own respective activities are not entirely interchangeable [38]. Different *chy* genes in higher plants are up-regulated often differentially, depending on environmental conditions or developmental stage of various tissues [6,8,26,33,36-40]. For algae, however, the research on xanthophylls biosynthesis is still in its infancy. For unicellular green algae, *bch* genes were investigated such as in *Haematococcus pluvialis* and *Chlamydomonas reinhardtii*[41-43]. Although lutein and its derivatives are detected in Rhodophyta (macrophytic type), Cryptophyta, Euglenophyta, Chlorarachniophyta, and Chlorophyta, enzymes involving in hydroxylation of *α*-carotene remains unknown [44]. Recently, a new P450 protein responsible for the hydroxylation of *ϵ*–ring of *α*-carotene in *Arabidopsis thaliana* was reported [37,45]. In addition, two genes encoding cytochrome P450-type carotenoid hydroxylases (*cyp97a4* and *cyp97c2*) were isolated from *Oryza sativa* and their functions were investigated in a *β*-carotene producing *Escherichia coli* strain [35]. Moreover, 25 cytochrome P450 oxidoreductases have been discovered so far in the green alga *Ulva linza*[46]. Therefore, it is evident that P450-type monooxygenases, in addition to non-heme hydroxylases such as BCH, are involved in carotenoid hydroxylation, which identified one of the missing pieces of carotenoid biosynthetic enzyme and provided valuable clues to study *chy* genes in algae in the future.

Recently, the genome sequences of a number of microalgae became available from the DOE Joint Genome Institute (http://genome.jgi.doe.gov/). Proteins coding sequences of each filtered model of these algae genomes with representatives from very different groups such as green algae, red algae, diatoms, and Haptophytes was performed to identify their *chy* genes. Details of the completeness of genome sequences used in this study were taken from DOE Joint Genome Institute project list (project list: http://genome.jgi.doe.gov/genome-projects/) [see Additional file 1: Table S1]. A BLASTp-HMMER-plus-phylogeny reconstruction approach was employed to analyze CHYs in focus of their distribution, structure, evolution, origins, and expression. In order to study the evolutionary histories of xanthophylls biosynthetic pathway from cyanobacteria to modern algae, candidate genes were then compared with known sequences of other organisms. Based on the predicted putative CHY-encoding genes, two full-length sequences of CYP97-encoding cDNA (*Haecyp97a* and *Haecyp97b*) were isolated from high-astaxanthin-production green alga *H. pluvialis* strain Flotow 1844 [42]. Expression profiles of different *chy* genes under high light stress of different wavelength were observed by means of relative quantitative real-time RT-PCR (Reverse Transcription-Polymerase Chain Reaction). Light is considered an effective stimulus inducing the expression of carotenoid biosynthesis-related genes and the astaxanthin accumulation in economic green alga *H. pluvialis*[47,48]. These studies provide significant insight into the origins and evolution of *chy* genes in photosynthetic eukaryotes and provide information for further gene cloning, functional characterization, and expression analysis of *chy* genes in algae. A better understanding of algal CHYs can help us to comprehend the roles of CHYs in xanthophylls biosynthetic under different adverse conditions.

## Results and discussion

### Identification, classification, and distribution of CHY proteins

**Figure 1:**
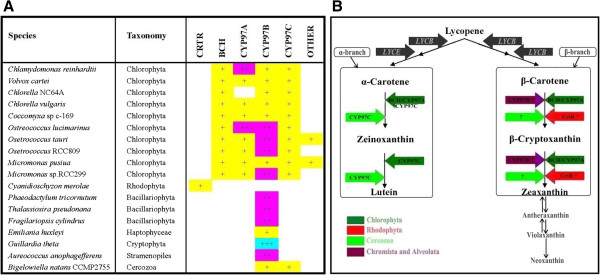
**Distribution of putative genes encoding CHYs and hypothesized xanthophylls biosynthetic pathway in algae. A]** The distribution of genes encoding BCH, CrtR and CYP97 homologs across 18 algal genome sequences. The number of putative homologs or paralogs in each corresponding genome is indicated by "+" with color codes, respectively. **B]** Hypothesized xanthophylls biosynthetic pathway in algae. The genes encoding putative BCH-, CrtR- or CYP97-homologs identified from different algae genomes were indicated with color arrows. Lutein and *α*-carotene are absent in red algae and Chromalveolates according to our results. Question mark indicates enzymes involved in xanthophylls biosynthesis are unclear.

18 algal nuclear genomes were examined for putative genes of CHY proteins. A summary of algal genes putatively encoding CHYs is shown in Table 1, and the classification and distribution of candidate genes, and hypothesized xanthophylls biosynthetic pathways across organisms are given in Figure 1. A total of 11 and 49 putative genes encoding BCH and CYP97, respectively, were predicted and annotated from 18 complete or incomplete eukaryotic algal genomes. Deduced protein sequences of genes encoding CHYs from the 18 algal genomes are shown in the Additional file [see Additional file 2]. Among the 18 eukaryotic photosynthetic algae, red algae possessed a *bch* homolog (CrtR-type) only. Hydroxylation of *β*-carotene in different organisms is primarily carried out by three gene subfamilies of the non-heme/di-iron monooxygenase superfamily: *bch* genes of higher plants and green algae; *bch* genes of non-photosynthetic bacteria; and *CrtR* genes of cyanobacteria [37]. Green algae inhabiting freshwater (Chlorophyceae: *C*. *reinhardtii* and *V. carteri*; Trebouxiophyceae: *C*. sp. NC64A, *C*. *vulgaris*, and *C*. sp. C-169) and marine environments (Prasinophyceae: *M. pusilla*, *M*. sp. RCC299, *O*. sp. RCC809, *O. tauri*, and *O. lucimarinus*) each possessed a *bch* homolog. No *CrtR*- or *bch*-homolog was discovered in those algal strains from red algal secondary endosymbiotic event. Previous studies, however, demonstrated a xanthophyll cycle (zeaxanthin-antheraxanthin-violaxanthin), with violaxanthin the putative precursor of both diadinoxanthin and fucoxanthin in the diatom *Phaeodactylum tricornutum*[49,50], implying that this enzymatic reaction may therefore be catalyzed by other unrelated enzymes, such as LUT-like P450 proteins in those algal strains from Chromalveolates [51]. Therefore, isolation and characterization of novel enzymes involved in *β*-xanthophylls (zeaxanthin) biosynthesis in Chromalveolates are of going research.

**Table 1:** Putative carotenoid hydroxylase genes identified in 18 algae genomes

**Gene locations (Locations are indicated by positions on either chromosomes or scaffolds)**	**Protein length**	**Types**
**Chlorophyta----*Chlamydomonas reinhardtii***		
jgi|Chlre4|309780|kg.chromosome_8_#_293_#_ABQ59243.1	577	CYP97C
jgi|Chlre4|196742|DNE_DNE_gwH.55.10.1	652	CYP97A
jgi|Chlre4|196744|DNE_DNE_e_gwW.42.59.1	655	CYP97A
jgi|Chlre4|283001|au.g1522_t1	502	CYP97B
jgi|Chlre4|164400|fgenesh2_kg.C_scaffold_40000013	298	BCH
**Chlorophyta----*Volvox carteri***		
jgi|Volca1|83281|estExt_Genewise1Plus. C_520019	576	CYP97C
jgi|Volca1|100143|fgenesh4_pg.C_scaffold_106000021	672	CYP97A
jgi|Volca1|65884|e_gw1.51.4.1	642	CYP97B
jgi|Volca1|44641|gw1.75.40.1	294	BCH
**Chlorophyta---- *Chlorella *NC64A**		
jgi|ChlNC64A_1|51247|fgenesh3_pg. C_scaffold_6000061	578	CYP97C
jgi|ChlNC64A_1|138471|IGS.gm_20_00220	615	CYP97B
jgi|ChlNC64A_1|24463|e_gw1.13.95.1	238	BCH
**Chlorophyta----*Chlorella vulgaris***		
jgi|Chlvu1|60845|GG.C169_S07_00141	541	CYP97C
jgi|Chlvu1|24726|e_gw1.1.89.1	535	CYP97A
jgi|Chlvu1|26454|e_gw1.3.111.1	534	CYP97B
jgi|Chlvu1|44442|estExt_Genewise1Plus.C_160017	347	BCH
**Chlorophyta---- *Coccomyxa *sp c-169**		
jgi|Coc_C169_1|63212|Genemark1.3995_g	541	CYP97C
jgi|Coc_C169_1|52277|estExt_fgenesh1_pm.C_10317	432	CYP97A
jgi|Coc_C169_1|12656|e_gw1.3.115.1	534	CYP97B
jgi|Coc_C169_1|30875|estExt_Genewise1Plus.C_160317	347	BCH
**Chlorophyta----*Ostreococcus lucimarinus***		
jgi|Ost9901_3|33533|eugene.0900010237	545	CYP97C
jgi|Ost9901_3|47300|estExt_GenewiseEukaryote.C_Chr_130084	495	CYP97A
jgi|Ost9901_3|1824|gwEuk.21.62.1	461	CYP97A
jgi|Ost9901_3|29177|eugene.0100010571	564	CYP97B
jgi|Ost9901_3|18007|fgenesh1_pg.C_Chr_14000053	561	CYP97B
jgi|Ost9901_3|9013|gwEuk.10.453.1	153	BCH
**Chlorophyta----*Osetrococcus tauri***		
jgi|Ostta4|1830|gw1.09.00.100.1	490	CYP97C
jgi|Ostta4|23029|estExt_fgenesh1_pm.C_Chr_13.00010043	484	CYP97A
jgi|Ostta4|27418|estExt_gwp_GeneWisePlus.C_Chr_01.00010469	577	CYP97B
jgi|Ostta4|23060|estExt_fgenesh1_pm.C_Chr_15.00010014	485	CYP97B
jgi|Ostta4|18835|e_gw1.08.00.85.1	407	OTHER
jgi|Ostta4|5089|gw1.10.00.289.1	216	BCH
**Chlorophyta---- *Osetrococcus *RCC809**		
jgi|OstRCC809_1|1644|gw1.3.120.1	490	CYP97C
jgi|OstRCC809_1|38666|fgenesh1_pg.C_scaffold_13000165	542	CYP97A
jgi|OstRCC809_1|53931|estExt_Genewise1.C_21287	525	CYP97B
jgi|OstRCC809_1|87721|eugene1.0000120100	576	CYP97B
jgi|OstRCC809_1|16226|gw1.3.1299.1	153	BCH
**Chlorophyta----*Micromonas pusilla***		
jgi|MicpuC2|32152	550	CYP97C
jgi|MicpuC2|26780	580	CYP97A
jgi|MicpuC2|22138	530	CYP97B
jgi|MicpuC2|57732	526	OTHER
jgi|MicpuC2|11104	230	BCH
**Chlorophyta---- *Micromonas *sp. RCC299**		
jgi|MicpuN2|95887|estExt_Genewise2Plus.C_Chr_140254	542	CYP97C
jgi|MicpuN2|83128|e_gw2.06.152.1	525	CYP97A
jgi|MicpuN2|96121|estExt_Genewise2Plus.C_Chr_160324	539	CYP97B
jgi|MicpuN2|88940|e_gw2.16.55.1	574	CYP97B
jgi|MicpuN2|76186|gw2.07.573.1	232	BCH
**Rhodophyta----*Cyanidioschyzon merolae***		
gnl|CMER|CMV041C [pt] beta-carotene hydroxylase	259	CRTR
**Bacillariophyta----*Thalassiosira pseudonana***		
jgi|Thaps3|36235|e_gw1.9.19.1	667	CYP97B
jgi|Thaps3|264039|thaps1_ua_kg.chr_13000087	547	CYP97B
**Bacillariophyta----*Phaeodactylum tricornutum***		
jgi|Phatr2|26422|estExt_Genewise1.C_chr_50056	770	CYP97B
jgi|Phatr2|16586|e_gw1.27.30.1	539	CYP97B
**Bacillariophyta----*Fragilariopsis cylindrus***		
jgi|Fracy1|169705|estExt_Genewise1.C_61231	528	CYP97B
jgi|Fracy1|170430|estExt_Genewise1.C_71165	614	CYP97B
**Haptophyceae----*Emiliania huxleyi***		
jgi|Emihu1|463287|estExtDG_fgeneshEH_pg.C_230139	618	CYP97B
**Cryptophyta----*Guillardia theta***		
jgi|Guith1|114743|au.78_g15845	582	CYP97B
jgi|Guith1|88554|estExt_Genewise1Plus.C_630014	492	CYP97B
jgi|Guith1|158065|fgenesh2_pm.48_#_5	499	CYP97B
**Cercozoa---- *Bigelowiella natans *CCMP2755**		
jgi|Bigna1|39488|e_gw1.33.13.1	474	CYP97C
jgi|Bigna1|52980|estExt_Genewise1Plus.C_140020	545	CYP97B
**Stramenopiles----*Aureococcus anophagefferens***		
jgi|Auran1|19592	528	CYP97B
jgi|Auran1|34662	432	CYP97B

OTHER indicates two genes belonged to no one subfamily of CYP97.

Homologs of *cyp97a* and *cyp97c* genes are found in green algae, but absent in other algae, including red algae (*C. merolae*), Heterokontophyta (*P. tricornutum, F. cylindrus* and *T. pseudonana*), Haptophyta (*E. huxleyi*), Cryptophyta (*G. theta*), and Stramenopiles (*A. anophagefferens*). Interestingly, two *cyp97a* genes were predicted in *C. reinhardtii* and *O. lucimarinus*, indicating that lineage-specific gene duplications occurred during the evolution of these algae. Due to gene loss or the incompletely sequenced genome, only genes encoding CYP97B and CYP97C homologs and none gene encoding CYP97A homolog were predicted in *B. natans* CCMP2755. Moreover, none gene encoding CYP97A homolog has been discovered in *Chlorella* sp. NC64A. In this study, at least one copy of the gene encoding CYP97B was found to be widely distributed in most of algae, except for red algae, indicating that the originations of this gene is not due to secondary endosymbiosis.

### Domain structures of BCH-type CHYs in algae

*β*-Xanthophylls (zeaxanthin) are widely distributed in nature, and they are common in all photosynthetic eukaryotes, and many photosynthetic, or non-photosynthetic prokaryotes [26,52]. Non-heme/di-iron *β*-ring carotenoid hydroxylase (BCH) has long been regarded as the only enzyme involved in zeaxanthin synthesis by hydroxylating *β*–carotene. Genes encoding *β*-carotene hydroxylases have been identified and characterized from bacteria [53,54], cyanobacteria [55], algae [41,42] and higher plants [34]. The *bch* genes of algae and higher plants encode the members of the fatty acid hydroxylase superfamily [PF04116] that also includes fatty acid and carotene hydroxylases and sterol desaturases. Members of this superfamily are integral membrane proteins and contain two copies of histidine-rich iron-binding motifs (HXXHH), needed for binding this cofactor with enzymes [34,56].

**Figure 2:**
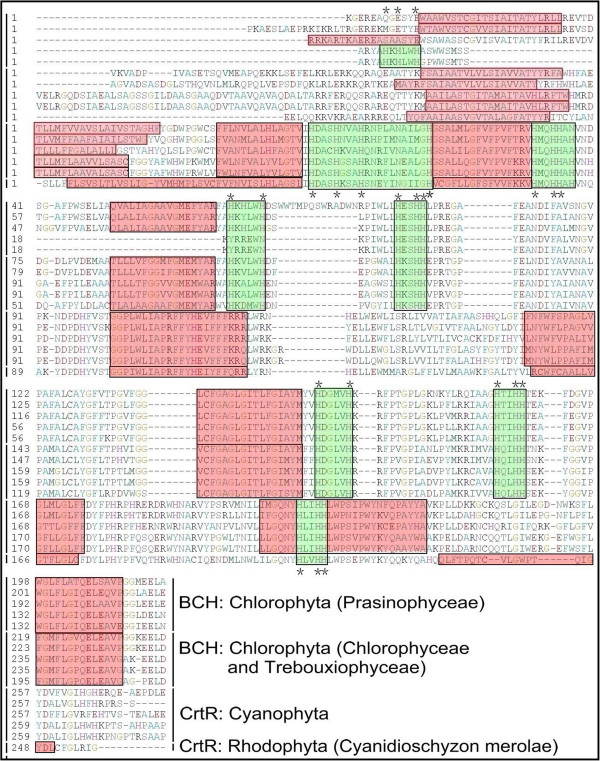
**Domain structure of BCH from green algae and CrtR from cyanobacteria and red algae.** Ten BCH-type CHYs from green algae, one CrtR-type CHY from red algae and five CrtRs from cyanobacteria are included. A partial protein sequence (position: 110–400) has been selected for domain structure analysis. The predicted trans-membrane segments are shaded in red. The histidine boxes are shaded in green and Black stars indicate the positions of conserved histidine residues. Ten BCH-type CHYs from green algae includes Prasionphyceae [*Micromonas pusilla*, *Micromonas* sp. RCC299, *Ostreococcus* sp. RCC809, *Ostreococcus tauri*, and *Ostreococcus lucimarinus*]. One CrtR-type CHY from the red alga is *Cyanidioschyzon merolae*. The information of BCH genes from algae is as in Table 1. Five CrtRs from cyanobacteria includes *Synechococcus* sp. JA-2-3B'a (2–13) [Cyanobase: CYB_0102], *Synechococcus* sp. JA-3-3Ab [Cyanobase: CYA_1931], *Cyanothece* sp. PCC 7425 [Cyanobase: Cyan7425_1008], *Acaryochloris marina* MBIC11017 [Cyanobase: AM1_3637] and *Thermosynechococcus elongatus* BP-1 [Cyanobase: tlr1900].

An alignment of the deduced amino acid sequences of CrtRs of cyanobacteria, and BCHs of red algae and green algae is displayed in Figure 2. There are few gaps in the alignment and a number of highly conserved regions. Four transmembrane segments and four histidine-rich boxes (HKXLWH, HXSHH, HDGLVH, and HXXHH) are distributed among the amino acid residues of BCHs of green algae (Figure 2). It is interesting that the first transmembrane segment was absent in two BCHs of green algae *O*. sp. RCC809 and *O. lucimarinus*, and replaced instead by another histidine-rich box in the N-terminal (HKHLWH). Three conserved histidine-rich boxes (HDASHXXAH, HXQHHXX, and HLIHH) and five transmembrane segments are generally well-conserved between red algae and cyanobacteria, indicating that red algae acquired this gene from cyanobacteria during primary endosymbiosis (Figure 2). However, previous studies have demonstrated that the protein sequence of the *C. merolae* BCH (CrtR-type) is truncated at the N-terminus by at least 22 amino acids relative to the sequences of all cyanobacterial CrtRs, and that the activity of this encoded enzyme could not be shown when expressed in *E. coli*[57,58]. Two HXXHH boxes are widespread across the BCHs of algae and CrtRs of cyanobacteria, indicating that their catalytic mechanisms are highly similar in these proteins [34,56].

### Origins and evolution of BCHs in algae

**Figure 3:**
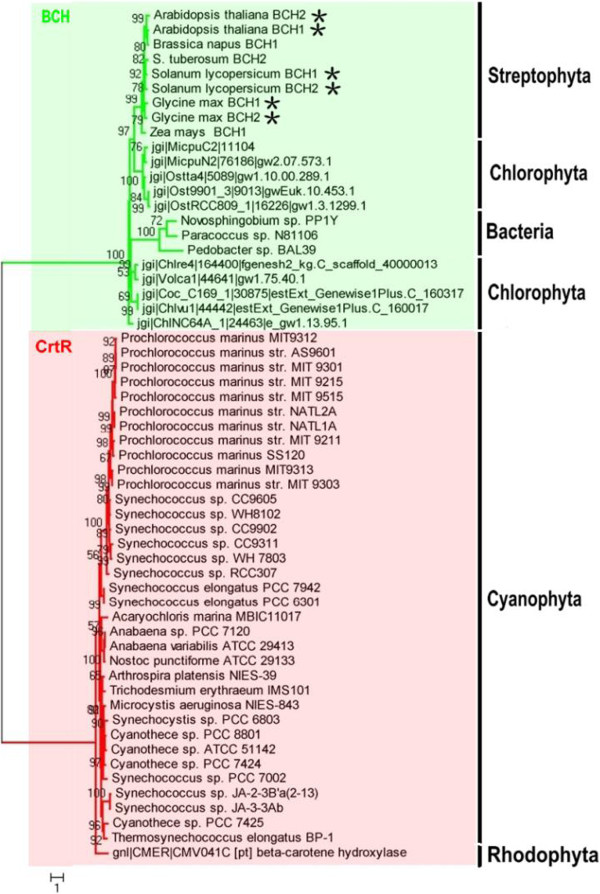
**A un-rooted maximum likelihood tree of our BCH database and some other BCHs from bacteria, higher plants and cyanobacteria.** The sequences information of BCH from cyanobacteria, bacteria and higher plants was downloaded from Cyanobase or NCBI database respectively and summarized in an additional file [see Additional file 3: Table S2]. A partial protein sequence (position: 110–400) has been selected for phylogenetic analysis. A maximum likelihood phylogenetic tree (loglk = −12176.58485) as inferred from amino acid sequences (291 amino acid characters) of BCH and CrtR proteins was computed using LG model for amino acid substitution (selected by PROTTEST) with discrete gamma distribution in four categories. All parameters (gamma shape = 1.963; proportion of invariants = 0.010; number of categories: 4) were estimated from the dataset. Numbers above branches indicate ML bootstrap supports. ML bootstraps were computed using the above mentioned model in 300 replicates. Stars indicate where later gene duplications led to creation of paralogs genes found within one species. Major groups of organisms are labeled to allow comparison between the phylogeny of BCH and algae evolution.

*β*–Carotene hydroxylation in different organisms is primarily carried out by members of three subfamilies of the non-heme/di-iron monooxygenase superfamily: BCH-type enzyme of green algae and higher plants; BCH-type enzyme of non-photosynthetic bacteria; and CrtR-type enzyme of cyanobacteria. The former shares sequence homology with non-photosynthetic bacterial-type enzyme, and the latter (CrtR-type) is more closely related to bacterial carotenoid ketolases [59]. Identification of plant-like BCHs in green algae and cyanobacteria-like CrtRs in red algae is the key indicating complicated origination of BCH in algae, for which we constructed a phylogenetic tree of CrtRs of cyanobacteria and BCHs of green algae, higher plants, and bacteria (Figure 3) (Additional file 3: Table S2).

As shown in Figure 3, difference between CrtRs of cyanobacteria and BCHs of algae, bacteria, and higher plants is very clear. Genes encoding BCHs from bacteria, green algae, and higher plants build a monophyletic group (Bootstrap [BS]: 100%), and the phylogenetic relationship present here indicates that they share a common ancestor and strongly supports a non-cyanobacterial origin. In contrast to the apparently widespread retention of BCH paralogs in higher plants, the gene encoding BCH is a single copy in each algal genome sequences (Figure 3). In higher plants, duplication of the ancestral *bch* gene took place most probably via whole-genome or segmental genome duplication [26] and the duplicates seem to have functionally diverged primarily at the gene expression level [60-62]. It is worth mentioned, however, that the gene encoding BCH (CrtR-type) of red algae forms another monophyletic group (BS: 97%) with CrtRs of cyanobacteria, suggesting that the red alga obtained this gene via the primary endosymbiotic event from cyanobacteria and retained it over the course of evolution persistently. However, the activity of this encoded enzyme was not demonstrated when expressed in *E. coli*[57,58]. Therefore, hydroxylation of *β*-carotene in red algae remains unclear, calling for more red-algal genomes sequences to elucidate. In this study, no BCH- or CrtR-type CHY-encoding gene was discovered in algae of Chromalveolates, except for a partial sequence of *bch*-type gene in the genome sequences of *T. pseudonana*. This result is consistent with observations from a previous study, in which only a partial sequence of BCH-type gene was found in the genome sequences of *T. pseudonana* and no one was detected from the *P. tricornutum* genome [51]. Unfortunately, the enzymatic activity of this putative carotene hydroxylases (BCH) from *T. pseudonana* has not yet been reported anywhere to the best knowledge of authors. Therefore, the hydroxylation of *β*-carotene in Chromalveolates remains unknown, resulting from genomes of the few organisms currently sequenced lack entire hydroxylation families and, enzymatic activity of putative carotene hydroxylases has not yet been reported at present [51,57,63-65].

As mentioned above, it is difficult to fully understand the evolution of *β*-xanthophylls biosynthesis with limited available data, but some hypotheses were proposed based on the tree topology. Firstly, it is presumed that CrtR-type CHY was originated in all algae as the result of primary endosymbiosis. In such a scenario, CrtR-type CHY proteins were lost in extant green algae and higher plants, and these organisms acquiring another BCH from bacteria by lateral gene transfer or from the host during the primary endosymbiosis event. Secondly, red algae have CrtR-type CHY-encoding gene, while Chromalveolates do not have them due possibly to gene lost during the secondary endosymbiosis event. In addition, BCH-type CHYs are also absent in Chromalveolates, which may be resulted from the replacement of algal CrtR- and BCH-type CHYs by another unidentified novel lineage-specific CHY in strains that derived from the red algal secondary endosymbiosis. Previous studies of the diatom *P. tricornutum*[43], however, have demonstrated the presence of a xanthophyll cycle (zeaxanthin-antheraxanthin-violaxanthin), with violaxanthin the putative precursor of both diadinoxanthin and fucoxanthin, implying this enzymatic reaction may therefore be catalyzed by other unrelated enzymes, such as LUT-like P450 proteins [51]. Therefore, we speculate that the *bch* genes in Chromalveolates have not been under a strong pressure of natural selection like in green algae and higher plant lineages, and they may have instead evolved different ways for *β*-xanthophylls biosynthesis.

### Domain structures of CYP97s in algae

**Figure 4:**
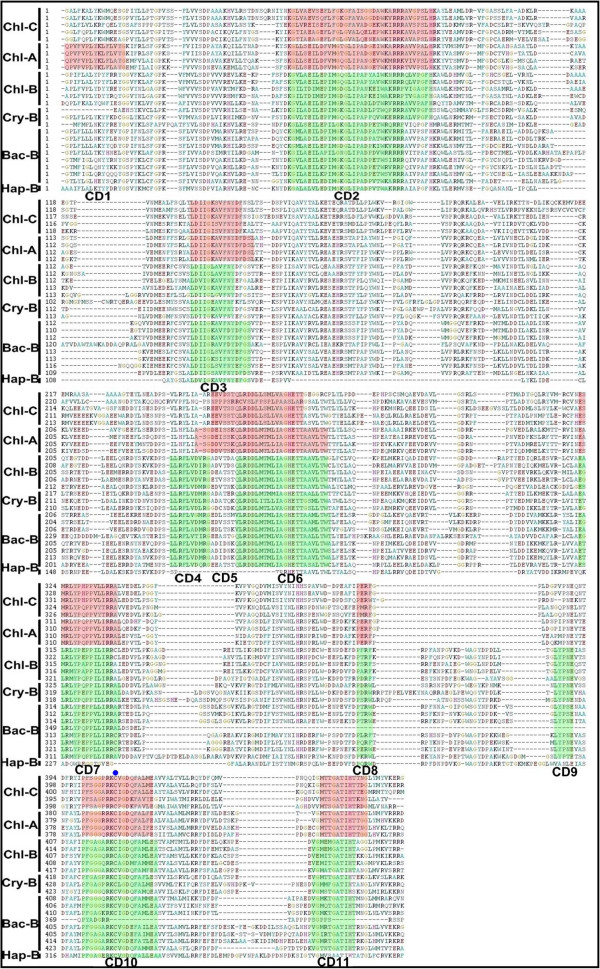
**Domain structure of CYP97 from algae.** A partial protein sequence (position: 272–926) has been selected for domain structure analysis. The red shades indicated conserved amino acid residues in CYP97A and CYP97C homologs from green algae. The green shades indicated conserved amino acid residues in all CYP97B homologs from all algae. The P450s active site components were found in the amino acid sequences of all CYP97A/B/C, including *I*-helix involved in oxygen binding (CD6 in CYP97B and CD5 in CYP97A and CYP97C), ERR triad (CD7 in CYP97A/B/C) involved in locking the heme pockets into position and to assure stabilization of the conserved core structure, and CD10 involved in heme binding and a conserved cysteine (the circle with blue color). The abbreviations used are: Chl-C, CYP97C from Chlorophyta *C. reinhardtii*, *V. carteri*, *M*. sp. RCC299, *O*. RCC809, and Cercozoa *B. natans* CCMP2755; Chl-A, CYP97A from Chlorophyta *C. reinhardtii*, *V. carteri*, *M*. sp. RCC299, and *O*. RCC809; Chl-B, CYP97B from Chlorophyta *M*. sp. RCC299, *O*. RCC809, and *V. carteri*; Cry-B, CYP97B from Cryptophyta *G. theta*; Bac-B, CYP97B from Bacillariophyta *T. pseudonana*, *P. tricornutum*, *F. cylindrus*, and Stramenopiles *A. anophagefferens*; Hap-B, CYP97B from *E. huxleyi*. The information of BCH genes from algae is as in Table 1.

Cytochrome P450s are defined by the 450 nm light absorption of their heme cofactors. They oxidize various arrays of metabolic intermediates and environmental compounds [66], and participate in many primary, secondary, and xenobiotic metabolic reactions [67]. The CYP97-encoding genes are members of P450s family. These members share a common catalytic center in heme by iron coordination to the thiolate of a conserved cysteine [68]. Despite low sequence identity at amino acid level, P450s display a common overall topology in three-dimensional folding pattern [69,70]. Genes encoding members of CYP97 family have been isolated and functionally investigated from higher plants [8,26,33,37,59,71,72]. There are three CYP97 family members (CYP97A3, CYP97B3, and CYP97C1) in *Arabidopsis* genome, of which CYP97A3 and CYP97C1 are predicted to be chloroplast-targeted [59,73]. The localization of predicted chloroplast CYP97A/C from algae and higher plants is coincident with the sub-cellular of carotenoids biosynthesis. To further reveal domain structure characteristics of CYP97 proteins, an alignment was constructed using selected CYP97A/B/C protein sequences from different algae (Figure 4) and an additional file shows this in more detail [see Additional file 4: Figure S1].

The alignment (Figure 4) reveals 11 domains in strongly conserved amino acid sequences from all CYP97 protein sequences. All conserved domains are highly similar between CYP97As and CYP97Cs of green algae except for CD1 (QPVFVPLYKLPLXYG) (Figure 4), indicating that they share a common ancestor. In addition, the conserved domains in CYP97Bs of distinct algae phyla are also similar, implying that this gene occurred before the formation of extant diverse algal groups. Degenerate primers for cloning genes encoding CYP97 homologs were designed from these conserved amino acid residues. P450s catalytic motifs were found as expected in all CYP97A/B/C protein sequences including *I*-helix involved in oxygen binding (CD6 in CYP97B and CD5 in CYP97A and CYP97C), an ERR triad (CD7 in CYP97A/B/C) responsible for locking the heme pockets into position and assuring stabilization of the conserved core structure, and CD10 associated with the conserved heme-binding cysteine. In addition, many amino acids were conserved in each CYP97 subfamily such as CD4 (LLRFLVDXR) and CD9 (LYPXE) were conserved in CYP97B subfamily only. The conserved domains associated with active sites suggest that these protein sequences are members of the P450 family, and that conserved domains within each CYP97 subfamily proteins are responsible for the specificity with respect to the *β*- or *ϵ*-ring of different carotenes.

### Origins and evolution of CYP97s in algae

Production of α-xanthophylls (lutein) in higher plants requires four reaction steps: *β*- and *ϵ*-ring formation from lycopene by the action of *β*- and *ϵ*-cyclases (LYCB and LYCE); and subsequently, hydroxylation of each ring of *α*-carotene by *β*- and *ϵ*-ring hydroxylases (CYP97A and CYP97C) [26]. Ample evidence shows that genes encoding CYP97A and CYP97C homologs are only present in green algae and higher plants, but not in Chromalveolates, indicating that *α*-xanthophylls biosynthesis occurs only in green algae and higher plant lineages. In our results, this biosynthetic pathway is also absent in red algae. In contrast to a previous study postulated that synthesis of α-xanthophylls occurs in only a few lineages of photosynthetic eukaryotes, namely, some red algae, all green algae, and higher plants [26]. An insufficient number of red algal species (only a red alga used in this study) may be responsible for these different conclusions. In addition, our previous and other studies have demonstrated that *β*-cyclases (LYCBs) genes are widely distributed in nature, and *ϵ*-cyclase (LYCEs) genes were identified only in green algae, higher plants, and some cyanobacteria (e.g. *Prochlorococcus marinus* MED4). LYCE genes seems to come from *β*-cyclases by gene duplication and subsequently functional divergence [30,57,74-77], which indirectly manifests that *α*-xanthophylls are synthesized only in green algae and higher plants. No CYP97A and CYP97C homologs are detected in algal genome sequences from Chromalveolates, and these algae cannot synthesize *α*-xanthophylls (lutein), which is consistent with previous studies [51].

**Figure 5:**
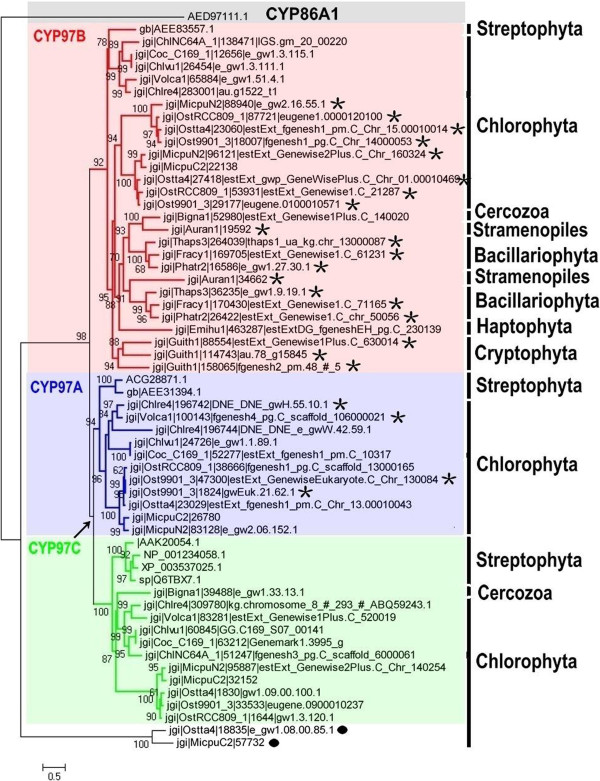
**A maximum likelihood tree of our CYP97 database and some other CYP97s from higher plants.** The sequences information of CYP97s from higher plants was downloaded from NCBI database and summarized as follow: *Arabidopsis thaliana* [GenBank: CYP97A3, gb|AEE31394.1, CYP97B3, gb|AEE83557.1, CYP97C1, sp|Q6TBX7.1 and CYP86A, AED97111.1], *Zea mays* [GenBank: CYP97A16, ACG28871.1], *Glycine max* [GenBank: carotene *epsilon*-monooxygenase, XP_003537025.1], *Solanum lycopersicum* [GenBank: CYP97C11, NP_001234058.1] and *Oryza sativa* Japonica Group [GenBank: carotene *epsilon*-monooxygenase, AAK20054.1]. A partial protein sequence (position: 272–926) has been selected for phylogenetic analysis. A maximum likelihood phylogenetic tree (loglk = −27808.68723) as inferred from amino acid sequences (655 amino acid characters) of CYP97 proteins was computed using LG model for amino acid substitution (selected by PROTTEST) with discrete gamma distribution in four categories. All parameters (gamma shape = 1.924; proportion of invariants = 0.011; number of categories: 4) were estimated from the dataset. Numbers above branches indicate ML bootstrap supports. ML bootstraps were computed using the above mentioned model in 300 replicates. The arrow indicates an ancient gene duplication event creating CYP97A/C, respectively. Stars indicate where later gene duplications led to creation of paralogs genes found within one species. Black circle indicate two genes belonged to no one subfamily of CYP97. Major groups of organisms are labeled to allow comparison between the phylogeny of CYP97A/B/C and algae evolution.

At present, no CYP97 protein homolog has been found in cyanobacteria, suggesting that these proteins are an ancient eukaryotic innovation. All three CYP97 subfamilies are represented in *Arabidopsis* and other land plants, often in a single copy per subfamily, indicating their critical functions [26]. In contrast, some paralogs genes encoding CYP97A in a few green algae and CYP97B in most algae, especially in Chromalveolates, indicating recent gene duplication events occurred. For deep understanding the internal phylogenetic relationships among CYP97 proteins from higher plants and green algae, we constructed a maximum likelihood tree using our CYP97 database and some other CYP97s from higher plants (Figure 5). CYP86A1 from *Arabidopsis thaliana* was selected as outgroup because its substrates, fatty acids with chain lengths from C_12_ to C_18_[78] are mostly molecules similar to carotenoids, and the CYP86 clade is the most closely related to the CYP97 clade [26].

As it shows in maximum likelihood tree, CYP97 proteins constitute three distinct monophyletic groups: CYP97A, CYP97B, and CYP97C. Two of these groups (CYP97A and CYP97C) form a sister group that are composed of proteins from green algae and higher plants. CYP97A and CYP97C sequences in Chromalveolates, including Heterokontophyta (*P. tricornutum, F. cylindrus*, and *T. pseudonana*), Haptophyta (*E. huxleyi*), Cryptophyta (*G. theta*), and Stramenopiles (*A. anophagefferens*) are absent in the cluster. Genes encoding CYP97B homologs from all organisms form a monophyletic group (BS: 92%). Surprisingly, algal CYP97Bs from Chromalveolates and green algae (Prasinophyceae) build another monophyletic group (BS: 95%). This phylogenetic relationship and the lack of CYP97B homologs in *C. merolae* point to a “green” origin of this gene in Chromalveolates, similar to the origin and evolution of hydroxypyruvate reductase [79]. Alternatively, this gene represents early eukaryotic innovations in the Plantae although lacking CYP97B homolog in *C. merolae*. Further investigations are needed to support this hypothesis.

The topology of the phylogenetic tree shows that the CYP97B is an ancient gene emerging before the divergence of extant algae groups during evolution, and represents an ancient eukaryotic innovation. Our results indicate that CYP97A is evolutionarily more closely to CYP97C than to CYP97B (Figure 5). Therefore, we believe that the CYP97A and CYP97C genes were originated by gene duplication before the split between green algae and higher plants, and were subjected to purifying selection in a lineage-specific fashion (Figure 5). Alternatively, these genes may have been originally present in all algae, and then they were lost gradully from red algae and Chromalveolates. This scenario is similarity to that of *Arabidopsis* whose evolution and functional divergence of two duplicate gene pairs, CYP97A3/C1 and BCH1/2 involved in carotenoid hydroxylation occurred [26]. Additional lineage- or organism-specific gene duplications have occurred during the evolution of CYP97A in green algae (*C. reinhardtii* and *O. lucimarinus*), and CYP97B in most algae, except for *C. reinhardtii*, *C.* sp. NC64A, *C. vulgaris*, *C.* sp. C-169, *V. carteri*, *M. pusilla*, *E. huxleyi*, and *B. natans* CCMP2755. Duplication and subsequent functional divergence of genes have been recognized increasingly as an important mechanism of evolution [80-83].

### Isolation and characterization of *cyp97* genes from *H. pluvialis*

To further study genes encoding CYP97 homologs in green algae, three full-length cDNA sequences of *cyp97* homologs, including *Haecyp97a*, *Haecyp97b*, and *Haecyp97c*[84] were isolated from commercial green alga *H. pluvialis* strain Flotow 1844. Briefly, 1,017- and 984-bp cDNA fragments encoding HaeCYP97A/B were generated by RT-PCR with degenerate primers [see Additional file 5: Table S3]. The *Haecyp97a* fragment shared 77% sequence similarity with *cyp97a5* gene of *C. reinhardtii* and 74% similarity with *cyp97a3* gene of *A. thaliana*. The *Haecyp97b* fragment shared 61% sequence similarity with *A. thaliana cyp97b3* gene. The results indicate that two partial putative *cyp97a/b* genes were isolated from *H. pluvialis*. Gene-specific primers were then designed to obtain full-length sequences of *Haecyp97a/b* using RACE methods. Information regarding the full-length sequences of *Haecyp97a/b/c* is summarized in Table 2. The full-length cDNA sequences of *Haecyp97a* comprised 1,872-bp with an open reading frame (ORF) of 1,593-bp encoding a 530 amino acid protein, and it was flanked by a 159-bp of 5′-untranslated region (UTR) and a 120-bp of 3′-UTR including the poly-A tail [see Additional file 6: Figure S2]. The deduced protein had a calculated molecular weight of 59.03 kDa and a predicted isoelectric point (pI) of 7.81. The full-length cDNA sequences of *Haecyp97b* contained an ORF of 1,620-bp, a 2-bp 5′-UTR, and a 249-bp 3′-UTR, and it encoded a new putative carotenoid hydroxylase protein of 539 amino acid protein with a deduced molecular weight of 58.72 kDa and pI of 6.26 [see Additional file 7: Figure S3]. Sequence analysis revealed that the cloned *Haecyp97c* cDNA was 1,995-bp in length, and contained a 1,620-bp ORF, a 46-bp 5′-UTR, and a 329-bp 3′-UTR in characteristic of a poly (A) tail. An ATG translation initiation codon was identified in the 46-bp terminal sequence (47-49 bp), and a TAA termination codon was found in the 1,620 bp downstream of the initiation site [see Additional file 8: Figure S4].

**Table 2:** **Listed of three full-length of HaeCYP97A/B/C from *Haematococcus pluvialis***

	**mRNA (bp)**	**5′UTR (bp)**	**CDS (bp)**	**3′UTR (bp)**	**Protein (aa)**	**MW (kDa)**	**pI**	**TM**	**SL**
HaeCYP97A	1872	1-159	160-1752	1753-1872	530	59.03	7.81	-	chl
HaeCYP97B	1871	1-2	3-1622	1623-1871	539	58.72	6.26	-	nd
HaeCYP97C	1995	1-46	47-1666	1667-1995	539	58.71	7.94	-	chl

Abbreviations: *5’UTR* 5’-untranslated region, *3’UTR* 3’-untranslated region, *CDS* coding sequences, *Mw* molecular weight, *pI* isoelectric point, *TM* Transmembrane regions, *SL* subcellular localization, *bp* base pair, *chl* chloroplast.

The ChloroP and TargetP servers [85,86] were used to predict the sub-cellular location of the respective deduced proteins from isolated genes (Table 2). The results indicate that CYP97A and CYP97C in *H. pluvialis* are probably located in the chloroplast, same as in higher plants *Arabidopsis thaliana*[59,73]. We also calculated degrees of identity and similarity between predicted amino acid sequences of each isolated CYP97 and corresponding CYP97s of other eukaryotes (Table 3). Amino acid sequences from each of the isolated CYP97 genes shared high similarities (72%-76%) with known eukaryotic proteins, indicating that the three novel *cyp97* genes of green alga *H. pluvialis* Flotow 1844 has been successfully identified using RT-PCR with degenerate primers designed from conserved motifs and RACE methods. To our knowledge, this is the first time these three *cyp97* gene homologs have been isolated from commercial green algae.

**Table 3:** The degree of identity and similar between the predicted amino acid sequences for each isolated CYP97 genes and corresponding CYP97 from other eukaryotes

	**HaeCYP97A**	**HaeCYP97B**	**HaeCYP97C**	**AthCYP97A3**	**AthCYP97B3**	**AthCYP97C1**
HaeCYP97A	100/100	51/66	46/64	57/72	48/64	50/70
HaeCYP97B		100/100	43/59	48/63	61/76	44/60
HaeCYP97C			100/100	46/62	39/56	61/72
AthCYP97A3				100/100	40/65	53/72
AthCYP97B3					100/100	43/60
AthCYP97C1						100/100

Note: AthCYP97A3 [GenBank: NP_564384.1], AthCYP97B3 [GenBank: NP_193247.2] and AthCYP97C1 [GenBank: NP_190881.2] were downloaded from National Center for Biotechnology Information GenBank database, respectively. The data was present by the form of identity/similarity.

### Expression analysis of four *chy* genes in *H. pluvialis* under high light stress

Studies showed that high light (HL) effectively induces carotenoid biosynthesis-related gene expression and astaxanthin accumulation in *H. pluvialis*[47,48], indicating that light plays an important role in controlling green algal carotenoid biosynthesis. Although the regulatory role of light in the expression of nuclear-encoded plastid-targeted proteins has been studied for decades in green algae and higher plants, elucidation of effects of light on transcriptional levels of three novel *cyp97* genes in green algae is still in its infancy. In a previous study on diatom *P. tricornutum*, a blue-light library was found to be the most enriched in carotenogenesis-related ESTs [51]. For more detail in the transcriptional regulatory role of light on green algae, we studied the gene transcriptional expression profiles of *bch*, *Haecyp97a*, *Haecyp97b*, and *Haecyp97c* genes in response to white and blue HL conditions.

**Figure 6:**
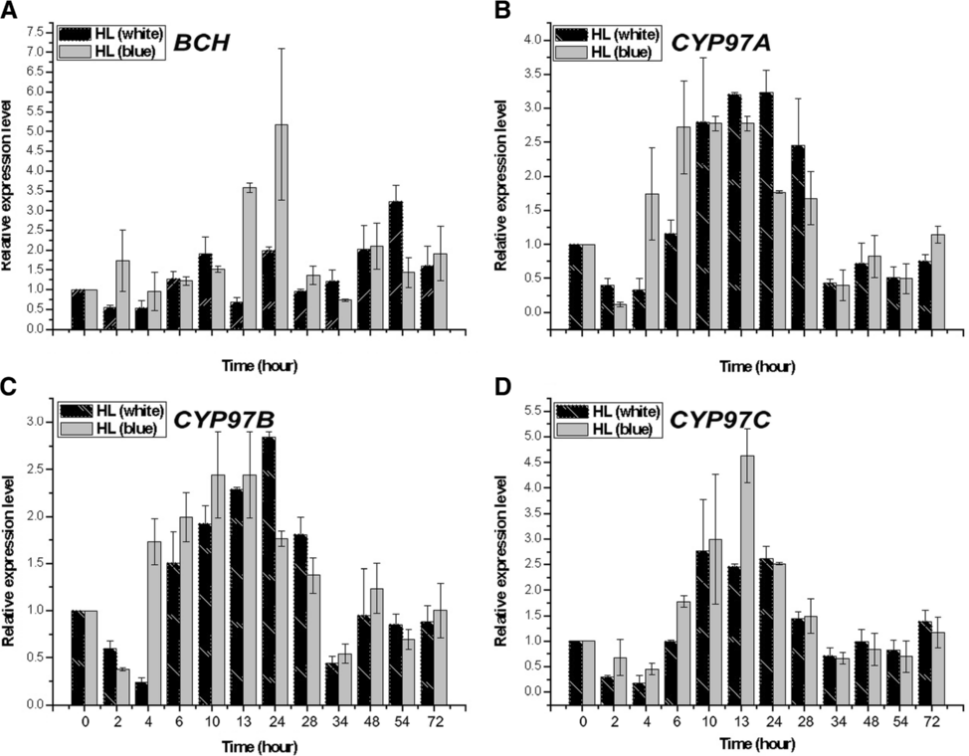
**mRNA levels of xanthophylls biosynthesis-related genes upon white or blue high light stimulation.** The exponentially growing cultures (cell density approximately 5 × 10^7^ cells ml^–1^) were harvested and transferred cells to 500-ml erlenmeyer flasks (named, 1–9), each containing 250-ml BBM (fresh medium) under continuous white light (390–770 nm) or blue light (420–500 nm) with light intensity of 1,000 μmol photons m^–2^ s^–1^ without a day/night cycle, respectively. Collected algal cells (20-mL sample at some selected time) were rinsed with PBS, stored at −80°C if not immediately used. The relative transcript levels of *bch***[A]**, *Haecyp97a***[B]**, *Haecyp97b***[C]** and *Haecyp97c***[D]** were determined after 2, 4, 6, 10, 13, 24, 28, 34, 48, 54 and 72 h by qRT-PCR using *actin* as a reference gene. The values were normalized to the transcript levels in the normal light condition. Data are averages of triplicate measurements. The error bars represent standard deviation. Length of the distance in x-axis did not correspond to length of induced time (hours).

As shown in Figure 6, transcriptional levels of four *chy* genes (*bch*, *Haecyp97a*, *Haecyp97b*, and *Haecyp97c*) were increased throughout the course of HL illumination treatments. Starting from relatively low levels, *bch* expression level was slowly increased under blue HL treatment and reached a maximum transcriptional level at 24 h exposure that was 5.0-fold higher than that of the control. It then declined sharply after 72 h of exposure (Figure 6A). A similar, although less pronounced trend was observed under white HL: the highest *bch* transcriptional level occurred at 54 h of exposure, with 3.3-fold higher compared with the control. Our previous study has demonstrated that zeaxanthin concentrations under blue and white HL treatments were increased markedly and reached their highest levels at 34 h (blue) and 48 h (white) of exposure [84], which is listed in Figure S5 for details [see Additional file 9: Figure S5]. BCH is the mainly enzyme catalyzing the hydroxylation of *β*-carotene, which produces zeaxanthin associated with the xanthophylls cycle [40,87]. The contradiction between low *bch* expression levels and marked increase of zeaxanthin concentration during the early stage of treatments was explained by the fact that when photoprotection is required, violaxanthin is rapidly converted via antheraxanthin to zeaxanthin by violaxanthin de-epoxidase [88]. The astaxanthin concentration under blue or white HL stress was higher than that of the control, reaching a maximum level at 34 h and 54 h of exposure, respectively [84], which is listed in Figure S5 for details [see Additional file 9: Figure S5]. Accumulation of astaxanthin may be responsible for the rapidly increasing levels of *bch* and sharply decreasing concentrations of zeaxanthin at later treatment stages. Previous studies have demonstrated that *bch* and *bkt* are the main genes involved in astaxanthin biosynthesis [89].

Expression levels of *Haecyp97a*, *Haecyp97b*, and *Haecyp97c* began with a slight decrease followed by a dramatic increase (Figure 6B-D). Strongly steady increase in transcriptional levels of *Haecyp97a* and *Haecyp97b* were observed during 4–28 h of both HL (white and blue) treatments. Then, they dropped sharply after 28 h of exposure. It is also intriguing that different conditions had various impacts on *Haecyp97a* and *Haecyp97b* mRNA levels. For instance, transcriptional levels of *Haecyp97a* and *Haecyp97b* under blue HL were higher than that of under white HL during 4–10 h of exposure, and the contrary tendency was observed during 13–28 h of exposure (Figure 6B and C). The maximum transcriptional levels of *Haecyp97c* under both the blue and white HL treatments occurred at hour 13 and 10, and transcriptional levels were 4.5- and 2.8-fold higher than that of control, respectively (Figure 6D). Blue HL appeared to have stronger effects on *Haecyp97c* transcriptional level than that of white HL (Figure 6D). Studies on mutant of higher plants showed that CYP97A3 and CYP97C1 are the enzymes primarily responsible for catalyzing hydroxylation of *β*- and *ϵ*-ring of *α*-carotene, respectively, producing *α*-branch xanthophylls (lutein), the most abundant carotenoids in light-harvesting complexes (LHCs) which is key structural and functional components of light harvesting [6,8,26,33,37,39,59,72]. During early stages (0–4 h) of HL (blue and white) treatments, lutein concentrations were lower than that of the control [84], which is listed in Figure S5 for details [see Additional file 9: Figure S5]. This result is consistent with the decreased transcriptional levels of *Haecyp97a* and *Haecyp97c* observed in *H. pluvialis* (Figure 6B and D). Although *Haecyp97a* and *Haecyp97c* transcriptional levels increased over time, lutein concentration level remained rather stable but was lower than the control. When considering that CYP97A is also involved in hydroxylation of *β*-carotene into zeaxanthin, the level of *Haecyp97a* increase is understandable [26]. Previous studies have demonstrated there was a cytochrome P450 involved in astaxanthin biosynthesis in *H. pluvialis* by the use of ellipticine [90]. Therefore, we speculate that CYP97A may be the cytochrome P450 involved in astaxanthin biosynthesis in *H. pluvialis*. According to our results, however, it is unclear why and how the levels of *Haecyp97b* and *Haecyp97c* increased. The function of CYP97B is currently unknown and they (CYP97B and CYP97C) may play some additional roles (aside from CHY *ϵ*–ring) in algae under adverse conditions. An earlier study reported that cytochrome P450 reductase is also co-up-regulated with enzymes involved in DNA repair under light/dark cycles [91].

Our data (Figure 6) reveal that mRNA levels of different *chy* genes increase rapidly under HL exposure and varied with light wavelength. For instance, our results indicate that blue HL very significantly increased *Haebch* and *Haecyp97c* genes expression, and produced similar effects on *Haecyp97a* and *Haecyp97b* genes expression compare with white HL treatment. Young et al. [40] have demonstrated that different carotenoid metabolic gene isoforms typically had distinctly different expression patterns. Previous study has demonstrated that zeaxanthin and astaxanthin concentrations were more efficiently enhanced for *H. pluvialis* under blue HL treatment than white [84]. Therefore, we propose that *Haematococcus* cells are more sensitive to blue induction compare with white. Similar phenomena were reported red Light Emitting Diodes (LEDs) operated at a relatively low light intensity were found to be suitable for cell growth; and LEDs emitting short wavelength (380–470 nm) can induce morphological changes in *H. pluvialis* and enhance astaxanthin accumulation [92-94]. It is well known that strong light (blue) enhances astaxanthin accumulation, because astaxanthin is produced by *H. pluvialis* cells to protect cells from the strong intensity of the light [93]. Although blue light contains more energy than red light, according to Planck’s law [95], in fact only energy associated to the S1 transition level of chlorophyll (Chl) can be used for photosynthesis [96]. On this bottom line, the fact that the efficiency of energy transfer from carotenoid to Chl a is far lower (40%) with respect to Chl b to Chl a or Chl a to Chl a. Thus it can easily be concluded that less energy used for photosynthesis is available from blue light than from red or white light under the same light intensity. According to above conclusions, we speculate that astaxanthin accumulation for *H. pluvialis* depends on not only light intensity, but also light quality (i.e. blue light might be a more useful wavelength to enhance astaxanthin accumulation in *H. pluvialis* under HL intensity). Therefore, we believe that the response of *H*. *pluvialis* Flotow 1844 to high light stress is a complicated process involving *bch*, *Haecyp97a*, *Haecyp97b*, and *Haecyp97c*.

## Conclusions

Our study provided a genome-wide comparative analysis of genes encoding CHYs in algae, with a focus on their distribution, structure, evolution, origins, and expression. As part of this study, we provided a summary of currently known distributions of *chy* genes in eukaryotic photosynthetic algae, and constructed hypotheses regarding xanthophylls biosynthetic pathways. Genes encoding BCHs of green algae and higher plants were determined to be non-cyanobacterial origin, whereas those from red algae were derived from cyanobacteria. Genes involved in *β*-xanthophylls biosynthetic pathway in Chromalveolates remains unknown. CYP97B genes are likely an innovation of eukaryotic algae, whereas genes of CYP97A and CYP97C initiated by gene duplication events before the split of green algae and higher plants. The biosynthesis of *α*-xanthophylls is characteristic of green algae and higher plants. Although transcriptional levels of *bch*, *Haecyp97a*, *Haecyp97b*, and *Haecyp97c* were up-regulated by blue and white HL treatments, the amplitude and kinetics of mRNA accumulation varied among different *chy* genes. Compare with white light, blue light may play a more important role in controlling green algal carotenoid biosynthesis. The response of *H. pluvialis* Flotow 1844 to different HL stresses is a complicated course involving *bch, Haecyp97a, Haecyp97b*, and *Haecyp97c*. The observed increase in *Haecyp97b* transcriptional level implies that this gene may be another carotenoid biosynthetic-related gene, perhaps a novel *chy* gene in Chromalveolates. Further investigations are needed to test this hypothesis.

## Methods

### Identification of *chy* genes encoding CHY proteins

The genomes of 18 eukaryotic photosynthetic algae included *Chlamydomonas reinhardtii*, *Chlorella* sp. NC64A, *Chlorella vulgaris*, *Coccomyxa* sp. C-169, *Volvox carteri*, *Micromonas pusilla*, *Micromonas* sp. RCC299, *Ostreococcus* sp. RCC809, *Ostreococcus tauri*, *Ostreococcus lucimarinus*, *Phaeodactylum tricornutum*, *Thalassiosira pseudonana*, *Fragilariopsis cylindrus*, *Aureococcus anophagefferens*, *Emiliania huxleyi*, *Guillardia theta* and *Bigelowiella natans* CCMP2755 were obtained from the website of the DOE Joint Genome Institute (Walnut Creek, CA, USA; http://genome.jgi.doe.gov/). The genome of the red alga *Cyanidioschyzon merolae* was obtained from the *C. merolae* Genome Project (http://merolae.biol.s.u-tokyo.ac.jp). The protein coding sequences of each genome was fed into the program makeblastdb to create an organism-species database [97].

Two methods were applied to identify the putative CHY homologs genes. Firstly, we followed JGI’s or the *C. merolae* Genome Project’s annotation to determine the number of *chy* present in the algal genomes. Then, eight previously characterized CHYs from *Haematococcus pluvialis* [GenBank: BCH, ABB70496.1], *Chlamydomonas reinhardtii* [GenBank: BCH, AAX54907.1], *Synechocystis* sp. PCC 6803 [GenBank: CrtR, BAA17468.1] and *Arabidopsis thaliana* [GenBank: BCH1, sp|Q9SZZ8.1; BCH2, sp|Q9LTG0.1; CYP97A3, NP_564384.1; CYP97B3, NP_193247.2, and CYP97C1, NP_190881.2] were used to construct a query protein set. BLASTp [97,98] and HMMER [99] programs were then conducted locally to identify all *chy* genes in all 18 algal genomes using a threshold e-value of 1e-10. Finally, we manually checked the extracted proteins by SMART and Pfam analyses to avoid false positive hits that commonly arise during large-scale automated analyses. Putative *chy* genes found by this method were added to the query set for another round of BLASTp searches. This procedure was iterated until no newly retrieved sequences that belonged to *chy* homologs. Moreover, in order to check for false negatives, two HMM models [Pfam: PF04116] and [Pfam: PF00067] derived from known *bch* and *cyp97* genes were applied to search for genes encoding *chy* on all proteins encoded in the 18 algal genomes [100,101]. All translated protein sequences of CHYs-encoding genes used in this paper were listed in more detail [see Additional file 2]. CHY proteins from higher plants and cyanobacteria were also downloaded from National Center for Biotechnology Information GenBank database and Cyanobase (http://bacteria.kazusa.or.jp/cyanobase/).

### Multiple sequence alignment and phylogenetic analysis

Proteins identified by the BLAST and HMM searches were aligned using ClustalW [102,103] with a gap opening penalty of 10, a gap extension penalty of 0.2, and Gonnet as the weight matrix. The SMART [104] and Pfam 26.0 [105] databases were applied to delete false positives. Phylogenetic trees were constructed using Maximum likelihood (ML) method that implemented in PhyML [106]. ML trees were built in particular models of amino acid substitutions chosen according to PROTTEST AIC results [107]. The Le and Gascuel evolutionary model [108] was selected to analyze the protein phylogenies by assuming an estimated proportion of invariant sites and a gamma correction (four categories). Bootstrap values (BS) were inferred from 400 replicates. Graphical representation and edition of the phylogenetic tree were performed with TreeDyn (v198.3) [109].

### Algal strains and culture conditions

*H. pluvialis* strain Flotow 1844 was obtained from Culture Collection of Algae and Protozoa, Dunstaffnage Marine Laboratory, and maintained at the Biological Resources Laboratory, Yantai Institute of Costal Zone Research, Chinese Academy of Science. Algae were incubated in 250-ml erlenmeyer flasks, each containing 100-ml BBM, and placed in an illuminating incubator (Ningbo Jiangnan Instrument Factory, GXZ-380, Ningbo, China) under a light intensity of 25 μmol photons m^–2^ s^–1^ in 14 h:10 h diurnal scheme at temperature of 22 ± 1°C without aeration.

For high light stress conditions, exponentially growing cultures (cell density approximately 5 × 10^7^ cells ml^–1^) were harvested, and then transferred to 500-ml erlenmeyer flasks (named, 1–9), each containing 250-ml BBM (fresh medium) under continuous exposure of white light (390–770 nm) or blue light (420–500 nm) in light intensity of 1,000 μmol photons m^–2^ s^–1^ without day/night cycle. Algal cells collected (20-mL sample at some selected time) were rinsed with PBS, stored at −80°C if not immediately used. All experimental chemicals and reagents were of analytical grade.

### Cloning and characterization of the CYP97 genes

*H. pluvialis* strain flotow 1844 in the exponential growth phase were harvested. Total RNA was extracted from fresh cells of *H. pluvialis* using Trizol reagent (TaKaRa D9108B, Dalian, China) according to the user manual. RNA solutions were stored at −80°C, if not immediately used. First-strand cDNAs were synthesized from 2 μg total RNA with PrimeScript® RT Enzyme Mix I (TaKaRa DRR047A, Dalian, China) according to the manufacturer′s instructions.

The CODEHOP (Consensus-degenerate hybrid oligo-nucleotide primers) strategy (http://blocks.fhcrc.org/blocks/codehop.html) was used to generate degenerate primers [110,111] for the cloning of core partial sequences of HaeCYP97A/B/C basing on the highly conserved regions of predicted putative genes encoding CYP97 homologs from some green algae. The nucleotide sequences of the 3’- and 5’-ends of HaeCYP97A/B/C were amplified by the RACE method [112,113]. Gene-specific primers were designed from the amplified core cDNA sequence of HaeCYP97A/B/C and their 3’- and 5’-ends were obtained using SMARTTM RACE cDNA Amplification Kit (Clontech) according to the manual. All primers used in this study were listed in more [see Additional file 5: Table S3]. The PCR products were resolved by electrophoresis on 1% agarose gel. The fragment of interest was excised and purified using agarose gel DNA fragment recovery kit (TaKaRa D823A, Dalian, China). Finally, the fragment was cloned into PMD-18T vector (TaKaRa D101A, Dalian, China) and sequenced (Invitrogen, Beijing, China).

The full-length cDNA sequence of HaeCYP97A/B/C was spliced according to the RACE-PCR results by the SeqMan software of DNAStar 7.1 (DNASTAR Inc., USA). The theoretical molecular weight (Mw) and isoelectric point (pI) of HaeCYP97A/B/C protein were computed by ExPASy Compute pI/Mw tool [114]. Transmembrane regions were predicted by "DAS"-Transmembrane Prediction server [115]. Prediction of sub-cellular localization of the deduced amino acids was conducted using ChloroP and TargetP Servers [82,83].

### Gene expression profiling: Real-time RT-PCR

Total RNA from different samples was extracted using Trizol reagent (TaKaRa D9108B, Dalian, China) according to the user manual. Total RNA was treated with RNase-free DNase I (Fermentas, Glen Burnie, MD) to remove any residual genomic DNA that might be carried through the extraction process. Nuclear acids were quantified by NanoDrop 2000c (Thermo Scientific, USA). The first-strand cDNA synthesis for quantitative Real-time RT-PCR (qRT-PCR) was obtained from 1 μg total RNA with PrimeScript® RT Enzyme Mix I (TaKaRa DRR047A, Dalian, China) according to the manufacturer’s instructions.

Gene-specific primers were designed with Primer5 and are listed in more [see Additional file 5: Table S3]. All primer pairs were initially tested by standard RT-PCR and the amplification of single products of the correct size was verified on 2% (w/v) agarose gels (data not shown). The *actin* gene [116] has been proven experimentally in this study [see Additional file 10: Figure S6]. The qRT-PCR amplifications were carried out in triplicate in a total volume of 20 μL according to the manufacturer’s instructions of SYBR® Premix Ex Taq™ (Tli RNaseH Plus) (TaKaRa DRR420A, Dalian, China). The qRT-PCR program was holding stage, 50°C for 20 s and 95°C for 10 min, followed by 40 cycles of 95°C for 15 s, 60°C for 1 min, and melt curve stage, 95°C for 15 s, 60°C for 1 min, 95°C for 30 s, and 60°C for 15 s. Real-time RT-PCR analysis was performed on an ABI fast 7500 Sequence Detection System (Applied Biosystems) following the protocol previously described using *actin* gene as the internal control. The relative steady state mRNA transcript levels were normalized to the respective *actin* transcripts. The 2^–ΔΔCT^ method [117] was used to analyze quantitative real-time PCR data based on the cycle threshold (C_*T*_) values. The ΔΔC_*T*_ is represented as the following formula: ΔΔC_*T*_ = (C_*T*_, target gene (test group) - C_*T*_, *actin* gene (test group)) time x - (C_*T*_, target gene (control group) - C_*T*_, *actin* gene (control group)) times x, where x is the time of selected sample. Therefore, the relative expression level of *chy* genes has been normalized to control group (normal light regime).

### Statistical analyses

All exposure experiments were repeated three times independently, and data were recorded as the mean with standard deviation (SD). For gene expression experiments, quantitative real-time PCR analysis was performed using software BioRAD iQ5. For each gene, the expressed as the mean ± SD (% control) was calculated using the (standard curve) approximation corrected for primer efficiency and normalized to housekeeping gene *actin* expression values.

### Nucleotide sequencing and accession numbers

The cDNA nucleotide sequences of HaeCYP97A/B/C have been deposited and assigned the accession number AFR31786, AFR36909, and AFQ31612, respectively, in the EMB/GenBank/DDBJ database.

## Abbreviations

CHY: Carotenoid hydroxylase; BCH: Non-heme/di-iron *β*-ring hydroxylase; P450s: Cytochrome P450 proteins; CYP97: Cytochrome P450 protein, 97 family; CD: Conserved domain; BS: Bootstrap value; RACE: Rapid Amplification of cDNA Ends; BKT: *β*-Carotenoid ketolase; HL: High light.

## Competing interests

The authors have declared that no competing interests exist.

## Authors' contributions

HLC and XNY conceived of the study, participated in all the bioinformatics analysis, including the sequence alignment and phylogeny analysis and drafted the manuscript; YLC helped in bioinformatics analysis, data mining and management; XQL and YW conceived the idea of identification of three full-length of CYP97 genes and designed the study; ZPL and SQ guided the writing of the manuscript. All authors read and approved the final manuscript.

## Supplementary Material

Additional file 1: Table S1 The details about the completeness of genome sequences used in this study. The details about the completeness of genome sequences used in this study were summarized from (DOE Joint Genome Institute, project list: http://genome.jgi.doe.gov/genome-projects/). The genomes of the red alga *Cyanidioschyzon merolae* was obtained from the *C. merolae* Genome Project (http://merolae.biol.s.u-tokyo.ac.jp). Click here for file

Additional file 2 **The deduced protein sequences of genes encoding CHYs from 18 algal genomes.** The red indicated genes encoding BCH-type CHYs in algal genomes. The blue indicated two genes encoding proteins which belong to no one subfamily of CYP97. Click here for file

Additional file 3: Table S2 The sequences information of BCHs from higher plants and CrtRs from cyanobacteria was downloaded from NCBI database or Cyanobase respectively. Click here for file

Additional file 4: Figure S1 The multiple sequence alignment of all CYP97 from algae. The names of each sequence are as listed in Additional file 2. A partial protein sequence (position: 272–926) has been selected for domain structure analysis. Click here for file

Additional file 5: Table S3 List of primer sequences used for PCR amplification to clone three full-length cDNA of CYP97A/B/C homologs respectively in green alga *Haematococcus pluvial*is strain Flotow 1844. Note: F, forward; R, reverse; Position, the location of motifs where the primers were designed from protein multiple sequence alignments. Click here for file

Additional file 6: Figure S2 Nucleotide and the predicted amino acid sequence of HaeCYP97A. Click here for file

Additional file 7: Figure S3 Nucleotide and the predicted amino acid sequence of HaeCYP97B. Click here for file

Additional file 8: Figure S4 Nucleotide and the predicted amino acid sequence of HaeCYP97C. Click here for file

Additional file 9: Figure S5 Changes in total chlorophylls and carotenoids concentrations of *H. pluvialis* under different high light stresses (white and blue). Cells grown in autotrophic medium were harvested and transferred to fresh medium with different high light intensity. Cells were harvested at different periods of induction and changes in total chlorophylls (A), total chlorophylls (B), lutein (C), α-carotene (D), β-carotene (E), and astaxanthin (F). Values are mean ± SD of three independent determinations. Click here for file

Additional file 10: Figure S6 The transcriptional level of *actin* gene under high light stress of different wavelength (blue and white). Click here for file
