# Supplementary material for: Evolutionary origins, molecular cloning and expression of carotenoid hydroxylases in eukaryotic photosynthetic algae
Source: BMC Genomics. 2013 Jul 8;14:457. doi: 10.1186/1471-2164-14-457 (PMC3728230; doi:10.1186/1471-2164-14-457)
Supplement: Additional file 1: Table S1 — The details about the completeness of genome sequences used in this study. The details about the completeness of genome sequences used in this study were summarized from (DOE Joint Genome Institute, project list: http://genome.jgi.doe.gov/genome-projects/). The genomes of the red alga Cyanidioschyzon merolae was obtained from the C. merolae Genome Project (http://merolae.biol.s.u-tokyo.ac.jp). [file 1471-2164-14-457-S1.pdf]

Evolutionary origins, molecular cloning and expression of carotenoid hydroxylases in eukaryotic photosynthetic algae

Hongli Cui[1, 2§], Xiaona Yu[3§], Yan Wang[2], Yulin Cui[2], Xueqin Li[4], Zhaopu Liu[3] and Song Qin[1*]

[1]Key Laboratory of Coastal Biology and Biological Resources Utilization, Yantai Institute of Coastal Zone Research, Chinese Academy of Sciences, Yantai 264003, People's Republic of China

[2]University of the Chinese Academy of Sciences, Beijing 100049, People's Republic of China

[3]College of Resources and Environmental Sciences, Key Laboratory of Marine Biology, Nanjing Agricultural University, Nanjing 210095, People's Republic of China

[4]Shenzhen Key Laboratory for Marine Bio-resource and Eco-environment, College of Life Sciences, Shenzhen University, Shenzhen 518060, People's Republic of China

[§]These authors contributed equally to this work.

[*]Corresponding author

E-mail addresses:

HLC: hlcui@yic.ac.cn

XNY: 2011103006@njau.edu.cn

YW: ywang@yic.ac.cn

YLC: yulincui@yic.ac.cn

XQL: 2110180316@email.szu.edu.cn

ZPL: sea@njau.edu.cn

SQ: sqin@yic.ac.cn

Additional file 1 - Table S1 The details about the completeness of genome sequences used in this study.

The details about the completeness of genome sequences used in this study were summarized from (DOE Joint Genome Institute, project list: http://genome.jgi.doe.gov/genome-projects/). The genomes of the red alga *Cyanidioschyzon merolae* was obtained from the *C. merolae* Genome Project (http://merolae.biol.s.u–tokyo.ac.jp).

**Species**	**Product name**	**Status**	**Last updated**	**JGI project ID**
Chlamydomonas reinhardtii	Standard Draft	Post-Draft-Seq	28-Aug-2008	16938 (Assembly v4.0)
Chlorella sp. NC64A	Standard Draft	Complete	17-Oct-2007	16663 (Assembly v1.0)
Coccomyxa sp. C-169	Standard Draft	Complete	01-Apr-2009	16665 (Assembly v2.0)
Volvox carteri	Standard Draft	Complete	26-Jun-2008	201861 (Assembly v2.0)
Chlorella vulgaris	Standard Draft	Complete	01-Jun-2007	16662 (Assembly v1.0)
Micromonas pusilla	Standard Draft	Complete	15-Jun-2011	16152 (Assembly v3.0)
Micromonas sp. RCC299	Standard Draft	Complete	20-Nov-2007	16153 (Assembly v3.0)
Ostreococcus sp. RCC809	Standard Draft	Complete	08-May-2008	16233 (Assembly v2.0)
Ostreococcus tauri	Standard Draft	Complete	19-Dec-2011	16178 (Assembly v2.0)
Ostreococcus lucimarinus	Standard Draft	Complete	12-Nov-2011	400391 (Assembly v3.0)
Phaeodactylum tricornutum	Standard Draft	Complete	12-Nov-2008	16244 (Assembly v2.0)
Thalassiosira pseudonana	Standard Draft	Complete	09-Dec-2008	16452 (Assembly v3.0)
Fragilariopsis cylindrus	Standard Draft	Complete	20-Nov-2008	16035 (Assembly v1.0)
Aureococcus anophagefferens	Standard Draft	Complete	02-Apr-2009	16554 (Assembly v1.0)
Emiliania huxleyi	Standard Draft	Complete	28-Jan-2008	16965 (Assembly v1.0)
Guillardia theta	Standard Draft	Complete	28-Sep-2010	16067 (Assembly v1.0)
Bigelowiella natans	Standard Draft	Complete	05-Apr-2010	16592 (Assembly v1.0)
Cyanidioschyzon merolae	Standard Draft	Incomplete	17-Sep-2005	
