# Supplementary material for: Evolutionary origins, molecular cloning and expression of carotenoid hydroxylases in eukaryotic photosynthetic algae
Source: BMC Genomics. 2013 Jul 8;14:457. doi: 10.1186/1471-2164-14-457 (PMC3728230; doi:10.1186/1471-2164-14-457)
Supplement: Additional file 2 — The deduced protein sequences of genes encoding CHYs from 18 algal genomes. The red indicated genes encoding BCH-type CHYs in algal genomes. The blue indicated two genes encoding proteins which belong to no one subfamily of CYP97. [file 1471-2164-14-457-S2.pdf]

Evolutionary origins, molecular cloning and expression of carotenoid hydroxylases in eukaryotic photosynthetic algae

Hongli Cui[1, 2§], Xiaona Yu[3§], Yan Wang[2], Yulin Cui[2], Xueqin Li[4], Zhaopu Liu[3] and Song Qin[1*]

[1]Key Laboratory of Coastal Biology and Biological Resources Utilization, Yantai Institute of Coastal Zone Research, Chinese Academy of Sciences, Yantai 264003, People's Republic of China

[2]University of the Chinese Academy of Sciences, Beijing 100049, People's Republic of China

[3]College of Resources and Environmental Sciences, Key Laboratory of Marine Biology, Nanjing Agricultural University, Nanjing 210095, People's Republic of China

[4]Shenzhen Key Laboratory for Marine Bio-resource and Eco-environment, College of Life Sciences, Shenzhen University, Shenzhen 518060, People's Republic of China

[§]These authors contributed equally to this work.

[*]Corresponding author

E-mail addresses:

HLC: hlcui@yic.ac.cn

XNY: 2011103006@njau.edu.cn

YW: ywang@yic.ac.cn

YLC: yulincui@yic.ac.cn

XQL: 2110180316@email.szu.edu.cn

ZPL: sea@njau.edu.cn

SQ: sqin@yic.ac.cn

Additional file 2 - The deduced protein sequences of genes encoding CHYs from 18 algal genomes.

The names of each sequence are listed in the next page. The red indicated genes encoding BCH-type CHYs in algal genomes. The blue indicated two genes encoding proteins which belong to no one subfamily of CYP97.

>LUT-1
MMLSNRTSGRPTVGSRSSSSARRPALFVPVKHVSRVAPLRAQNEDDEPSTFGKNIDSKGAGTSFTSPGWLTQL
NMLWGGKSNVPVANAQPDDIKELLGGALFKALYKWMQESGPIYLLPTGPVSSFLVVSDPAAAKHVLRSTDNSQ
RNIYNKGLVAEVSEFLFGKGFAISGGDAWKARRRAVGPSLHKAYLEAMLDRVFGASSLFAADKLRKAAAEGTPV
NMEALFSQLTLDIIGKSVFNYDFNSLTSDSPVIQAVYTALKETEQRATDLLPLWKVRGIGWLIPRQRKALEAVELIR
KTTNDLIKQCKEMVDEEEMRAASAAAAAGTEYLNEADPSVLRFLIAAREEVDSTQLRDDLLSMLVAGHETTGE
GGRCPLYLPDDEPHPSCSMQAEVDAVLGSRLSPTMADYGQLRYVMRCVNESMRLYPHPPVLLRRALVEDELP
GGFKVPVGQDVMISVYNIHHSPAVWDDPEAFIPERFGPLDGPVPNEQNTDFRYIPFSGGPRKCVGDQFALME
AVVALTVLLRQYDFQMVPNQQIGMTTGATIHTTNGLYMYVKERGAAASGSSGVAGGKQLAAA
>LUT-2
MQTQRPLASPGRQASIPARRAYSLRPPLTQRQLPIARAEPPQTEEIKLFGIIPTAPRSSKLGENLEDRIQSGEFTDS
GSTKEKLTRPLRQALAKEPLVGRSAARFLADLGRQWRAEASKRMPEARGDIREIVGQPVFVPLYKLFLVYGKIFRL
SFGPKSFVIISDPAYAKQILLTNADKYSKGLLSEILDFVMGTGLIPADGEIWKARRRAVVPALHRKYVMSMVDMF
GDCAAHGASATLDKYAASGTSLDMENFFSRLGLDIIGKAVFNYDFDSLAHDDPVIQAVYTLLREAEHRSTAPIAY
WNIPGIQFVVPRQKRCQEALVLVNECLDGLIDKCKKLVEEEDAVFGEEFLSERDPSILHFLLASGDEISSKQLRDDL
MTMLIAGHETTAAVLTWTLYLLSQHPEAAAAIRKEVDELLGDRKPGVEDLRALKMTTRVINEAMRLYPQPPVLI
RRALQDDHFDQFTVPAGSDLFISVWNLHRSPKLWDEPDKFKPERFGPLDSPIPNEVTENFAYLPFGGGRRKCIG
DQFALFEAVVALAMLMRRYEFNLDESKGTVGMTTGATIHTTNGLNMFVRRRDPLTVPPTSSSVAETVSTGYAF
ACGPAVMPVASAEVVAAPATAAGGGCPFHTAAGAAVPAATMSLRPTGPPSA
>LUT-3
MSPALFNPYVAPNRIAPGPRCRMLQRGAAGRGTARGVAATTHAARPYVPRWSRAAAARVVRAAAPSPPPAQ
DTARGTEAAGEAVFQSSSKKKRRNAVKPPLSRGPLLSLSALFGRGGGQQQQGPTPEVRVPLNNVGKVPIFQLLY
ELYSSHGGVFRMRLGPKSFLVLSDPGAVRQVLVGAVDKYSKGILAEILEFVMGNGLLAADGEHWIARRRVVAPA
LQRKFVSSQVALFGAATAHGLPQLEAAAAAAAAAAGDSRGGGAASVDMESFFSRLSLDIIGKSVFDYDFDSLRH
DDPVIQAVYSVLRESTVRSTAPFPYWKLPGISLLVPRLRESDAALAIVNDTLDRLIARCKSMVGRCCGGGGGGG
GGSSAPTVLHFLLGSGEALNSRQLRDDLMTLLIAGHETTAAALTWALHLLVAHPEVMKRVRDEVDWVLGDRLP
GSDDLPLLRYTTRVVNEALRLYPQPPVLIRRAMQDDVLPGGHVVAAGTDLFISVWNLHHSPQLWERPEAFDP
DRFGPLDSPPPTEFSTDFRFLPFGGGRRKCVGDMFAIAECVVALAVVLRRYDFAPDTSFGPVGFKSGATINTSNG
LHMLISRRDLTGVPPPAPRAPAAAAGAAAGSCPHAAAAAATAAAAAAVGCPHAAAAATSGAPAGVTPQ
>LUT-4
MQCQLHQQRRAASSASAGRPAGRPSLPLRPLVRASATSSSAPASSSSPLLARRVGSGVTASPLRPGNPSGRNAG
LRSRVVPQGLPDPVAVGLFFAPGLAILAYAMVRGKGNLTDGLSRVLTESEFLSLGLDIIGLGVFNFDFGSINSESPVI
KAVYGVLKEAEHRSTFYLPYWNLPLADVLVPRQAKFRADLKVINECLDNLIKQARDTRVAEDAEALQNRDYSKV
SDPSLLRFLVDMRGEEPTNKQLRDDLMTMLIAGHETTAAVLTWAMFCLVQSPESEAKVLEEVDRVCGDRTPTL
EDLKAMPYLRACLGESLRMYPQPPILIRRALAEDTLPAGLRGDPAGYPIGKGADLFISVWNLHRSPYLWKDPDTF
RPERFFEPNSNPDFGGKWAGYRPDAVTGGAALYPNEVASDFAFIPFGGGARKCVGDQFAMFEATVAAAMLLR
RFTFRLAVPAEKVGMATGATIHTANGLSMRVTRRTPSGGSGSGAPGAAAKVPATV
>LUT-5
MMLASRPAVALGARAQPQVLRPTLVPRPGMVSNLRLQPVKVADPIVASETSQVMEAPQEKKLSEFELKRLERK
QQRAQEAATYKFSAIAATVLVLSIAVVATYYRFAWHFAEDGDLPVDEMAATLLLVFGGMFGMEMYARFAHKVL
WHDFEPGWALHKSHHEPRTGPFELNDIYAVANALPAMALCAYGFFTPHVIGGVCFGAGLGITLFGIAYMFFHD
GLVHRRFPVGPIANLPYMKRIMVAHQIHHTNKFGGVPFGMFLGVQELEAVPGGKEELDKLMADLEAREAAAA
KAAGSS
>LUT-6

MLLSGRVAQPKACGHASNNPRRRPVPFQSYHQANRIVKVRAQDDEPIMGKSIDAAGAGASFTSPGWLTQLN
MLWGGKGNVPVANAQPDDIKELLGGALFKALYKWMQESGPVYLLPTGPVSSFLVISDPAAAKHVLRATDNSQ
RNIYNKGLVAEVSQFLFGKGFAVAGGDDWKVRRRAVGPSLHRAYLEAMLVRVFGPSSEFAADKLRVAARSGTP
VNMEAMFSQLTLDIIGKAVFNYDFNSLTSDSPLIQAVYTALKETEQRATDLLPLWKVPALGWLIPRQRKALQVRH
STLVRRPVKKNLFSRSVCVCVCFAFVVLLCAAANGDFTAKQQHQCRVVPFLNQPSNPPPSRRCVSLFPSPLPAAS
LARGSALTWTLYLLVQNPDKMAKAVAEVESVMGSRTAPTLADYGQLRYVMRCVNESMRLYPHPPVLLRRALVE
DELPGGYKVPVGQDVMISVYNIHHSEAVWDNPEAFIPERFGPLDGPVPSEQNTDFRYIPFSGGPRKCVGDQFA
LMEAVVALAVLLRQFDFSLVPNQKIGMTTGATIHTTDGLYMYVKERRTCAGQAAAGAAAVTAG
>LUT-7
MQQHQSRTLGGRPQQQPQRLPRCPVLRSAGISRSRPIVHAEPTEGNQPDSGKLFGLIPLRARGENLDARIESG
EFTDAGSTKEKLTRPLRQALAKEPIVGRPVARFLADLGRRWRSEAAKRMPEARGDIREIVGQPVFVPLYKLFLVY
GKIFRLSFGPKSFVIISDPAYAKQILLTNADKYSKGLLSEILDFVMGTGLIPADGEVWKARRRAVVPALHRKYVASM
VGMFGDCTVHGTATLDCAVASGQSIDMENYFSRLALDIIGKAVFNYDFDSLTHDDPVIQAVYTVLREAEHRSTA
PLAYWNLPGATIVVPRQRRCQEALRIVNDTLDGLIDKCKKLVEEEDMEFNEEFLSDQDPSILHFLLASGDEISSKQ
LRDDLMTMLIAGHETTAAVLTWTLYTLASHPEATEAIRREVDEVLGDRAPNVEDFKSLRFTTRVINEAMRLYPQP
PVLIRRALQEDKFDQYVVPAGSDLFISVWNLHRSPELWDEPDKFKPERFGPLDGPIPNEVTENFGYLPFGGGRR
KCIGDQFALFEALVALAMLVRRYDFVLDTSKPPVGMTTGATIHTTGGLYMHVKKRDMSGLAAAVRRQETPAYAF
AYGTSTVAAMASPASSSPAAVAGGGCPFHTGAAVPPPPPAAVAAASVTVGGATATLGGGVSIGPSAPAGPAGL
>LUT-8
MQQLNCRSGARGPLRIAAGCSPRPRRAHAFSPNPFNPSLSSLATSPLRPGRLSRSTGLQSRLIPAGLPDPLAVGL
FFAPGFAALVYAYFRGKGNLTDGLSRLLTEISQGYFQPDVGGKNIPVAQGELSDLAGDQPLFKALYQWFIESGGV
YKLVFGPKAFIVVSDPVVVRHLLKDNAFNYDKGVLAEILEPIMGRGLIPADLDTWRVRRRAVVPAFHRQYYDAM
VTMFGRCADRSSDKLQALVEKGQVGLGGRVVDMESEFLSLGLDIIGLGVFNYDFGSITSESPVIKAVYGVLKEAE
HRSTFYLPYWNLPLADVLVPRQAKFRRDLRVINDCLDDLIRKAQETRVEEDAEALQNRDYSKLRDPSLLRFLVD
MRGEDVTNKQLRDDLMTMLIAGHETTAAVLTWALYCLMQSPAALERVLREVDGVERGGNPQGETVADLEAC
KGDPLGESLRMYPQPPILIRRALGEDVLPGGLRGDPAGYPIGTGADLFISVWNLHRSPYLWKDPDTFRPDRFFES
YSNPDFEGKWAGAYAVSGGAALYPNEVGSDFAFIPFGGGARKCVGDQFAMFEATVALAVLLRRFSFALEGPPEK
VGMATGATIHTANGLMVRVSRRTPPPPPPAPAAGSPREEQLPRQPVAA
>LUT-9
MLLSARPMRVRLALASGHAKPIRPTLWPRLQVLSHQRLQPAGVADSASDGLSTHQVNLMLRQPQLVEPSDFQ
KLRLERKRQRTKEAMAYRFSAIAATVLVLSIAVVATYYRFHWHLAEEGDVPLDEVAATLLLVFGGIFGMEMYARY
AHKVLWHDFEPGWALHKSHHEPRTGPFEANDIYAVVNAVPAMALCLYGFLTPHVTGGVCFGAGLGITLFGIMY
MFFHDGLVHRRFPVGPIAEVPYMKRIMVAHQIHHSNKFGGVPFGMFLGPQELEAVPGGKEELDRLMAALEA
REA
>LUT-10
LSSYENELLAAWDTDSSLQRGFDWEIEKLRRNFAGLRQREDGQWVRKPSLFDFLVTNTPSNVVGVSNTGERYE
SPPKPVNMLDVGLLITKNLLNTLGFGPSLGMAAVPDAVIQKYEGSFFSFIKGVLGGDLQTLAGGPLFLLLAKYYQ
DYGPIFNLSFGPKSFLVISDPVMARHILRDSSPEQYCKGMLAEILEPIMGDGLIPADPKIWKVRRRAVVPGFHKK
WLNNMVTLFGDCGERLVNDLDARATAKTPVDMEERFCSVTLDIIGKAVFNYDFGSVTKESPIVKAVYRVLREAE
HRSSSFIPYWDLPYADKWMGGQVEFRKDMGMLDDILTKLINRAIETRDEASVEELEDRDVGDDPSLLRFLADM
RGEDLTSKVLRDDLMTMLIAGHETTAAMLTWTVFGLVSNDSGLMKEIQAEVRTVMGDKLRPDYDDIAKMKK
MRYALIEALRLYPEPPVLIRRARSEDNLPAGGSGLSGGVKVLRGTDIFISTWNLHRAPEYWENPEKYDPTRWER
RFKNPGVKGWNGYDPEKQSESSLYPNEITADYAFLPFGAGKRKCIGDQFAMLEASVTLAMIINKFDFTLVGSPKD
VGMKTGATIHTMNGLNLVVSRRSEDNPIPETNDYWIQQHLSRGLNVNGRPYSTNEDAAWTASSRDKNEGVV

SRLVN
>LUT-11
MQPAEPGTDIIFADSARVLRTNIEQIYGGFPSLDQCPLAEGEITDIADGTMFIGLQRYQQQYGSPYKLCFGPKSFL
VISDPVQAKHVLRDANTLYDKGILAEILKPIMGKGLIPADPETWSVRRRAIVPAFHKAWLNHMVGLFGYCNEGLI
ASLEEAAKKNDAPNGQQGGKIEMEEKFCSVALDIIGLSVFNYEFGSVSEESPVIKAVYSALVEAEHRSMTPAPYW
DLPFANEVVPRLRKFNSDLKVLDDVLTDLIDRAKNSRQVEDIEELEKRDYANVKDPSLLRFLVDMRGADIDNKQL
RDDLMTMLIAGHETTAAVLTWALFELTKHPEQMAKVRAEIDSVLGDRTPTYDDIKEMQYLRLVVAETLRLYPEP
PLLIRRCRTENKLPKGGGREATVIRGMDIFLSLYNLHHDERFWPEPNEFKPERWESKYINPEVPEWAGYDPAKW
INTNLYPNEVASDFAYLPFGGGARKCVGDEFATLEATVTLAMLLRRFEFEFDSAKLAASKIDIMDHPEDLEHAVG
MRTGATIHTRKGLHMVIRKREL
>LUT-12
MRSSDYSRAPRWESFVVLAWATASAVLVGNVFQQALPQPVQAFGPALVPHLSRRCDLIQRRVSSDLEDVDVDV
DNDAILGSNNIPRRLRIQGRGIPTRKDPIQPLDSMTYESDLIKTWEQDPSRQKGFDWEIEKLRRYFAGLRMRDD
GVWVRQPSFFDFLVSKSRSDPGNAPRPVGLVDVVKLVLTNSLTSVGLGPALGMAAVPNAVIQKYEGSFFSFIKGV
LGGDLQTLAGGPLFLLLNKYFEVYGPIFNLSFGPKSFLVVSDPVMARHVLRETSPDQYCKGMLAEILDPIMGKGLI
PADPATWKVRRRAIVPSFHKRWLNRMITLFAERAEILADDLQPKSAKGQVVDMEERFCSVTLDIIGKAVFNYDF
GSVTDESPIIKAVYRVLREAEHRSSSFIPYWNLPYADQWMGGQVEFRKDMTMLDDILADLINKAVSTRREASIE
ELEKRENEDDPSLLRFLVGMRGEDLSSMVLRDDLMTMLIAGHETTAAMLTWTLFELSRGDPGLLKEVQAEVRT
VLKGKERPDYDDIVAMKKLRYSLIEALRLYPEPPLLIRRARTEDNLPAGSSDLKSGVKVLRGTDMFISTWNLHRSP
DLWENPEVFDPTRWDRPFNNAGIPGWSGYNPDKVSGLYPSENAADFAFLPFGGGQRKCVGDQFAMMEATV
TMALMIKKYDFDFAIPAEDVGMKTGATIHTMNGLMMRARQVNENEPVQSAEGYWEMQHLKRGLNANGRP
YTTEEEAVWQTSERLSHKKEEEKPNGEGGCPMHKM
>LUT-13
MQVGKSGEEISFGDSANVLRTNIEQIYGGFPSLDGCPLAEGELADIGDGTMFIGLQNYYRNYGSPYKLCFGPKSF
LVISDPVQAKHILKDANTNYDKGVLAEILEPIMGKGLIPADPETWSIRRRQIVPAFHKAWLEHIVGLFGYCNQPLI
DTLNKRVDGDGKVEMESLFCSVALDIIGLSVFNYEFGSVTQESPVIKAVYSALVEAEHRSMTPAPYWNLPLANQL
VPRLRKFNSDLKLLNDVLDDLITRAKQTRTVEDIEELENRNYNEVQDPSLLRFLVDMRGADIDNKQLRDDLMT
MLIAGHETTAAVLTWALFELTKNPEIMKELQDEIDEVVGDRMPNYEDIKKMKFLRLVVAETLRMYPEPPLLIRRC
RTPDELPQGAGREAKVIRGMDIFMAVYNIHRDERFWPSPDTFDPLRFTRSHSNPDVPGWAGFDPKKWEGKLY
PNEVASDFAFLPFGGGARKCVGDEFAILEATVTLAMVLRRFEFSFDESKFEGKDDILSSAQGLNHPVGMRTGATI
HTRNGLHLVVEKRGVPK
>LUT-14
MAAPDWMTQLNRLWGGASEIPVADAKLEDITGLLGGGLFQPLFKWMRESGPVYLLPTGPITSYVVVSDPDCIK
QVLFNYGSRYIKGTIAEAGEFLFGLGVALQELEPWKIRRKAVAPSLHRKYVEAMVDRCFGPCADRMVSILEGEA
GAGGVGGVNMESRFSKTALDIIGISVFNYDFEALTTAAPVIQATYTALKEVETRSMDLLPTWRLPEKFLRVVSPR
QRDAQDAVTVIRDVTQRLVDDCKRMVEEEEKVGGAEEWARDYLNESNPSVLRYLIAAREEVSSTQLRDDLLSLL
VAGHETTASVLTWGTYELLKPENAEQLRLLRAELDEVLGTRPFPTFADLPKMPYLERCFHESMRLYPQPPVYTRR
AVVEDVLPNGMTIPKNQDLLVSIYNLHRSPTSWGPTSQEFEPMRFGPLANGQPNELNTDYRYVPFSAGPRRCP
GDKFAVYEGIVIWATMFRRLDLELKAGHDVVMTSGATIHTKSGLLATVKARAMREVAEADRVDWANLKPAKDI
GEEWMEKALFNSEATGAVSAGKCPMGH
>LUT-15
MPVASGDIREIAGQPVFVPLYKLFLAYGEMFVLAIGPKKFVVVSDNAVAKEMLLTQAKSFSKGLLSEILDFVMGQ
GLIPANGEVWKIRRKVIVPSLHKKYVTSMVDMFGDCGLKGMSQLARAEKANESVEMENFYSRFALDIIGKAVF
NYDFDSLSTDDPVIKAVYTVLREAEYRSVTFIPYWKVPPLRWLVPRQRQCQEALQVVNDTLDDLINRCKAVVEE

EDEEFVEEYMNTDDPSILHFLIASGDDVTSKQLRDDLMTLLIAGHETTAAVLTWTTFLLAKHPEVKAKVFEEVDR
VVGDRNPTVADMRALVYTTRVINESMRLYPQPPVLIRRALEPVTLGGYNIDAGTDFFISVWNLHRNPRIWDEP
DAFKPERFPIDAPMPNEYTEEYAYLPFGGGQRKCVGDQFAIFESIVSLAMLMRRFDFELDESKHPDGECGMTT
GATIHTTNGLHVKLKRRDGRGGREMDGTYVTGMALSNLEDVDVVRGSIDAPT
>LUT-16
KMPVASGDIREIAGQPVFVPLYKLFLAYGEMFVLAIGPKKFVVVSDNAVAKEMLLTQAKSFSKGLLSEILDFVMG
QGLIPANGEVWKIRRKVIVPSLHKKYVTSMVDMFGDCGLKGMSQLARAEKANESVEMENFYSRFALDIIGKAV
FNYDFDSLSTDDPVIKAVYTVLREAEYRSVTFIPYWKVPPLRWLVPRQRQCQEALQVVNDTLDDLINRCKAVVE
EEDEEFVEEYMNTDDPSILHFLIASGDDVTSKQLRDDLMTLLIAGHETTAAVLTWTTFLLAKHPEVKAKVFEEVD
RVVGDRNPTVADMRALVYTTRVINESMRLYPQPPVLIRRALEPVTLGGYNIDAGTDFFISVWNLHRNPRIWDE
PDAFKPERFPIDAPMPNEYTEEYAYLPFGGGQRKCVGDQFAIFESIVSLAMLMRRFDFELDESKHPDGECGMTT
GATIHTTNGLHVKLKRR
>LUT-17
MRRATPRRGDAARERAVGDAWNRAVVARAVKEPEEETIPSDDFKPEELKFRDIVSLWVTQILQTYGDKESKDN
APVCEGVIDDLVGGPIFLALYPYFRRYGGVFKLAFGPKVFMVLSDPVVVREVLKEKPFSFDKGVLAEILEPIMGQG
LIPAPYAVWKNRRRQLVPGFHKAWLDHMVGLFGHCSNALVRNLDKAAASGEVVDMEERFCSVSLDIIGLAVF
NYDFGSVTKESPIISAVYNCLQEAAHRSTFYFPYWNLPFATDIVPRQREFKKNMSIINDTLNGLIKQAQQFEGTD
DLEELQNRDYSKVKDPSLLRFLVDIRGADVTDVQLRDDLMTMLIAGHETTAAVLTWGLFCLVQKPELLKRIQADI
DEVFGDDDRTPTYDDIQKLESVRLCIAEALRLYPEPPILIRRCLEDVTLPKGAGDVEVTLIKGMDIFISVWNLHRSP
ECWENPDEFDPFRFKRPFKNPGVKDWAGYNPDLLTGLYPNEVASDFAFIPFGAGARKCIGDQFAMLEATIAMA
MTLRRYDFELQKDPKDIGMEMGATIHTAGGLPMKIKRRTPAA
>LUT-18
MRATPSTLRTPSRARAPGAANSRGSTARARAIQPPKAPDAYRGALFPGVEVPDNDLARSFSALFPWGNGARVT
EKVLGDLLKPEVRAAPLFVPLYDYYREYGGVYNLGAGPKWFVVVSDPVAVRTMFKDQADSFSKGILTDIMEPIM
GDGLIPANKEIWAKRRPVIGAGFHGAWLKHMCNLFGASAMRLADKLDTFVESEKTVELESELYAMALDVIGKA
VFNYEFGALKQETPIIKAVYRVLRESEHRSTFPLQYWQIPGAMELVPRQKQFKEDMKMVNDELSVLINNAIASR
NETGLEEMERRDYSNVEDASLLRFLVDIRGDEATSTQLRDDLMTMLIAGHETTAAVLTWTLYLLAQHPEIADDA
VAEINACVENADGIPTPEEVRKLEKVRMILAEGMRLYPAPPILIRRAIKDVTLPRGGNGKEITLKAGTDCFIAVWN
LHRSPDLWEDPEKFDPSRFSRRFENPAIEGWGGLNPELMTGLYPNEQCTDFSYVPFGGGQRRCAGDQFAMLE
AVTALSVLLKKFKFELACEPGEVEMITGATIHTKKGLPMKLKRR
>LUT-19
ARYAHKHLWHASWWSMSSKYRREWNKPIWLLHESHHLPREGAYEANDVFALVNGVPAFALCAFGFFTPGVF
GGLCFGAGLGITLYGIAYMYVHDGLVHKRFPTGPLGKLPLLRKIAAGHTIHHTEAFEGVPWGLFLGIQELEAVPG
GLDELNK
>LUT-20
APDWMTQLNRLWGGASEIPVADAKLEDITGLLGGGLFQPLFKWMLEAGPVYLLPTGPVTSYVVVSDAACIKQV
LFNYGSKYIKGTIAEAGEFLFGLGVALQELEPWKVRRKAVAPSLHRKYVEAMVDRCFALCADRMTTILEEEAAN
GAVGSVNLESRFSKTALDIIGISVFNYDFKALTTAAPVIQATYTALKEVETRSMDLLPTWRLPEQFLRIVSPRQRNA
QDAVTVIRDVTQRLVDDCKRMVEEEEKVGGAEEWARDYLNESNPSVLRYLIAAREEVSSTQLRDDLLSLLVAGH
ETTASVLTWGTYELLKPENAEQLRLLRAELDEVLGTRPYPTFADLAKMPYLERCFHESMRLYPQPPVYTRRAVVE
DVLPNGMTVPKNQDLLVSIYNLHRSPANWGPTSQQFEPMRFGPLANGQPNELNTDYRYVPFSAGPRRCPGD
KFAVYEXIVIWATMLRRLDLELKAGHDVIMTSGATIHTKSGLLATV
>LUT-21
MSKMPVATGDIREIAGQPVFVPLYKLFLAYGEMFVLAIGPKKFVVVSDNAVAKEMLLTQAKSFSKGLLSEILDFV

MGQGLIPANGEVWKIRRKVIVPSLHKKYVTSMVGMFGDCGLKGMAQLARAEKMGESVEMENFYSRFALDII
GKAVFNYDFDSLTTDDPVIKAVYTVLREAEYRSVTFIPYWKVPPLRWLVPRQRQCQEALKVVNDTLDELIDRCKK
IVEEEDEEFVEEYMNTDDPSILHFLIASGDDVTSKQLRDDLMTLLIAGHETTAAVLTWTTFLLAKHPDIKQKVFEE
VDRVVGDRNPTVADMRELVYTTRVINESMRLYPQPPVLIRRALEPVTLGGYNIDAGTDFFISVWNLHRNPRIW
PEPDAFKPERFPIEGPMPNEYTEDYAYLPFGGGQRKCVGDQFAIFESIVSLAMLMRRFDFELDESKHPDGECGM
TTGATIHTTNGLHVRLKRREGRGGAEMSGAVRVYSSSLR
>LUT-22
MVARARVHASRGVDARRVRARGRARVDVIARAVKEPSSAPEEALPDENFKPEQLKFQDIVSLWVTQILQTYGG
KESKDNAPVCEGVIDDLVGGPIFLALYPYFRRYGGVFKLAFGPKVFMVLSDPVIVREVLKEKPFSFDKGVLAEILEP
IMGQGLIPAPYAVWKNRRRQLVPGFHKAWLDHMVGLFGHCSNELVRNLDKSAEDGEVVDMEERFCSVSLDII
GLAVFNYDFGSVTKESPIISAVYNCLQEAAHRSTFYFPYWNIPFATDIVPRQREFKQNMKIINETLNGLIQKAQKF
EGTEDLEELQNRDYSKVKDPSLLRFLVDIRGADVTDSQLRDDLMTMLIAGHETTAAVLTWGLFCLMQNPELMK
RIQADIDEVMGDDDRTPTYDDIQKLESVRLCIAEALRLYPEPPILIRRCLEDVTLPKGAGDAEVTLIKGMDIFISVW
NLHRSPECWENPEEFDPFRFKRPFANPGVKDWAGYNPELFTGLYPNEVASDFAFIPFGAGARKCIGDQFAMLE
ATIAMAMVLRRYDFELTTDPKDIGMTMGATIHTEKGLPCRVRRRQPVTTATAAAV
>LUT-23
MDLLKPEVRAAPLFVPLYDYYREYGGVYNLGAGPKWFVVVSDPVAVRTMFKDKADDFSKGILTDIMEPIMGDG
LIPANKETWAKRRPVIGAGFHGAWLKHMCNLFGASAMRLAEKLDVAAEKGTTVELEGELYAMALDVIGKAVF
NYEFGALKQETPIIKAVYRVLRESEHRSTFPLQYWQIPGAMDLVPRQKQFKEDMKMINEELSTLINSAIESRNET
GLEEMERRDYSNVEDASLLRFLVDIRGDEATSTQLRDDLMTMLIAGHETTAAVLTWTLYLLAQHPEIMEEAVKEI
EMCVENADGVPTPEEVRKLEKVRMILAEGMRLYPAPPILIRRAIKDVTLPRGGNGKEITLKAGTDCFIAVWNLH
RSPDLWENPEKFDPSRFSRRFTNEAIEGWGGLNPDLMTGLYPNEQSTDFSYVPFGGGQRRCAGDQFAMLEAV
TSLSVLLKKFKFELDCPPNDVEMITGATIHTKKGLPMKIKRRE
>LUT-24
IFQRHCVVVADPELVKRVMQTNLKNYKKDTEFSYEPFLEILGTGLVTSEGETWRAQRQRISSALRIEILDDIIAIATR
ALEKVRGKGEAVELAEEFRLLTLQVIAEAILSLTPEQSDEVMPNLYLPIMDECNRRSLEPWRKFLPTREWREHRKR
VAALNKYIVDLIRVRWKKRVSGETNPNPDILDRVLASVEMEEYGSDVEEQMCFEIKTFLLAGHETSAAMLVWTI
YELVKNEDKMTEAVAEANKVLGAVKPGNLPTRDELAHLDYCVSALKETLRLYSVVPVVTRRAVEDDVLGGCKIPK
GTTVIISLQGIHHREDLWPNAMSFEPERFLNGKGDEIGNYAYLPFIQGPRNCLGQYLALLEARVVLATLIRRFKFK
SASANNGKKHTKAIPIAPADGMWFTVE
>LUT-25
RRKARTKAEREASAASYEWSAWASSCGVISVAITATYFRILREVDVNGGVFPVAELVAQLALIAGAAVGMEFYAR
YAHKHLWHGSXPIWLLHESHHLPREGAFEANDVFALMNGVPAFALCAFGFFTPGVFGGLCFGAGLGITLFGIAY
MYVHDGLVHKRFPTGPLGKLPVMRRIAAGHTIHHTEAFEGVPWGLFLGIQELAAVPGGLEELEKVVI
>LUT-26
MAAPDWMTQLNRLWGGKSEIPVADAKLDDITGLLGGGLFQPLFKWMKEAGPVYLLPTGPITSYVVVSDPDCI
KQVLFNYGSKYIKGTIAEAGAFLFGLGVALQENEAWKIRRKAVAPSLHRRYVEAMVDRCFGPCADRMVSLVED
QINADGGRRERVNMESKFSQAALDIIGISVFNYDFKALTSAAPVIQATYTALKEVETRSMDLLPTWRLPEQFLRIV
SPRQKAAQDAVTVIQEVTTKLVDDCKKMVEEEEAVGGAEAWARDYLNDANPSVLRYLIAAREEVSSTQLRDDL
LSLLVAGHETTASVLTWGTFELLKPENAEQLRLLRAELDEVLGDKPFPDYADMLKLPYLERCFHESMRLYPQPPV
YTRRAVVEDVLPHGLGTIPAGQDLLVSIYNLHRSPANWGPASQAFEPMRFGPLSAGQPNELNTGYRYTPFSAGP
RRCPGDKFAVLEGMAIWAVLFRRLDMELVAGHDVVMTSGATIHTRDGMLVNATRRETRRKGGGDAVDWAN
LRPAKDIGEGWWERGIETKGGSGADAKSKCPMPFVK
>LUT-27

MPVAAGDIREIAGQPVFVPLYKLFLAYGEMFVLAIGPKKFVVVSDNDVAREMLKDQATSFSKGLLSEILEFVMGT
GLIPADGETWKVRRRTVVPSLHKKYVASMVDMFGDCGLNGSAQLARSEMNGDTVEMENFYSRLALDIIGKAV
FNYDFNSLKMDDPVIKAVYTVLREAEYRSVTFIPYWKVPPLRWLVPRQKACQEALVVVNDTLNMLIARTKKLVE
EEDEEFVEEYLNKADPSILHFLIASGDDVTSKQLRDDLMTLLIAGHETTAAVLTWTTYLLATHPEIKARVQAEVDE
VCGDRNPTIADMMDLKFTTRVINESMRLYPQPPVLIRRALEPVTLDGYKIDAGTDFFISVWNLHRNPRLWENP
DKFDPDRFPIDQKMPNEITENFAYLPFGGGQRKCVGDQFALFESIITLAMVCRRFDFELDAKFHPDGECGMTT
GATIHTTGGLHVKLKRRDGAGGNEMLGSIDCGDGVKCASLGELSDVNTGDGTPESGAASFDEAERAQARDLK
EAAVVLGAKTAGASELKSGSGGAKGEKPSIDGAALEEAVKEAEALYEAEAEREAAMKKELEQSL
>LUT-28
MERPSQTYGDEESKDGAPVCEGSVDDLVGGPIFLALYPYFLKYGGVFKLAFGPKVFMVLSDPVIVRRVLKEKPFA
FSKGVLAEILEPIMGQGLIPAPYAVWKNRRRQLVPGFHKAWLDHMVGLFGDCSAQLVKNLGASHLTLTDASIAA
GNGVARIDMEERFCSVSLDIIGLAVFNYDFGSTTRESPIIKAVYTCLQEAAHRSTFYFPYWNIPFMCDIVPRQREF
KANMKLINDTLNGLITQAQQFEGTEDLEELQNRDYSKVKDPSLLRFLVDIRGADVTDLQLRDDLMTMLIAGHET
TAAVLTWCLFCLVRDKPLMKKVVEEIDSVMGPVAEEARAPNYEEIQKLELVRLCLAEALRLYPEPPILIRRCLEDVP
LPKGAGDADVTLIKGMDVFISVWNLHRHPDCWEEPLKFDPFRFKKPYSNPGVKDWAGYNPDLISGMYPNEVT
SDFAFVPFGAGARKCIGDQARSCLHWSPYDRFAMLEATSCLAMTLQRYDFELDKDAAEVGMEMGATIHTAGG
LPMRVTRRK
>LUT-29
MAVAPPSEPVLIATSVALAVLTVAFYVSRAVAGFLYGVARWHRQAFVLRHTPTAPGYVPLIGHTIALFRAVGNYP
CTWDLFAMWATATAPKPARVQIFDRHCVVIADPSTMKRVMATNLKNYQKDLEFSYAPFLEILGTGLVTSGGET
WRKMRGHISKALRVEILDDIIAIATRAVERLCVKLDAAKASAAAVDMEQEFRLLTLQVIGEAILSLSPEDSDELFPS
LYLPIMDECNARSLSPWRAWIPTREWFAHKARSIHWFPYDRVGVVNASRVRELDDAIISIVRARWRKKQAGED
VPDDVLERVLEQVREDEYGADVETQLCFEIKTFLLAGHETSAAMLTWTLHELSKAPDMMREVKRESDRVFGRT
RKGSLPTRDQLASMEYTLAAFKETLRLYSVVPVVTRVAVEDDELGGTRVPAGTTVIMSLQGVHHRADLWPEPLK
YDPARFVKADENDEMRDFKFLPFIQGPRNCLGQYLALLEARVVLGTLVRRYAFAPSKAQGKKHTKAIPIAPANG
MHFTVS
>LUT-30
KGEREAQGESYEWAAWVSTCGITSIAITATYLRLLREVTDSGAFPWSELIAQVALIAGAAVGMEFYARFAHKHLW
HDSWWTMPQSWRADWNRPIWLLHESHHLPREGAFEANDIFAVSNGVPAFALCAYGFVTPGVFGGLCFGAGL
GITLFGIAYMYVHDGMVHKRFPTGPLGKNKYLRQIAAGHTIHHTEKFDGVPWGLFLATQELSAVPGGMEELAT
VLEAADRKAAR
>LUT-31
MQAATAVSTAPVAGSRNGAFPAPSKRHAPPRRRLEARAATEEKEEYDLNQKIDTGKLIDAAGAGRSMFSPGWL
TQLNQLWGGKSNVPVANAKPEDIQDLLGGALFKALFKWMVESGPVYLLPTGPISSFLVISDPECAKHVLRASDN
PSRPIYEKGLVAEVSQFLFGEGFAITGGEQWRVRRKAVGPALHRGYLEVMLDRVFGESALHLNKKLEVAAASGE
PIDMEACFSQLTLDVIGKAVFNYDFDALNNNTPVIQAVYTALKETETRATDLLPYWKYPLINLFVPRQRKAAAAV
ELIRQTTEELIAKCKAMVDAEEAASFEEGYINDADPSVLRFLIASREEVGSQQLRDDLLSMLVAGHETTGSVLTW
TLDLLARNPEQMKKAQEEVDRVLAGKSKPNMEDYMALKYCMRCVNESMRLYPHPPVLLRRAMVPDELPGGL
TVPQGGQDVMISVYNIHRSPAVWDRPDDFLPERFPLDGPVPNEQNTDYKYIPFSGGPRKCVGDQFALMEAVVA
LAVMIREFDFAPQPGHDPGMTTGATIHTKNGLYMTVAKRRPGGGSSCSGGAGAAAAAAAVGAA
>LUT-32
MQALAAAPKLAMPGCRAPLPGRALALAPLRHHPQPRRVQRQRPQQQRLVATQAFLSTDTLVGLAIFFSPSVAA
LIYAYIKGKGNLTDGLSRLLTDVSQGYFQPDVGGKNIPVAQGELSDLAGDEPLFKALYKWFIESGGVFKLEFGPKA
FIVISDPLVVRHLLKENYTNYDKGVLAEILEPIMGKGLIPADLETWKVRRRAIVPGFHKAYLDACVAMFGRCTQH

TVDKVEAALAAASPAPDGSQGAAVLDMETEFLNLGLDIIGLGVFNYEFGSITSESPVIEAVYGVLKEAEHRSTFYIP
YWNLPLTKYLVPRQRQFNADLAVKDASLLRFLVDMRDADLEAKQMRDDLMTMLIAGEWRRAAALRLCHETT
AAVCTWTLFCVVQDERVEGKVLAEIDAAVGDRVPTWDDFANLPYTRMTIAEAMRLYPQPPILIRRQAGAGGW
VRVALGEDVLPAGLGGDPNGYPIGKGADLFISLWNLHRSPHLWKDPDTFRPERFTGQLGERFVNAAFGGKWA
GYTPGGEGSSLYPNEVSSDFAFLPFGGGARKCIGDQFAVTEAALILVMLLRRFRFRLQDPQGVGMATGATIHTA
NGLKCTVERRVAAATAAAEPALAN

>LUT-33

EELQQKRVKRAEARRREQLTYQFAAIAASVGVTALAGFATYYRITCYLANDQAFPYLDLACTLALAAGAAFGME
MYARWVHKDMWHDNPVGYILHKSHHEPRTGPFEANDIYAIVNAVPAMALCLYGFLRPDVWGSLCFGAGLGIT
LFGISYMFVHDGLVHRRFPVGPIADLPAMKRIVVAHQLHHSEKYGGVPFGMFLGPQELEAVGAGPELDRMVA
EFDAARAKKAGATAGAGRR

>LUT-34

MQGPKLDAEVFGKDRLKQEEADIRKMEKQGMAKKDVTLTDIISLWITNIILTYGDKEKYAQLQDGAVKSEGVVD
DLVGGPLFLPLYKYFKDCGGLYKLCFGPKVFMVASDPLVIRHIMKDNVFSYDKGVLTEILEPFMGQGLIPAPFQV
WKERRRALVPGFHQAWLNRMCRMFSECTDRLSAKLDAVADTDEIIDMEENWNSCSLDIIGKAVFNYDFGSVE
KLSPVVEAALCALREAEHRSTFYFPYWKIPGLGAEWPIPALVPRQRKFQQDMALLNGVLDKLILNVVNEKQETD
LDALINKDYDNVNDPSLLRFLVDLRGADATQKQLRDDLITLLIAGHETTGSMLTWATWLLSQHPEAQAKMQKEI
DDVLGGRSPTYEDMPRLEQVRLVITETLRLFPEPPILIRRALDADVLPKASNLDGSVQGNAVKIIKGSDFFLSVWN
LHRSPLLWDKPDEFDPDRWRRPTPPELVEKYNAQRRAEGLPEWQGYVPDLKTLYPNEVHADYAFLPFGAGPRK
CLGDQFALMESVVMLTKIFQRYSFELVGNHDPKVPNESDVGMMFGATIHTANGLNVRVKRRQA

>LUT-35

MFMPLNKYFREYGGVYKLSFAPVPQATFYVLSDPHAIKHVLKESPNDFDKGLLSEILEPILGKGLIPADPETWRQR
RPVIQPGFHMRWLERMTMTFNECASIMIDKLEGEADAGNLVDMEGMFNSVSLDIIGKAVFNYEFGSVTRESP
VIKAAYACLKEAEHRSTFLLPYWNVPFLGQGKFSVVPRQREFAAHLEVLNDTLDTIIQKAKSLKNEDDLEALERRD
YKSIQDPSLLRFLVDLRGGDCNDKQLRDDLMTLLVAGHETTGSLLTWTAFELAQNPAEMRKVQEEVDRVLGGR
NPTMDDIKKLEYTRLVLAEGLRLYPQPPILLRRALKETKLPVAHSGSHEDQASSDMQPSGVSISPGANIFISVWN
LHRNPKLWDNPDSFDPSRWLRPQPATNGHSSWAGYTPRKDMGLYPNENDANYGYIPFGGGQRKCVGDQFA
MQEAVVILSKLFQRFDIELAGSPEEVGMSTGATIHSKNGLMIRLKKRNV

>LUT-36

MRHLPEDGEAEHSYKVCLLPKAFLIVSDPTVVRHILSENALKYDKGILADILEPIMGKGLIPADLPTWQPRRRAVV
PGFHSSWLQSMMAVGLFSRCSDRMVGALKESMQRGMGPMSSCWRTQERAGEEVDLESMYSSVALDIIGEA
VFNFKFLSVQRKSPVIDAVYNLMQEAEHRSFFLLPYWKVPVLGFRFLGLGPLVERQQRFEQDIELINDCLDELIKE
ALLTRSEEDIETLQKRDYDALENPSLLRFLVDMRGADATERQLRDDLMTMMIAGHETTAALLTWTTFCLLTNPE
EMKKVHQEIEDVLGGRRATYEDILKMEKTRLALAEALRLYPQPPILIRRALDDDVLPLAWGNEKQVKVFRGTDIF
MLVWNLHRSPVLWGDDADAFRPDRWLSSRSNPDVPGWEGYKPNMKNLYPNEVSSDFAFCPFGAGPRKCIG
DQFAFLESVVILSRVLQEFDIQLATSPEEVGMTTGATIHTEKGLKVSLRARKNVPAA

>LUT-37

MAPRRGVSISCFLLLPCLAASLAASRAGAPAVQRVAAAPPRAASAIAAAPAAAEREKSASNDLIPEEPTLSDWIN
NMPIAMSKSLARQILTSQEKEPEPLPGIWDWFWDSMPFLAAGKKGEPLTLGDVARTFKVNIEQIFGNIPAPDKA
PLAAADVEGLDFKASRYGEMWEIWGGGPAQTGTTAAAAAAIFLALKTYFDRYGSVYKMCFGPKSFMVSSDPVI
ARHVLRENCKNYDKGALALVLEDIMGKGLIPADPVTWAKRRRAIAPGFHKLYLERMVSEFGQANANLIPQLLQA
YGSLALDVIGKAVFNYEFGSVDEESPVVLYKLIDECLDSRNPEELDALKSKDYSKVKDPPSPTPRHETTAAVLTWLT
YALSQHPEALRRVQDEIDTVVGDRYATVDDIKRMPEVQKADQWPEGGTGVEGGFALARANDLFISTYNMGRS
PQLWEEPDVFDPQRWDRPFDNPDVKGWAGYDPAKRTGTGKMKPEGGLWVANLEIASDHAMIPFGAGERKC

VGDQFALLEAAVSVVMLLRRFEFDLEMPDGPVNPAKLDPDNPDKSIGTVGMVSAATIHTATGLFCRVKERFPG
RTDLPPRQDPNLQAPEPEGPALGEPALVP
>LUT-38
MAAPDWLTQLNRLWGGNSEIPVADAKLEDITGLLGGGLFQPLFKWMKEAGPVYLLPTGPVTSYVVVSDPDCIK
QILFNYGSKYIKGTIAEAGEFLFGLGVALQENEAWKIRRKAVAPSLHRRYVEAMVDRCFGPCAERMVELVESAID
SEGGEKKRLNMESKFSQAALDIIGISVFNYDFKALTSAAPVIQATYTALKEVETRSMDLLPTWRLPEPFLRVVSPR
QKAAQDAVKIIQEVTTKLVDDCKRMVEEEEKVGGAEEWARDYLNDSNPSVLRYLIAAREEVSSTQLRDDLLSLLV
AGHETTASVLTWGTYELLKPENAEQLRLLRAELDEVLGDKPFPTYEDMTKMPYLERCFHESMRLYPQPPVYTRR
AVVEDVLPKGLGVVPAGQDLLVSIYNLHRSPENWGPNSQVFEPMRFGPLALGQPNELNTGYRYTPFSAGPRRC
PGDKFAVLEGMAIWAVMFRRLDLTLVAGHDVVMTSGATIHTRDGMLVNARRRRGEKGQKVDWAQLKPEKDI
GAGWFEKGIEAKGSSGGGKCPVAH
>LUT-39
MSRMPVAAGDIREIAGQPVFVPLYKLFLAYGEMFILAIGPKKFVVVSDNEVAKEMLLTQANSFSKGLLSEILDFV
MGTGLIPANGETWKIRRRTVVPSLHKKYVASMVDMFGDCGVHGSAQLAKSEREGKTVEMENFYSRLALDIIG
KAVFNYDFDSLKKDDPVIKAVYTVLREAEYRSVTFIPYWKVPPLRWLVPRQKACQEALVVVNDTLNMLIERTKKI
VEDSDEEFVEEYLSGDDPSILNFLIASGDDVTSKQLRDDLMTLLIAGHETTAAVLTWTTYLLATHPEQMRKVQEE
VDRVVGDRRPTIQDMMELKYTTRVINESMRLYPQPPVLIRRALEPVTLDGYKIETGTDFFISVWNLHRNPRLWP
EPDKFIPERFPLDQKMPNEVTENFAYLPFGGGQRKCVGDQFALFESIITLAMVCRRFDIDLDPAFHPDGECGMT
TGATIHTTGGLHVKLTRRSRTQPAACPAPPASPASTTRAPPSSAASVPRPPPPGSAFPPPGASPSPSSQPADLRTR
PPP
>LUT-40
MPQPPSTEEDIDDDFVPSQLGIKDIISLWITQILQTYGDEESKDGAPVCEGSVDDLVGGPIFLALYPYFLRYGGVFK
LAFGPKVFMVLSDPVVVREVLKEKPFAFSKGVLAEILEPIMGQGLIPAPYAVWKNRRRQLVPGFHKAWLDHMV
GLFGDCSTQLVKNLDAEIAKGNGSAIVDMEERFCSVSLDIIGLAVFNYDFGSTTRESPIIKAVYTCLQEAAHRSTFY
FPYWNLPLADVLVPRQREFKNNMNLINETLNGLIKKAQAFEGTEDLEELQNRDYSKVKDPSLLRFLVDIRGADV
TDSQLRDDLMTMLIAGHETTAAVLTWCLYCLAQDRELMARVVAEIDDVMGPADGETPTAPNYEQIQKMELVR
LCLAEALRLYPEPPILIRRCLEDVPLPKGAGDANVTLIKGMDVFISVWNLHRHPDCWEEPLKFDPTRFKRPFQNP
GVKDWAGYNPDLISGLYPNEVTSDFAFIPFGAGARKCIGDQFAMLEATSCLAMTLRRYDFEMTKDASEVGME
MGATIHTAGGLPMKVTRR
>LUT-41
MPAPTRFTRAPRRAHPGSRPAERGARVARRAVEEPKTRGGDSAPGRIYRGALFPGVEVPEAGADVWRGISRVF
PWGNGAPVTEGVLGDLLKEEVRAAPLFVPLYDYYRQYGGVYNLGAGPKWFVVVSDPVVVRHMFKDNADAFS
KGILTDIMEPIMGDGLIPAPKEIWAKRRPTVGAGFHGAWLKHMTNLFGASATNLADKLEREWCDKDVAVNLE
DELYAMALDVIGKAVFNYEFGALREETPLIKAVYRVLRESEHRSTFPLQYWNIPGAMDVVPRQKQFKEDIAAINA
ELSKLIADALADRNETDLAEMESRDYANVEDASLLRFLVDVRGETVSSTQLRDDLMTMLIAGHETTAAVLTWT
MYLLATHPEEAELARAEVDAIVADPSGVPTVEEIRKLERTRLCLAEGMRMYPAPPILIRRALEDVTLPAGGMGRE
ITLKKGTDCFVAVWNLHRSPDLWDRPDVFDPARFKREFKNPKIEGWNGLSPELVTGLYPNEQSTDFAYVPFGGG
QRRCAGDMFAMMEATVALSVLLKRFEFELGCDESEVEMITGATIHTKAGMPVKLRSRSAK
>LUT-42
PKAESLAEPRKIKRLTRGEREKMGETYEWTAWVSTCGVVSIAITATYLRLLREVSDTGAFPWSELVAQLALIAGAA
VGMEFYARYAHKYLWHDSLPIWLLHESHHLPREGAFEANDIFAIANGVPAFALCAYGFLTPGLFGGLCFGAGLGI
TLFGIAYMYVHDGLVHKRFPTGPLGKLPFLRKIAAGHTIHHTEAFDGVPWGLFLGIQELEQVPGGLAELEAVME
AADRKSA
>LUT-43

APDWMTQLNRLWGGESEIPVADAKLEDITGLLGGGLFQPLFKWMRESGSVYLLPTGPITSYVVVSDPACIKQVL
FNYGSKYIKGTIAEAGEFLFGLGVALQELEPWKIRRKAVAPSLHRKYVEAMVDRCFGPCADRMVTMLESESASG
PVGGVNMESRFSKTALDIIGISVFNYDFEALTTAAPVIQATYTALKEVETRSMDLLPTWRLPEQFLRAVSPRQKAA
QDAVTIIRDVTQKLVDDCKRMVEEEEKVGGAEEWARDYLNESNPSVLRYLIAAREEVSSTQLRDDLLSLLVAGHE
TTASVLTWGTYELLKPENAEQLRLLRAELDEVLGTRPYPTFADLPNMPYLERCFHESMRLYPQPPVYTRRAVVED
VLPNGMTVPKNQDLLVSIYNLHRSPDNWGPTSQEFEPMRFGPLANGQPNELNTDYRYVPFSAGPRRCPGDKF
AVYEGIVIWATMIRRLDLELKPGHDVVMTSGATIHTKNGLLATV

>LUT-44
MSKMPVASGDIREIAGQPVFVPLYKLFLAYGEMFVLAIGPKKFVVVSDNAVAKEVLLTQAKSFSKGLLSEILDFV
MGQGLIPANGEVWKMRRKVIVPSLHKKYVTSMVDMFGDCGLKGMSQLARAETAGESVEMENFYSRYALDII
GKAVFNYDFDSLTTDDPVIKAVYTVLREAEYRSVTFIPYWKVPPLRWLVPRQRKCQEALEVVNDTLDELISRCKK
VVEEEDEEFVEEYMNTDDPSILHFLIASGDDVTSKQLRDDLMTLLIAGHETTAAVLTWTTFLLAKHPEVKAKVFE
EVDRVVGDRNPTVADMRELVYTTRVINESMRLYPQPPVLIRRALEPVSLGGYNIDAGTDFFISVWNLHRNPRI
WDEPDAFKPERFPVEAPMPNEYTEEYAYLPFGGGQRKCVGDQFAIFESIVSLAMLMRRFDVELDASKHPDGEC
GMTTGATIHTTNGLHVKLTRRQGRGGAEMRGVYTEGKQTLSNLEDVDVAGGSIDAPSASADEFDEAVGEDDD
DIADTKEKLERATRIVESEKEEVGQAR

>LUT-45
MPSDDFTPEELKFQDVVSLWVTQILQTYGDAESKDNAPVCEGVIDDLVGGPIFLALYPYFRRYGGVFKLAFGPKV
FMVLSDPVVVREVLKEKPFSFDKGVLAEILEPIMGQGLIPAPYAIWKNRRRQLVPGFHKAWLDHMVGLFGHCS
GELVRNLDAAAAAGETVDMEERFCSVSLDIIGLAVFNYDFGSVTKESPIISAVYNCLQEAAHRSTFYFPYWNLPFA
TDIVPRQREFKQNMAVINDTLNGLIAQAQQYEGTDDLEELQNRDYSKVKDPSLLRFLVDVRGADVTDAQLRDD
LMTMLIAGHETTAAVLTWGLFCLVQKPELMKRIQADIDEVFGDDDRAPTYEDIQRLESVRLCVAEALRLYPEPPI
LIRRCLEDVTLPKGAGDREITLIKGMDIFISVWNLHRSPECWENPDEFDPFRFKRPFANPGVKDWAGYDPDLLT
GLYPNEIASDYAFIPFGAGARKCIGDQFAMLESTVAMTMTLRDFDFELQKNPEDVGMEMGATIHTAGGLPMK
VTRRRR

>LUT-46
MASMPSPTRLPRVATTTTTTTRRVRRDEPSSGHRARVRARALDSKPPAASDAYRGALFPGVEVPDNDLARSFA
RLFPWGNGARVTEKVLGDLLKEEVRAAPLFVPLYDYYREYGGVYNLGAGPKWFVVVSDPVAVRTMFKDRADS
FSKGILTDIMEPIMGDGLIPANKEIWAKRRPVIGAGFHNAWLKHMCDLFGASAMRLADKLDAAAETKETVELE
GELYSMALDVIGKAVFNYEFGALREETPIIKAVYRVLRESEHRSTFPLQYWQIPGAMDVVPRQKQFKEDMKMIN
EELSVLINNAISSRTETDLEEMERRDYSKVEDASLLRFLVDIRGDEATSTQLRDDLMTMLIAGHETTAAVLTWTLY
LLAQHPEIAEEAVEEINACVSDSNGVPTPEEVRKLQKVRMILAEGMRLYPAPPILIRRAIEDVTLPRGGNGKEITLK
AGTDCFIAVWNLHRSPDLWENPEKFDPSRFSRRFENPAIEGWGGLNPDLMTGLYPNEQSTDFSYVPFGGGQR
RCAGDQFAMLEAVTAMSVLLKKFDFKLACSPSDVEMITGATIHTKKGLPMKIKRRATR

>LUT-47
ARYAHKHLWHGPWWSMTSKYRREWNKRIWLLHESHHLPREGAFEANDIFALVNGVPAFALCAFGFFKPGVF
GGLCFGAGLGITLYGIAYMYVHDGLVHKRFPTGPLGKLPLMRKIAAGHTIHHTEAFEGVPWGLFLGIQELEAVEG
GIEELNK

>LUT-48
MDEEIDLDVSVDRGLQKLIDTAGAGASVTSPGWLTQLGRLWGGKSDVPVADAKPDDIKDLLGGALFKALYKW
MEETGPVYLLPTGPVSSFLVVSDPEAAKHVLRATDNPKRPIYVKGLVAEVSEFLFGDGFAITGGDNWRVRRRAV
GPSLHRAYLAAMADRVFGPSAQHLATKLEGVAASGESIDMEACFSQLTLDVIGKAVFNYDFDALNTQSPLIQAV
YTALKETEQRATDLLPLWKLPFLAPFVPRQRKALEAVELIRAETERLIAKCKEMVDAEEQAQFGDGYMNEADPS
VLRFLIASREEVSSRQLRDDLLSMLVAGHETTGSVLTWTLYLLEQNPRAMAKARAEVDAVMGDRAAPSVEDFM

ALRYVMRCVNESMRLYPHPPVLLRRAQVADTLPGGYSVPVGQDVMISVYNIHRSPAVWDDPNDFRPERFPLD
EPVPSEQTTDYRFIPFSGGPRKCVGDQFALMEAVVALAVLLKRFDFELVPDQDIGMTTGATIHTQNGLFMTVRE
RASGRSQGSSSNGASSLEPAVAAGAAA
>LUT-49
MQCFWSTGRGLAYQLAKIGQEWRAAAAVRMPEARGDIREIVGQPVFVPLQKLAMIYGKVFRLSFGPKSFVVV
SDAAVARHIMMTNAANYSKGILSEILDFVMGSGLIPADGEVWKARRRVVVPSLHRKYIANMVDMFADSALHG
VATLAAAQEARILLSAGRAVEMENFFSRLTLDIIGKAVFNYDFDSLTHDDPLCLYLCQAVYTVLREAEYRSTYPLPY
WQLPLMMWLVPRQRQCVEALRIINTTLDSLIAKSKRLFDEEDQEFGEDYLSDKDPSILHFLLASGDEITSKQLRD
DLMTMLIAGHETTAAVLTWTLFCLTDRPDVLRRMQQEIDEVVGDGKPSMDQLRSLRLTTRVVNEAMRLYPQP
PVLIRRALEDDVLGGFHVSAGSDIFISVWNIHRDTGYWTRPEDFDPDRFSLEGPVPNETTENFNYLPFGGGRRK
CIGMHFLLRPISDQFALFESLSALAVLLRRFEFRMAPGAPPVNMTTGATIHTSEGLLMQVVPRTITDPLEGVPVN
GKLVITPAAATLSASTV
>LUT-50
MLTELSQGYFQPNVGGETIPVAQGELSDLAGDEPLFKALYQWFLDSGGVYKLAFGPKAFIVVSDPVVVRHLLKE
NAFNYDKGVLAEILEPIMGKGLIPADLETWKPRRRAIVPAFHKAYLETMVAMFGACTQETIRSLDALTADGEGQ
TDMEEVFLSLGLDIIGLGVFNYDFNSITKESPVIKAVYGVLKEAEHRSTFYIPYWNIPITKYIVPRQRKFNADLAVIN
ACLDDLIAQAKQTRQADDVEALQARDYSKVRDPSLLRFLVDMRDADLSDKQLRDDLMTMLIAGHETTAAVLT
WTLFALLTHPSAYAKVLAEIDSVLGDKTPSIEDMRAMPYVRCALAESLRMYPQPPILIRRALSDDVLPAPLGGDSS
GYPIGKGADIFISSSSGTIMFQSLHRSPHLWKDPDTFRPERFSETNSNPAFNGAWAGYRPEAQGSSFYPNEVAS
DFAFIPFGGGARKCVGDQFALLEATVALTMLLRRFTFDFVEGPESVGMATGATIHTANGLQVRVQRRDVGVTPA
GAESQEVAVPV
>LUT-51
MVFSSHTSASYGGIVGRGVTVGHRCNSGAQPRASHVTYRRRQSQRCIVSPSSIDLQRVSVTGMPCAPEAEECS
VSDRFSVELRGQDSIAEALSAGSSGILDAASGAAQDVTAAVQAVAQDALTARRFERQQSRAREQTTYKMAAILA
STGITAMAITAVHLRFTWHMRDGAEFPILEAAATLLLTFGGVVGMEMWARWAHKALWHENEAGWSLHKSH
HVPRVGPFEANDIYAIANAVPAMGLCLYGFLTPTLMGGVCFGAGLGITLFGIMYMFIHDGLVHRRFPVGPLADV
PYLKRCAVAHQLHHSEKYGGIPWGMFLGPQELEAVGAKEELDRMCEELTFSSKKN
>LUT-52
MDEEIDLDVSVDRGLQKLIDTAGAGASVTSPGWLTQLGRLWGGKSDVPVADAKPDDIKDLLGGALFKALYKW
MEETGPVYLLPTGPVSSFLVVSDPEAAKHVLRATDNPKRPIYVKGLVAEVSEFLFGDGFAITGGDNWRVRRRAV
GPSLHRAYLAAMADRVFGPSAQHLATKLEGVAASGESIDMEACFSQLTLDVIGKAVFNYDFDALNTQSPLIQAV
YTALKETEQRATDLLPLWKLPFLAPFVPRQRKALEAVELIRAETERLIAKCKEMVDAEEQAQFGDGYMNEADPS
VLRFLIASREEVSSRQLRDDLLSMLVAGHETTGSVLTWTLYLLEQNPRAMAKARAEVDAVMGDRAAPSVEDFM
ALRYVMRCVNESMRLYPHPPVLLRRAQVADTLPGGYSVPVGQDVMISVYNIHRSPAVWDDPNDFRPERFPLD
EPVPSEQTTDYRFIPFSGGPRKCVGDQFALMEAVVALAVLLKRFDFELVPDQDIGMTTGATIHTQNGLFMTVRE
RASGRSQGSSSNGASSLEPAVAAGAAA
>LUT-53
MPEARGDIREIVGQPVFVPLQKLAMIYGKVFRLSFGPKSFVVVSDAAVARHIMMTNAANYSKGILSEILDFVMG
SGLIPADGEVWKARRRVVVPSLHRKYIANMAGRAVEMENFFSRLTLDIIGKAVFNYDFDSLTHDDPVIQAVYTV
LREAEYRSTYPLPYWQLPLMMWLVPRQRQCVEALRIINTTLDSLIAKSKRLFDEEDQEFGEDYLSDKDPSILHFLL
ASGDEITSKQLRDDLMTMLIAGHETTAAVLTWTLFCLTDRPDVLRRMQQEVNEAMRLYPQPPVLIRRALEDDV
LGGFHVSAGSDIFISVWNIHRDTGYWTRPEDFDPDRFSLEGPVPNETTENFNYLPFGGGRRKCIGDQFALFESLS
ALAVLLRRFEFRMAPGAPPVNMTTGATIHTSEGLLMQVVPRTITDPLEGCSAFVRCLKS
>LUT-54

MLTELSQGYFQPNVGGETIPVAQGELSDLAGDEPLFKALYQWFLDSGGVYKLAFGPKAFIVVSDPVVVRHLLKE
NAFNYDKGVLAEILEPIMGKGLIPADLETWKPRRRAIVPAFHKAYLETMVAMFGACTQETIRSLDALTADGEGQ
TDMEEVFLSLGLDIIGLGVFNYDFNSITKESPVIKAVYGVLKEAEHRSTFYIPYWNIPITKYIVPRQRKFNADLAVIN
ACLDDLIAQAKQTRQADDVEALQARDYSKVRDPSLLRFLVDMRDADLSDKQLRDDLMTMLIAGHETTAAVLT
WTLFALLTHPSAYAKVLAEIDSVLGDKTPSIEDMRAMPYVRCALAESLRMYPQPPILIRRALSDDVLPAPLGGDSS
GYPIGKGADIFISSSSGTIMFQSLHRSPHLWKDPDTFRPERFSETNSNPAFNGAWAGYRPEAQGSSFYPNEVAS
DFAFIPFGGGARKCVGDQFALLEATVALTMLLRRFTFDFVEGPESVGMATGATIHTANGLQVRVQRRDVGVTPA
GAESQEVAVPV

>LUT-55

MVFSSHTSASYGGIVGRGVTVGHRCNSGAQPRASHVTYRRRQSQRCIVSPSSIDLQRVSVTGMPCAPEAEECS
VSDRFSVELRGQDSIAEALSAGSSGILDAASGAAQDVTAAVQAVAQDALTARRFERQQSRAREQTTYKMAAILA
STGITAMAITAVHLRFTWHMRDGAEFPILEAAATLLLTFGGVVGMEMWARWAHKALWHENEAGWSLHKSH
HVPRVGPFEANDIYAIANAVPAMGLCLYGFLTPTLMGGVCFGAGLGITLFGIMYMFIHDGLVHRRFPVGPLADV
PYLKRCAVAHQLHHSEKYGGIPWGMFLGPQELEAVGAKEELDRMCEELTFSSKKN

>LUT-56

DIGELFGGALFLVLKRFQELYGSAFSPPPGPTSSFLVLSDPASAKHVLNSYKTYEKGLVREISEFLFGDGFAVADGEK
WKIRRRAVSPSLHRKYLDKMVSRVFADCSLRTSKVVQSYRGPAQPSNSEVNMEKVFSELTLDIIGKAVFNYDFNS
IGSTEDNEVISAVYAALKETERRSLDFLPVWKLGDDVARSVSPRQREAQDAVALIRKTTEDLIENCRKQLEEATGV
AGSFNEDDYISESDPSVLRFLIASREEVTSSQLRDDLLSMLVAGHETTGSVLTWTLYLLTKHPEKMNRLQEEIDSIL
GSKLDSEEGVSSLTLDDMLSLNYLKWCVAESMRLYPHPPVLIRRALEDDTLPSGYKVPKGQDVMISIYNVHHSPS
VWDDPDSFQPERWYGPRDNPNEKNTNFHYIPFSGGARKCIGDQFALLEAYTALSVLVRKFDFQLVKGQNIGMT
TGATIHTTEGMRMTYQPRM

>LUT-57

MQIFKANIEEIYGGYPSKDGTPIATGSLDGLTDGTMYLALNAFQQEFGPIYKLCFGPKSFIVVSDPVITKEILKSPPT
AYDKGVLAEILEDIMGKGLIPADPVIWKQRRRAIVPGFHKRWLDGMISVFGRASSTLIEDLDVAATTGEPRDME
ERFGSVALDIIGKAVFNYEFESTQKTSPVVKAAIDTLREAEHRSMIPLPYWKLPLADRLIPRQRAFASNMALMNE
KLDTAIEAALSSRDEADLETIENRDYNAMENPSLLRFLVDMRDEPTSSRQLRDDLMTMLIAGHETTASALTWAL
FELMQQPELLQKIRKEVDEVIGDRAPTYEDIVNMPLVRVTIAESLRMYPEPPLLIRRALEETVLPKGGAEQETKLP
RGADIFISSYNLHRSPLFWEEPDRFNPERFFKPFKNPEAAPYWKGYDPKLWKGRLYPTEDSTDFAYIPFGGGSRK
CVGDVFAMLEATVALAMILRRYDFDFTAPTSEPSMVGTNTGATIHTRNGLWCKVSPRQYQDNKPSSSSSSSSS
SSPAVNAPSSEETVAAAM

>LUT-58

MTFGDTANVLRTNIEQIYGGFPSLDGCPLAEGELGDIADGTMFIGLQNYYKKYGSPYKLCFGPKSFLVVSDPVQA
RHLLRDANKNYDKGVLAEILKPIMGKGLIPADPETWRVRRPQIVPAFHRKWLEYMVGQFGYCNKPLIDSLNILA
DTTGKVEMEEKFCSVALDIIGKSIFNYDFGSTTDESPVIKAVYSALVEAEHRSMTPAPYWDIPLANQLVPRLRKFN
TDLKLLNDVLDELIDKAKRTRTVGDIEQLESRNYAEAKDPSMLRFLVDMRGADIDNKQLRDDLMTMLIAGHETT
AAVLTWSLFELSKHPEILATVVEEIDRVVGDRAPNLDDIKEMKQVRLVIAETLRMYPEPPLLIRRCRTEDKLPAGG
GREATVIRGMDIFLPLYNIHRDERFWPNPDTFDPQRFTRPYKNPDIPDWAGFDPEKWSKMLYPNEVASDFAY
MPFGAGARKCVGDEFAIMEAVVTLSMVLRRFDFEFDLSKSTSVDMYDPPQTADHPVGIRTGATIHTKNGLNML
VKKRQTN

>LUT-59

MREDGAWVKKPSLFEFLVSKHRINNGSITGPSPVNVMDVAFLFAVTILSYLGLGPAFGMAAVPTAVIQKYEGSM
LSFITGVLGGDLQTMAGGPLFLLLNKYFLENGPIFNLSFGPKSFLVISDPVMAKHILRTAPADQYCKGMLADILEPI
MGKGLIPADPATWKVRRRAVVPAFHKRWLKRMITLFNERAEILCDDLTRKEGTVIDMEERFCSVTLDIIGKAVFD

YDFGSVTKESPIIKAVYRVLREAEHRSSSFIPYWNLPYADQWMGGQVEFRTDMTMLDDILAKLINRAVSTRSELT
VEELEDRDNAEDPSLLRFLVDMRGEDVSSTVLRDDLMTMLIAGHETTAAMLTWTLFELAQGEPGMFEEIQNE
VRTVLKDKDRPDYDDVVAMKKLRYALIEGLRLYPEPPVLIRRARTEDILPQGSSVMKDGIKVLRGTDIFVSTWNL
HRSPELWENPLTFDPTRWERSFKNPNVKGWAGYDPDKVSGLYPSENAADFAFMPFGGGSRKCVGDQFAMLE
AAVTFSVIIKNFNFEFEGSPEDVGMQTGATIHTMNGLRMRQTRVKKDDPLPSTDGWWEKQHLKRGLSSNGRA
YQSKEEVEHTKFTEITDLLTLE
>LUT-60
MRIFKGNIEQIYGDAPSFDGAPLAEGDISGLADGTLYLGLHEYSQRFGPVYKLCFGPKSFIVVSDHAVAKHVLREN
NGGYNKGVLAEILEDIMGKGLIPADPVTWKARRRAIVPAFHKRWLARMLTMFADETELLNAELPLGEPVDLEER
FGSLALDIIGSAVFNYDFDSVREPSRVVKAAIDTLREAEHRSMTPAPYWKIPGAMQVVPRQRAFTENMDLLNG
ELNKAIAAALADRVEEATEELERRDYATMENPSLLRFLVDQRGEEATSTQLRDDLMTMLIAGHETTASALTWCLF
ELAQNRPLLEELRAELDAKLPGGRPPRTLDEVRAVELTRLTVAESLRMYPQPPLLIRRAVDDDAVPTVQLPDTDE
LDASGLRARAVDVKVPRACDMFIAIYSLHRNPRYWKNPDSFDPKRWLEKYANPDEPTWAGYDPAKWKAGDG
MGSLYPTETSADFAYLPFGGGARKCVGDQFAMMEATVALAGFLQRFDFDFAGPTDTPDKVGTNTGATIHTRN
GLWMTVTERAK
>LUT-61
MAKSIATGDLQTLVGGPLFLLLTKYHNELGPVFKLAFGPRSFIVVADPSIMRYILRDGAMNYDKGILAEILAPILGN
GLIPADPDVWRRRRRVITPAFHKQWLASTLSLFDECTMELVDDLKARHATAEPAPLPATVDAWTAWKADDAQP
RALGAVDMEERFCSVSLDIIGRAVFDYDFGSAKAESPLVRAVYRCLIEAEKRTTAFIPFWLIPGAQFLPSQVAFKN
DFDLLNAKLDELVAQAFEEQIDDDDMLEAGQEVTKASTERISLLRFLVTIRGEEASTGQLRDDLMTMLVAGHET
TAALLTWTLYELFHPSKRAAGHLERLRAEVDANFALRKSENRTATAYADVVDCAFARLCLAEGLRLYPQPPLLIRRA
LDSDELPQPYADGRRTAPPPPSGPPSRSSPRSSPPPRSRRALQPRRMLSSSEPG
>LUT-62
MNSLLFFLSVSLTLVSLIGYVMHMPLSVCFVFNVISLHLAGSLIHDASHKSAHSNEYINGIIGHVCGFLLGFSFVVF
KKVHMQHHAHVNQAKYDPDHYVSTGGPIWLIAPRFFYHEIYFFQRRLYRNHELLEWMMARGLFFLVLMAAW
KFGALTYVLRCWFCAALLVGTFLGLCFDYLPHYPFVQTHRWHNACIQENDMLNWLILGQNYHLVHHLWPSEP
WYKYQQKYQAHQQLFTPQTCVLGWPTQIGYDLCFGLRIG
