# Supplementary material for: Evolutionary origins, molecular cloning and expression of carotenoid hydroxylases in eukaryotic photosynthetic algae
Source: BMC Genomics. 2013 Jul 8;14:457. doi: 10.1186/1471-2164-14-457 (PMC3728230; doi:10.1186/1471-2164-14-457)
Supplement: Additional file 4: Figure S1 — The multiple sequence alignment of all CYP97 from algae. The names of each sequence are as listed in Additional file 2. A partial protein sequence (position: 272–926) has been selected for domain structure analysis. [file 1471-2164-14-457-S4.pdf]

Evolutionary origins, molecular cloning and expression of carotenoid hydroxylases in eukaryotic photosynthetic algae

Hongli Cui[1, 2§], Xiaona Yu[3§], Yan Wang[2], Yulin Cui[2], Xueqin Li[4], Zhaopu Liu[3] and Song Qin[1*]

[1]Key Laboratory of Coastal Biology and Biological Resources Utilization, Yantai Institute of Coastal Zone Research, Chinese Academy of Sciences, Yantai 264003, People’s Republic of China

[2]University of the Chinese Academy of Sciences, Beijing 100049, People’s Republic of China

[3]College of Resources and Environmental Sciences, Key Laboratory of Marine Biology, Nanjing Agricultural University, Nanjing 210095, People’s Republic of China

[4]Shenzhen Key Laboratory for Marine Bio-resource and Eco-environment, College of Life Sciences, Shenzhen University, Shenzhen 518060, People’s Republic of China

[§]These authors contributed equally to this work.

[*]Corresponding author

E-mail addresses:

HLC: hlcui@yic.ac.cn

XNY: 2011103006@njau.edu.cn

YW: ywang@yic.ac.cn

YLC: yulincui@yic.ac.cn

XQL: 2110180316@email.szu.edu.cn

ZPL: sea@njau.edu.cn

SQ: sqin@yic.ac.cn

Additional file 4 – Figure S1 The multiple sequence alignment of all CYP97 from algae

The names of each sequence are as listed in additional file 1. A partial protein sequence (position: 272–926) has been selected for domain structure analysis.

```
LUT-1   1   GALFKALYKWMQESGPIYLLPTGPVSSFLVVSDPAAAKHVLRSTDNSQRNIYNKGLVAEVSEFLFGKGFAISGGDAWKARRRAVGPSLHK
LUT-6   1   GALFKALYKWMQESGPVYLLPTGPVSSFLVISDPAAAKHVLRATDNSQRNIYNKGLVAEVSQFLFGKGFAVAGGDDWKVRRRAVGPSLHR
LUT-48  1   GALFKALYKWMEETGPVYLLPTGPVSSFLVVSDPEAAKHVLRATDNPKRPIYVKGLVAEVSEFLFGDGFAITGGDNWRVRRRAVGPSLHR
LUT-52  1   GALFKALYKWMEETGPVYLLPTGPVSSFLVVSDPEAAKHVLRATDNPKRPIYVKGLVAEVSEFLFGDGFAITGGDNWRVRRRAVGPSLHR
LUT-31  1   GALFKALFKWMVESGPVYLLPTGPISSFLVISDPECAKHVLRASDNPSRPIYEKGLVAEVSQFLFGEGFAITGGEQWRVRRKAVGPALHR
LUT-56  1   GALFLVLKRFQELYGSAFSPPPGPTSSFLVLSDPASAKHVLNSYK-----TYEKGLVREISEFLFGDGFAVADGEKWKIRRRAVSPSLHR
LUT-14  1   GGLFQPLFKWMRESGPVYLLPTGPITSYVVVSDPDCIKQVLFNYGS----RYIKGTIAEAGEFLFGLGVALQELEPWKIRRKAVAPSLHR
LUT-43  1   GGLFQPLFKWMRESGSVYLLPTGPITSYVVVSDPACIKQVLFNYGS----KYIKGTIAEAGEFLFGLGVALQELEPWKIRRKAVAPSLHR
LUT-20  1   GGLFQPLFKWMLEAGPVYLLPTGPVTSYVVVSDAACIKQVLFNYGS----KYIKGTIAEAGEFLFGLGVALQELEPWKVRRKAVAPSLHR
LUT-26  1   GGLFQPLFKWMKEAGPVYLLPTGPITSYVVVSDPDCIKQVLFNYGS----KYIKGTIAEAGAFLFGLGVALQENEAWKIRRKAVAPSLHR
LUT-38  1   GGLFQPLFKWMKEAGPVYLLPTGPVTSYVVVSDPDCIKQILFNYGS----KYIKGTIAEAGEFLFGLGVALQENEAWKIRRKAVAPSLHR
LUT-2   1   QPVFVPLYKLFLVYGKIFRLSFGPKS-FVIISDPAYAKQILLTNAD----KYSKGLLSEILDFVMGTGLIPADGEIWKARRRAVVPALHR
LUT-7   1   QPVFVPLYKLFLVYGKIFRLSFGPKS-FVIISDPAYAKQILLTNAD----KYSKGLLSEILDFVMGTGLIPADGEVWKARRRAVVPALHR
LUT-49  1   QPVFVPLQKLAMIYGKVFRLSFGPKS-FVVVSDAAVARHIMMTNAA----NYSKGILSEILDFVMGSGLIPADGEVWKARRRVVVPSLHR
LUT-53  1   QPVFVPLQKLAMIYGKVFRLSFGPKS-FVVVSDAAVARHIMMTNAA----NYSKGILSEILDFVMGSGLIPADGEVWKARRRVVVPSLHR
LUT-15  1   QPVFVPLYKLFLAYGEMFVLAIGPKK-FVVVSDNAVAKEMLLTQAK----SFSKGLLSEILDFVMGQGLIPANGEVWKIRRKVIVPSLHK
LUT-44  1   QPVFVPLYKLFLAYGEMFVLAIGPKK-FVVVSDNAVAKEVLLTQAK----SFSKGLLSEILDFVMGQGLIPANGEVWKMRRKVIVPSLHK
LUT-16  1   QPVFVPLYKLFLAYGEMFVLAIGPKK-FVVVSDNAVAKEMLLTQAK----SFSKGLLSEILDFVMGQGLIPANGEVWKIRRKVIVPSLHK
LUT-21  1   QPVFVPLYKLFLAYGEMFVLAIGPKK-FVVVSDNAVAKEMLLTQAK----SFSKGLLSEILDFVMGQGLIPANGEVWKIRRKVIVPSLHK
LUT-27  1   QPVFVPLYKLFLAYGEMFVLAIGPKK-FVVVSDNDVAREMLKDQAT----SFSKGLLSEILEFVMGTGLIPADGETWKVRRRTVVPSLHK
LUT-39  1   QPVFVPLYKLFLAYGEMFILAIGPKK-FVVVSDNEVAKEMLLTQAN----SFSKGLLSEILDFVMGTGLIPANGETWKIRRRTVVPSLHK
LUT-3   1   VPIFQLLYELYSSHGGVFRMRLGPKS-FLVLSDPGAVRQVLVGAVD----KYSKGILAEILEFVMGNGLLAADGEHWIARRRVVAPALQR

LUT-1   91  AYLEAMLDRVFGASSLFA------ADKLR----KAAAEGTP-------VNMEALFSQLTLDIIGKSVFNYDFNSLTS--DSP----VIQA
LUT-6   91  AYLEAMLVRVFGPSSEFA------ADKLR----VAARSGTP-------VNMEAMFSQLTLDIIGKAVFNYDFNSLTS--DSP----LIQA
LUT-48  91  AYLAAMADRVFGPSAQHL------ATKLE----GVAASGES-------IDMEACFSQLTLDVIGKAVFNYDFDALNT--QSP----LIQA
LUT-52  91  AYLAAMADRVFGPSAQHL------ATKLE----GVAASGES-------IDMEACFSQLTLDVIGKAVFNYDFDALNT--QSP----LIQA
LUT-31  91  GYLEVMLDRVFGESALHL------NKKLE----VAAASGEP-------IDMEACFSQLTLDVIGKAVFNYDFDALNN--NTP----VIQA
LUT-56  86  KYLDKMVSRVFADCSLRT------SKVVQSYRGPAQPSNSE-------VNMEKVFSELTLDIIGKAVFNYDFNSIGSTEDNE----VISA
LUT-14  87  KYVEAMVDRCFGPCADRM------VSILEG--EAGAGGVGG-------VNMESRFSKTALDIIGISVFNYDFEALTT--AAP----VIQA
LUT-43  87  KYVEAMVDRCFGPCADRM------VTMLES--ESASGPVGG-------VNMESRFSKTALDIIGISVFNYDFEALTT--AAP----VIQA
LUT-20  87  KYVEAMVDRCFALCADRM------TTILEE--EAANGAVGS-------VNLESRFSKTALDIIGISVFNYDFKALTT--AAP----VIQA
LUT-26  87  RYVEAMVDRCFGPCADRM------VSLVEDQINADGGRRER-------VNMESKFSQAALDIIGISVFNYDFKALTS--AAP----VIQA
LUT-38  87  RYVEAMVDRCFGPCAERM------VELVESAIDSEGGEKKR-------LNMESKFSQAALDIIGISVFNYDFKALTS--AAP----VIQA
LUT-2   86  KYVMSMVD-MFGDCAAHGA-----SATLD----KYAASGTS-------LDMENFFSRLGLDIIGKAVFNYDFDSLAH--DDP----VIQA
LUT-7   86  KYVASMVG-MFGDCTVHG------TATLD----CAVASGQS-------IDMENYFSRLALDIIGKAVFNYDFDSLTH--DDP----VIQA
LUT-49  86  KYIANMVD-MFADSALHGVATLAAAQEAR----ILLSAGRA-------VEMENFFSRLTLDIIGKAVFNYDFDSLTH--DDPLCLYLCQA
LUT-53  86  KYIANM-------------------------------AGRA-------VEMENFFSRLTLDIIGKAVFNYDFDSLTH--DDP----VIQA
LUT-15  86  KYVTSMVD-MFGDCGLKG------MSQLA----RAEKANES-------VEMENFYSRFALDIIGKAVFNYDFDSLST--DDP----VIKA
LUT-44  86  KYVTSMVD-MFGDCGLKG------MSQLA----RAETAGES-------VEMENFYSRYALDIIGKAVFNYDFDSLTT--DDP----VIKA
LUT-16  86  KYVTSMVD-MFGDCGLKG------MSQLA----RAEKANES-------VEMENFYSRFALDIIGKAVFNYDFDSLST--DDP----VIKA
LUT-21  86  KYVTSMVG-MFGDCGLKG------MAQLA----RAEKMGES-------VEMENFYSRFALDIIGKAVFNYDFDSLTT--DDP----VIKA
LUT-27  86  KYVASMVD-MFGDCGLNG------SAQLA----RSEMNGDT-------VEMENFYSRLALDIIGKAVFNYDFNSLKM--DDP----VIKA
LUT-39  86  KYVASMVD-MFGDCGVHG------SAQLA----KSEREGKT-------VEMENFYSRLALDIIGKAVFNYDFDSLKK--DDP----VIKA
LUT-3   86  KFVSSQVA-LFGAATAHG-LPQLEAAAAA----AAAAAGDSRGGGAASVDMESFFSRLSLDIIGKSVFDYDFDSLRH--DDP----VIQA

LUT-1   158 VYTALKETEQRATDLLPLWKV--RGIGWLIPRQRKALEA--VELIRKTTN-DLIKQCKEMVDEEEMRAASA----AAAAGTEYLNEADPS
LUT-6   158 VYTALKETEQRATDLLPLWKV--PALGWLIPRQRKALQVRHSTLVRRPVKKNLFSRSVCVCVCFAFVVLLC----AAANGDFTAKQQHQC
LUT-48  158 VYTALKETEQRATDLLPLWKL--PFLAPFVPRQRKALEA--VELIRAETERLIAK-------CKEMVDAEE----QAQFGDGYMNEADPS
LUT-52  158 VYTALKETEQRATDLLPLWKL--PFLAPFVPRQRKALEA--VELIRAETERLIAK-------CKEMVDAEE----QAQFGDGYMNEADPS
LUT-31  158 VYTALKETETRATDLLPYWKY--PLINLFVPRQRKAAAA--VELIRQTTEELIAK-------CKAMVDAEE----AASFEEGYINDADPS
LUT-56  159 VYAALKETERRSLDFLPVWKLGDDVARSVSPRQREAQDA--VALIRKTTEDLIEN-------CRKQLEEATGVA-GSFNEDDYISESDPS
LUT-14  156 TYTALKEVETRSMDLLPTWRLPEKFLRVVSPRQRDAQDA--VTVIRDVTQRLVDD-------CKRMVEEEEKVGGAEEWARDYLNESNPS
LUT-43  156 TYTALKEVETRSMDLLPTWRLPEQFLRAVSPRQKAAQDA--VTIIRDVTQKLVDD-------CKRMVEEEEKVGGAEEWARDYLNESNPS
LUT-20  156 TYTALKEVETRSMDLLPTWRLPEQFLRIVSPRQRNAQDA--VTVIRDVTQRLVDD-------CKRMVEEEEKVGGAEEWARDYLNESNPS
LUT-26  158 TYTALKEVETRSMDLLPTWRLPEQFLRIVSPRQKAAQDA--VTVIQEVTTKLVDD-------CKKMVEEEEAVGGAEAWARDYLNDANPS
LUT-38  158 TYTALKEVETRSMDLLPTWRLPEPFLRVVSPRQKAAQDA--VKIIQEVTTKLVDD-------CKRMVEEEEKVGGAEEWARDYLNDSNPS
LUT-2   153 VYTLLREAEHRSTAPIAYWNI--PGIQFVVPRQKRCQEA--LVLVNECLDGLIDK-------CKKLVEEED-----AVFGEEFLSERDPS
LUT-7   152 VYTVLREAEHRSTAPLAYWNL--PGATIVVPRQRRCQEA--LRIVNDTLDGLIDK-------CKKLVEEED-----MEFNEEFLSDQDPS
LUT-49  162 VYTVLREAEYRSTYPLPYWQL--PLMMWLVPRQRQCVEA--LRIINTTLDSLIAK-------SKRLFDEED-----QEFGEDYLSDKDPS
LUT-53  132 VYTVLREAEYRSTYPLPYWQL--PLMMWLVPRQRQCVEA--LRIINTTLDSLIAK-------SKRLFDEED-----QEFGEDYLSDKDPS
LUT-15  152 VYTVLREAEYRSVTFIPYWKV--PPLRWLVPRQRQCQEA--LQVVNDTLDDLINR-------CKAVVEEED-----EEFVEEYMNTDDPS
LUT-44  152 VYTVLREAEYRSVTFIPYWKV--PPLRWLVPRQRKCQEA--LEVVNDTLDELISR-------CKKVVEEED-----EEFVEEYMNTDDPS
LUT-16  152 VYTVLREAEYRSVTFIPYWKV--PPLRWLVPRQRQCQEA--LQVVNDTLDDLINR-------CKAVVEEED-----EEFVEEYMNTDDPS
LUT-21  152 VYTVLREAEYRSVTFIPYWKV--PPLRWLVPRQRQCQEA--LKVVNDTLDELIDR-------CKKIVEEED-----EEFVEEYMNTDDPS
LUT-27  152 VYTVLREAEYRSVTFIPYWKV--PPLRWLVPRQKACQEA--LVVVNDTLNMLIAR-------TKKLVEEED-----EEFVEEYLNKADPS
LUT-39  152 VYTVLREAEYRSVTFIPYWKV--PPLRWLVPRQKACQEA--LVVVNDTLNMLIER-------TKKIVEDSD-----EEFVEEYLSGDDPS
LUT-3   164 VYSVLRESTVRSTAPFPYWKL--PGISLLVPRLRESDAA--LAIVNDTLDRLIAR-------CKSMVGRCC-----GGGGGGGGGSSAPT

LUT-1   238 -VLRFLIAAREEVDSTQLRDDLLSMLVAGHETTGEGGRCPLYLPDD--EPHPSCSMQAEVDAVLGSRLS-------PTMADYGQLRYVMR
LUT-6   242 RVVPFLNQPSNPPPSRRCVSLFPSPLPAASLARGSALTWTLYLLVQ--NPDKMAKAVAEVESVMGSRTA-------PTLADYGQLRYVMR
LUT-48  232 -VLRFLIASREEVSSRQLRDDLLSMLVAGHETTGSVLTWTLYLLEQ--NPRAMAKARAEVDAVMGDRAA-------PSVEDFMALRYVMR
LUT-52  232 -VLRFLIASREEVSSRQLRDDLLSMLVAGHETTGSVLTWTLYLLEQ--NPRAMAKARAEVDAVMGDRAA-------PSVEDFMALRYVMR
LUT-31  232 -VLRFLIASREEVGSQQLRDDLLSMLVAGHETTGSVLTWTLDLLAR--NPEQMKKAQEEVDRVLAGKSK-------PNMEDYMALKYCMR
LUT-56  238 -VLRFLIASREEVTSSQLRDDLLSMLVAGHETTGSVLTWTLYLLTK--HPEKMNRLQEEIDSILGSKLDSEEGVSSLTLDDMLSLNYLKW
LUT-14  236 -VLRYLIAAREEVSSTQLRDDLLSLLVAGHETTASVLTWGTYELLKPENAEQLRLLRAELDEVLGTRPF-------PTFADLPKMPYLER
LUT-43  236 -VLRYLIAAREEVSSTQLRDDLLSLLVAGHETTASVLTWGTYELLKPENAEQLRLLRAELDEVLGTRPY-------PTFADLPNMPYLER
LUT-20  236 -VLRYLIAAREEVSSTQLRDDLLSLLVAGHETTASVLTWGTYELLKPENAEQLRLLRAELDEVLGTRPY-------PTFADLAKMPYLER
LUT-26  238 -VLRYLIAAREEVSSTQLRDDLLSLLVAGHETTASVLTWGTFELLKPENAEQLRLLRAELDEVLGDKPF-------PDYADMLKLPYLER
LUT-38  238 -VLRYLIAAREEVSSTQLRDDLLSLLVAGHETTASVLTWGTYELLKPENAEQLRLLRAELDEVLGDKPF-------PTYEDMTKMPYLER
LUT-2   226 -ILHFLLASGDEISSKQLRDDLMTMLIAGHETTAAVLTWTLYLLSQ--HPEAAAAIRKEVDELLGDRK--------PGVEDLRALKMTTR
LUT-7   225 -ILHFLLASGDEISSKQLRDDLMTMLIAGHETTAAVLTWTLYTLAS--HPEATEAIRREVDEVLGDRA--------PNVEDFKSLRFTTR
LUT-49  235 -ILHFLLASGDEITSKQLRDDLMTMLIAGHETTAAVLTWTLFCLTD--RPDVLRRMQQEIDEVVGDGK--------PSMDQLRSLRLTTR
```

```
LUT-53   205 -ILHFLLASGDEITSKQLRDDLMTMLIAGHETTAAVLTWTLFCLTD--RPDVLRRMQQEV------------------------------
LUT-15   225 -ILHFLIASGDDVTSKQLRDDLMTLLIAGHETTAAVLTWTTFLLAK--HPEVKAKVFEEVDRVVGDRN--------PTVADMRALVYTTR
LUT-44   225 -ILHFLIASGDDVTSKQLRDDLMTLLIAGHETTAAVLTWTTFLLAK--HPEVKAKVFEEVDRVVGDRN--------PTVADMRELVYTTR
LUT-16   225 -ILHFLIASGDDVTSKQLRDDLMTLLIAGHETTAAVLTWTTFLLAK--HPEVKAKVFEEVDRVVGDRN--------PTVADMRALVYTTR
LUT-21   225 -ILHFLIASGDDVTSKQLRDDLMTLLIAGHETTAAVLTWTTFLLAK--HPDIKQKVFEEVDRVVGDRN--------PTVADMRELVYTTR
LUT-27   225 -ILHFLIASGDDVTSKQLRDDLMTLLIAGHETTAAVLTWTTYLLAT--HPEIKARVQAEVDEVCGDRN--------PTIADMMDLKFTTR
LUT-39   225 -ILNFLIASGDDVTSKQLRDDLMTLLIAGHETTAAVLTWTTYLLAT--HPEQMRKVQEEVDRVVGDRR--------PTIQDMMELKYTTR
LUT-3    237 -VLHFLLGSGEALNSRQLRDDLMTLLIAGHETTAAALTWALHLLVA--HPEVMKRVRDEVDWVLGDRL--------PGSDDLPLLRYTTR

LUT-1    319 CVNESMRLYPHPPVLLRRALVEDELPGGF-KVPVGQDVMISVYNIHHSPAVWD-DPEAFIPERFG--PLDGPVPNEQNTDFRYIPFSGGP
LUT-6    323 CVNESMRLYPHPPVLLRRALVEDELPGGY-KVPVGQDVMISVYNIHHSEAVWD-NPEAFIPERFG--PLDGPVPSEQNTDFRYIPFSGGP
LUT-48   313 CVNESMRLYPHPPVLLRRAQVADTLPGGY-SVPVGQDVMISVYNIHRSPAVWD-DPNDFRPERF---PLDEPVPSEQTTDYRFIPFSGGP
LUT-52   313 CVNESMRLYPHPPVLLRRAQVADTLPGGY-SVPVGQDVMISVYNIHRSPAVWD-DPNDFRPERF---PLDEPVPSEQTTDYRFIPFSGGP
LUT-31   313 CVNESMRLYPHPPVLLRRAMVPDELPGGL-TVPQGQDVMISVYNIHRSPAVWD-RPDDFLPERF---PLDGPVPNEQNTDYKYIPFSGGP
LUT-56   326 CVAESMRLYPHPPVLIRRALEDDTLPSGY-KVPKGQDVMISIYNVHHSPSVWD-DPDSFQPERWY-GPRDN--PNEKNTNFHYIPFSGGA
LUT-14   319 CFHESMRLYPQPPVYTRRAVVEDVLPNGM-TIPKNQDLLVSIYNLHRSPTSWGPTSQEFEPMRFG--PLANGQPNELNTDYRYVPFSAGP
LUT-43   319 CFHESMRLYPQPPVYTRRAVVEDVLPNGM-TVPKNQDLLVSIYNLHRSPDNWGPTSQEFEPMRFG--PLANGQPNELNTDYRYVPFSAGP
LUT-20   319 CFHESMRLYPQPPVYTRRAVVEDVLPNGM-TVPKNQDLLVSIYNLHRSPANWGPTSQQFEPMRFG--PLANGQPNELNTDYRYVPFSAGP
LUT-26   321 CFHESMRLYPQPPVYTRRAVVEDVLPHGLGTIPAGQDLLVSIYNLHRSPANWGPASQAFEPMRFG--PLSAGQPNELNTGYRYTPFSAGP
LUT-38   321 CFHESMRLYPQPPVYTRRAVVEDVLPKGLGVVPAGQDLLVSIYNLHRSPENWGPNSQVFEPMRFG--PLALGQPNELNTGYRYTPFSAGP
LUT-2    306 VINEAMRLYPQPPVLIRRALQDDHF-DQF-TVPAGSDLFISVWNLHRSPKLWD-EPDKFKPERFGP--LDSPIPNEVTENFAYLPFGGGR
LUT-7    305 VINEAMRLYPQPPVLIRRALQEDKF-DQY-VVPAGSDLFISVWNLHRSPELWD-EPDKFKPERFGP--LDGPIPNEVTENFGYLPFGGGR
LUT-49   315 VVNEAMRLYPQPPVLIRRALEDDVL-GGF-HVSAGSDIFISVWNIHRDTGYWT-RPEDFDPDRFS---LEGPVPNETTENFNYLPFGGGR
LUT-53   262 --NEAMRLYPQPPVLIRRALEDDVL-GGF-HVSAGSDIFISVWNIHRDTGYWT-RPEDFDPDRFS---LEGPVPNETTENFNYLPFGGGR
LUT-15   305 VINESMRLYPQPPVLIRRALEPVTL-GGY-NIDAGTDFFISVWNLHRNPRIWD-EPDAFKPERFP---IDAPMPNEYTEEYAYLPFGGGQ
LUT-44   305 VINESMRLYPQPPVLIRRALEPVSL-GGY-NIDAGTDFFISVWNLHRNPRIWD-EPDAFKPERFP---VEAPMPNEYTEEYAYLPFGGGQ
LUT-16   305 VINESMRLYPQPPVLIRRALEPVTL-GGY-NIDAGTDFFISVWNLHRNPRIWD-EPDAFKPERFP---IDAPMPNEYTEEYAYLPFGGGQ
LUT-21   305 VINESMRLYPQPPVLIRRALEPVTL-GGY-NIDAGTDFFISVWNLHRNPRIWP-EPDAFKPERFP---IEGPMPNEYTEDYAYLPFGGGQ
LUT-27   305 VINESMRLYPQPPVLIRRALEPVTL-DGY-KIDAGTDFFISVWNLHRNPRLWE-NPDKFDPDRFP---IDQKMPNEITENFAYLPFGGGQ
LUT-39   305 VINESMRLYPQPPVLIRRALEPVTL-DGY-KIETGTDFFISVWNLHRNPRLWP-EPDKFIPERFP---LDQKMPNEVTENFAYLPFGGGQ
LUT-3    317 VVNEALRLYPQPPVLIRRAMQDDVLPGGH-VVAAGTDLFISVWNLHHSPQLWE-RPEAFDPDRFGP--LDSPPPTEFSTDFRFLPFGGGR

LUT-1    405 RKCVG---------DQFALMEAVVALTVLLRQYDFQMV----PNQQIGMTTGATIHTTNGLYMYVKERG
LUT-6    409 RKCVG---------DQFALMEAVVALAVLLRQFDFSLV----PNQKIGMTTGATIHTTDGLYMYVKERR
LUT-48   398 RKCVG---------DQFALMEAVVALAVLLKRFDFELV----PDQDIGMTTGATIHTQNGLFMTVRERA
LUT-52   398 RKCVG---------DQFALMEAVVALAVLLKRFDFELV----PDQDIGMTTGATIHTQNGLFMTVRERA
LUT-31   398 RKCVG---------DQFALMEAVVALAVMIREFDFAPQ----PGHDPGMTTGATIHTKNGLYMTVAKRR
LUT-56   411 RKCIG---------DQFALLEAYTALSVLVRKFDFQLV----KGQNIGMTTGATIHTTEGMRMTYQPRM
LUT-14   406 RRCPG---------DKFAVYEGIVIWATMFRRLDLELK----AGHDVVMTSGATIHTKSGLLATVKARA
LUT-43   406 RRCPG---------DKFAVYEGIVIWATMIRRLDLELK----PGHDVVMTSGATIHTKNGLLATV----
LUT-20   406 RRCPG---------DKFAVYEXIVIWATMLRRLDLELK----AGHDVIMTSGATIHTKSGLLATV----
LUT-26   409 RRCPG---------DKFAVLEGMAIWAVLFRRLDMELV----AGHDVVMTSGATIHTRDGMLVNATRRE
LUT-38   409 RRCPG---------DKFAVLEGMAIWAVMFRRLDLTLV----AGHDVVMTSGATIHTRDGMLVNARR--
LUT-2    391 RKCIG---------DQFALFEAVVALAMLMRRYEFNLDESKGT---VGMTTGATIHTTNGLNMFVRRRD
LUT-7    390 RKCIG---------DQFALFEALVALAMLVRRYDFVLDTSKPP---VGMTTGATIHTTGGLYMHVKKRD
LUT-49   399 RKCIGMHFLLRPISDQFALFESLSALAVLLRRFEFRMAPGAPP---VNMTTGATIHTSEGLLMQVVPRT
LUT-53   345 RKCIG---------DQFALFESLSALAVLLRRFEFRMAPGAPP---VNMTTGATIHTSEGLLMQVVPRT
LUT-15   389 RKCVG---------DQFAIFESIVSLAMLMRRFDFELDESKHPDGECGMTTGATIHTTNGLHVKLKRRD
LUT-44   389 RKCVG---------DQFAIFESIVSLAMLMRRFDVELDASKHPDGECGMTTGATIHTTNGLHVKLTRRQ
LUT-16   389 RKCVG---------DQFAIFESIVSLAMLMRRFDFELDESKHPDGECGMTTGATIHTTNGLHVKLKRR-
LUT-21   389 RKCVG---------DQFAIFESIVSLAMLMRRFDFELDESKHPDGECGMTTGATIHTTNGLHVRLKRRE
LUT-27   389 RKCVG---------DQFALFESIITLAMVCRRFDFELDAKFHPDGECGMTTGATIHTTGGLHVKLKRRD
LUT-39   389 RKCVG---------DQFALFESIITLAMVCRRFDIDLDPAFHPDGECGMTTGATIHTTGGLHVKLTRRS
LUT-3    403 RKCVG---------DMFAIAECVVALAVVLRRYDFAPDTSFGP---VGFKSGATINTSNGLHMLISRRD
```

```
LUT-17  1   -GPIFLALYPYFRRYGGVFKLAFGPK---VFMVLSDPVVVREVLKE-KP---FSFDKG-VLAEILEPIMGQGLIPAPYAVWKNRRRQLVP
LUT-45  1   -GPIFLALYPYFRRYGGVFKLAFGPK---VFMVLSDPVVVREVLKE-KP---FSFDKG-VLAEILEPIMGQGLIPAPYAIWKNRRRQLVP
LUT-22  1   -GPIFLALYPYFRRYGGVFKLAFGPK---VFMVLSDPVIVREVLKE-KP---FSFDKG-VLAEILEPIMGQGLIPAPYAVWKNRRRQLVP
LUT-28  1   -GPIFLALYPYFLKYGGVFKLAFGPK---VFMVLSDPVIVRRVLKE-KP---FAFSKG-VLAEILEPIMGQGLIPAPYAVWKNRRRQLVP
LUT-40  1   -GPIFLALYPYFLRYGGVFKLAFGPK---VFMVLSDPVVVREVLKE-KP---FAFSKG-VLAEILEPIMGQGLIPAPYAVWKNRRRQLVP
LUT-18  1   -APLFVPLYDYYREYGGVYNLGAGPK---WFVVVSDPVAVRTMFKD-QA---DSFSKG-ILTDIMEPIMGDGLIPANKEIWAKRRPVIGA
LUT-23  1   -APLFVPLYDYYREYGGVYNLGAGPK---WFVVVSDPVAVRTMFKD-KA---DDFSKG-ILTDIMEPIMGDGLIPANKETWAKRRPVIGA
LUT-46  1   -APLFVPLYDYYREYGGVYNLGAGPK---WFVVVSDPVAVRTMFKD-RA---DSFSKG-ILTDIMEPIMGDGLIPANKEIWAKRRPVIGA
LUT-41  1   -APLFVPLYDYYRQYGGVYNLGAGPK---WFVVVSDPVVVRHMFKD-NA---DAFSKG-ILTDIMEPIMGDGLIPAPKEIWAKRRPTVGA
LUT-8   1   DQPLFKALYQWFIESGGVYKLVFGPK---AFIVVSDPVVVRHLLKD-NA---FNYDKG-VLAEILEPIMGRGLIPADLDTWRVRRRAVVP
LUT-50  1   DEPLFKALYQWFLDSGGVYKLAFGPK---AFIVVSDPVVVRHLLKE-NA---FNYDKG-VLAEILEPIMGKGLIPADLETWKPRRRAIVP
LUT-54  1   DEPLFKALYQWFLDSGGVYKLAFGPK---AFIVVSDPVVVRHLLKE-NA---FNYDKG-VLAEILEPIMGKGLIPADLETWKPRRRAIVP
LUT-32  1   DEPLFKALYKWFIESGGVFKLEFGPK---AFIVISDPLVVRHLLKE-NY---TNYDKG-VLAEILEPIMGKGLIPADLETWKVRRRAIVP
LUT-36  1   -------------EAEHSYKVCLLPK---AFLIVSDPTVVRHILSE-NA---LKYDKG-ILADILEPIMGKGLIPADLPTWQPRRRAVVP
LUT-34  1   -GPLFLPLYKYFKDCGGLYKLCFGPK---VFMVASDPLVIRHIMKD-NV---FSYDKG-VLTEILEPFMGQGLIPAPFQVWKERRRALVP
LUT-35  1   ---MFMPLNKYFREYGGVYKLSFAPVPQATFYVLSDPHAIKHVLKE-SP---NDFDKG-LLSEILEPILGKGLIPADPETWRQRRPVIQP
LUT-12  1   -GPLFLLLNKYFEVYGPIFNLSFGPK---SFLVVSDPVMARHVLRETSP---DQYCKG-MLAEILDPIMGKGLIPADPATWKVRRRAIVP
LUT-59  1   -GPLFLLLNKYFLENGPIFNLSFGPK---SFLVISDPVMAKHILRTAPA---DQYCKG-MLADILEPIMGKGLIPADPATWKVRRRAVVP
LUT-10  1   -GPLFLLLAKYYQDYGPIFNLSFGPK---SFLVISDPVMARHILRDSSP---EQYCKG-MLAEILEPIMGDGLIPADPKIWKVRRRAVVP
LUT-61  1   -GPLFLLLTKYHNELGPVFKLAFGPR---SFIVVADPSIMRYILRDGAM---N-YDKG-ILAEILAPILGNGLIPADPDVWRRRRRVITP
LUT-13  1   -GTMFIGLQNYYRNYGSPYKLCFGPK---SFLVISDPVQAKHILKD-AN---TNYDKG-VLAEILEPIMGKGLIPADPETWSIRRRQIVP
LUT-58  1   -GTMFIGLQNYYKKYGSPYKLCFGPK---SFLVVSDPVQARHLLRD-AN---KNYDKG-VLAEILKPIMGKGLIPADPETWRVRRPQIVP
LUT-11  1   -GTMFIGLQRYQQQYGSPYKLCFGPK---SFLVISDPVQAKHVLRD-AN---TLYDKG-ILAEILKPIMGKGLIPADPETWSVRRRAIVP
LUT-57  1   -GTMYLALNAFQQEFGPIYKLCFGPK---SFIVVSDPVITKEILKS-PP---TAYDKG-VLAEILEDIMGKGLIPADPVIWKQRRRAIVP
LUT-60  1   -GTLYLGLHEYSQRFGPVYKLCFGPK---SFIVVSDHAVAKHVLRE-NN---GGYNKG-VLAEILEDIMGKGLIPADPVTWKARRRAIVP
LUT-37  1   AAAIFLALKTYFDRYGSVYKMCFGPK---SFMVSSDPVIARHVLRE-NC---KNYDKG-ALALVLEDIMGKGLIPADPVTWAKRRRAIAP

LUT-17  82  GFHKAWLDHMVGL--FGHCSNAL------VRNL--------DKAAASGE-----------------VVDMEERFCSVSLDIIGLAVFNYD
LUT-45  82  GFHKAWLDHMVGL--FGHCSGEL------VRNL--------DAAAAAGE-----------------TVDMEERFCSVSLDIIGLAVFNYD
LUT-22  82  GFHKAWLDHMVGL--FGHCSNEL------VRNL--------DKSAEDGE-----------------VVDMEERFCSVSLDIIGLAVFNYD
LUT-28  82  GFHKAWLDHMVGL--FGDCSAQL------VKNLGASHLTLTDASIAAGNGVA--------------RIDMEERFCSVSLDIIGLAVFNYD
LUT-40  82  GFHKAWLDHMVGL--FGDCSTQL------VKNL--------DAEIAKGNGSA--------------IVDMEERFCSVSLDIIGLAVFNYD
LUT-18  82  GFHGAWLKHMCNL--FGASAMRL------ADKLD--------TFVESEK-----------------TVELESELYAMALDVIGKAVFNYE
LUT-23  82  GFHGAWLKHMCNL--FGASAMRL------AEKLD--------VAAEKGT-----------------TVELEGELYAMALDVIGKAVFNYE
LUT-46  82  GFHNAWLKHMCDL--FGASAMRL------ADKLD--------AAAETKE-----------------TVELEGELYSMALDVIGKAVFNYE
LUT-41  82  GFHGAWLKHMTNL--FGASATNL------ADKLER-------EWCDKDV-----------------AVNLEDELYAMALDVIGKAVFNYE
LUT-8   83  AFHRQYYDAMVTM--FGRCADRS------SDKLQ--------ALVEKGQVG-----------LGGRVVDMESEFLSLGLDIIGLGVFNYD
LUT-50  83  AFHKAYLETMVAM--FGACTQET------IRSLD--------ALTAD----------------GEGQTDMEEVFLSLGLDIIGLGVFNYD
LUT-54  83  AFHKAYLETMVAM--FGACTQET------IRSLD--------ALTAD----------------GEGQTDMEEVFLSLGLDIIGLGVFNYD
LUT-32  83  GFHKAYLDACVAM--FGRCTQHT------VDKVE--------AALAAASPAPD-------GSQGAAVLDMETEFLNLGLDIIGLGVFNYE
LUT-36  70  GFHSSWLQSMMAVGLFSRCSDRM------VGALK--------ESMQRGMGPMSS--CWRTQERAGEEVDLESMYSSVALDIIGEAVFNFK
LUT-34  82  GFHQAWLNRMCRM--FSECTDRL------SAKLD--------AVADTD-----------------EIIDMEENWNSCSLDIIGKAVFNYD
LUT-35  83  GFHMRWLERMTMT--FNECASIM------IDKLE--------GEADAG-----------------NLVDMEGMFNSVSLDIIGKAVFNYE
LUT-12  83  SFHKRWLNRMITL--FAERAEIL------ADDLQPKSA-------------------------KGQVVDMEERFCSVTLDIIGKAVFNYD
LUT-59  83  AFHKRWLKRMITL--FNERAEIL------CDDLTRK---------------------------EGTVIDMEERFCSVTLDIIGKAVFDYD
LUT-10  83  GFHKKWLNNMVTL--FGDCGERL------VNDLDARAT-------------------------AKTPVDMEERFCSVTLDIIGKAVFNYD
LUT-61  82  AFHKQWLASTLSL--FDECTMEL------VDDLKARHATAEPAPLPATVDAWTAWKADDAQPRALGAVDMEERFCSVSLDIIGRAVFDYD
LUT-13  82  AFHKAWLEHIVGL--FGYCNQPL------IDTLNKRVDGD-------------------------GKVEMESLFCSVALDIIGLSVFNYE
LUT-58  82  AFHRKWLEYMVGQ--FGYCNKPL------IDSLNILADTT-------------------------GKVEMEEKFCSVALDIIGKSIFNYD
LUT-11  82  AFHKAWLNHMVGL--FGYCNEGL------IASLEEAAKKNDAP-----------------NGQQGGKIEMEEKFCSVALDIIGLSVFNYE
LUT-57  82  GFHKRWLDGMISV--FGRASSTL------IEDLDVAATTG-------------------------EPRDMEERFGSVALDIIGKAVFNYE
LUT-60  82  AFHKRWLARMLTM--FADET----------ELLNAELPLG-------------------------EPVDLEERFGSLALDIIGSAVFNYD
LUT-37  83  GFHKLYLERMVSE--FGQANANL------IPQLL-------------------------------------QAYGSLALDVIGKAVFNYE

LUT-17  139 FGSVTK---ESP----IISAVYNCLQEAAHRSTFYFPYWNL--PFATD-------IVPRQREFKKN--MSIINDTLNGLIKQ-------A
LUT-45  139 FGSVTK---ESP----IISAVYNCLQEAAHRSTFYFPYWNL--PFATD-------IVPRQREFKQN--MAVINDTLNGLIAQ-------A
LUT-22  139 FGSVTK---ESP----IISAVYNCLQEAAHRSTFYFPYWNI--PFATD-------IVPRQREFKQN--MKIINETLNGLIQK-------A
LUT-28  150 FGSTTR---ESP----IIKAVYTCLQEAAHRSTFYFPYWNI--PFMCD-------IVPRQREFKAN--MKLINDTLNGLITQ-------A
LUT-40  142 FGSTTR---ESP----IIKAVYTCLQEAAHRSTFYFPYWNL--PLADV-------LVPRQREFKNN--MNLINETLNGLIKK-------A
LUT-18  139 FGALKQ---ETP----IIKAVYRVLRESEHRSTFPLQYWQI--PGAME-------LVPRQKQFKED--MKMVNDELSVLINN-------A
LUT-23  139 FGALKQ---ETP----IIKAVYRVLRESEHRSTFPLQYWQI--PGAMD-------LVPRQKQFKED--MKMINEELSTLINS-------A
LUT-46  139 FGALRE---ETP----IIKAVYRVLRESEHRSTFPLQYWQI--PGAMD-------VVPRQKQFKED--MKMINEELSVLINN-------A
LUT-41  140 FGALRE---ETP----LIKAVYRVLRESEHRSTFPLQYWNI--PGAMD-------VVPRQKQFKED--IAAINAELSKLIAD-------A
LUT-8   146 FGSITS---ESP----VIKAVYGVLKEAEHRSTFYLPYWNL--PLADV-------LVPRQAKFRRD--LRVINDCLDDLIRK-------A
LUT-50  141 FNSITK---ESP----VIKAVYGVLKEAEHRSTFYIPYWNI--PITKY-------IVPRQRKFNAD--LAVINACLDDLIAQ-------A
LUT-54  141 FNSITK---ESP----VIKAVYGVLKEAEHRSTFYIPYWNI--PITKY-------IVPRQRKFNAD--LAVINACLDDLIAQ-------A
LUT-32  150 FGSITS---ESP----VIEAVYGVLKEAEHRSTFYIPYWNL--PLTKY-------LVPRQRQFNAD--LAVK------------------
LUT-36  144 FLSVQR---KSP----VIDAVYNLMQEAEHRSFFLLPYWKV--PVLGFRFLGLGPLVERQQRFEQD--IELINDCLDELIKE-------A
LUT-34  139 FGSVEK---LSP----VVEAALCALREAEHRSTFYFPYWKI--PGLGAEWP-IPALVPRQRKFQQD--MALLNGVLDKLILN-------V
LUT-35  140 FGSVTR---ESP----VIKAAYACLKEAEHRSTFLLPYWNV--PFLGQGK---FSVVPRQREFAAH--LEVLNDTLDTIIQK-------A
LUT-12  140 FGSVTD---ESP----IIKAVYRVLREAEHRSSSFIPYWNL--PYADQ-------WMGGQVEFRKD--MTMLDDILADLINK-------A
LUT-59  138 FGSVTK---ESP----IIKAVYRVLREAEHRSSSFIPYWNL--PYADQ-------WMGGQVEFRTD--MTMLDDILAKLINR-------A
LUT-10  140 FGSVTK---ESP----IVKAVYRVLREAEHRSSSFIPYWDL--PYADK-------WMGGQVEFRKD--MGMLDDILTKLINR-------A
LUT-61  164 FGSAKA---ESP----LVRAVYRCLIEAEKRTTAFIPFWLI--P-GAQ-------FLPSQVAFKND--FDLLNAKLDELVAQ-------A
LUT-13  139 FGSVTQ---ESP----VIKAVYSALVEAEHRSMTPAPYWNL--PLANQ-------LVPRLRKFNSD--LKLLNDVLDDLITR-------A
LUT-58  139 FGSTTD---ESP----VIKAVYSALVEAEHRSMTPAPYWDI--PLANQ-------LVPRLRKFNTD--LKLLNDVLDELIDK-------A
LUT-11  147 FGSVSE---ESP----VIKAVYSALVEAEHRSMTPAPYWDL--PFANE-------VVPRLRKFNSD--LKVLDDVLTDLIDR-------A
LUT-57  139 FESTQK---TSP----VVKAAIDTLREAEHRSMIPLPYWKL--PLADR-------LIPRQRAFASN--MALMNEKLDTAIEA-------A
LUT-60  135 FDSVRE---PSR----VVKAAIDTLREAEHRSMTPAPYWKI--PGAMQ-------VVPRQRAFTEN--MDLLNGELNKAIAA-------A
LUT-37  128 FGSVDE---ESP----VV---------------------------------------------------------LYKLIDE-------C

LUT-17  204 QQFEGTDD---LEELQNRDYSKVKDPS-LLRFLVDIRGADVTDVQLRDDLMTMLIAG-----------HETTAAVLTWGLFCLVQ----K
LUT-45  204 QQYEGTDD---LEELQNRDYSKVKDPS-LLRFLVDVRGADVTDAQLRDDLMTMLIAG-----------HETTAAVLTWGLFCLVQ----K
```

```
LUT-22  204 QKFEGTED---LEELQNRDYSKVKDPS-LLRFLVDIRGADVTDSQLRDDLMTMLIAG-----------HETTAAVLTWGLFCLMQ----N
LUT-28  215 QQFEGTED---LEELQNRDYSKVKDPS-LLRFLVDIRGADVTDLQLRDDLMTMLIAG-----------HETTAAVLTWCLFCLVR----D
LUT-40  207 QAFEGTED---LEELQNRDYSKVKDPS-LLRFLVDIRGADVTDSQLRDDLMTMLIAG-----------HETTAAVLTWCLYCLAQ----D
LUT-18  204 IASRNETG---LEEMERRDYSNVEDAS-LLRFLVDIRGDEATSTQLRDDLMTMLIAG-----------HETTAAVLTWTLYLLAQ----H
LUT-23  204 IESRNETG---LEEMERRDYSNVEDAS-LLRFLVDIRGDEATSTQLRDDLMTMLIAG-----------HETTAAVLTWTLYLLAQ----H
LUT-46  204 ISSRTETD---LEEMERRDYSKVEDAS-LLRFLVDIRGDEATSTQLRDDLMTMLIAG-----------HETTAAVLTWTLYLLAQ----H
LUT-41  205 LADRNETD---LAEMESRDYANVEDAS-LLRFLVDVRGETVSSTQLRDDLMTMLIAG-----------HETTAAVLTWTMYLLAT----H
LUT-8   211 QETRVEED---AEALQNRDYSKLRDPS-LLRFLVDMRGEDVTNKQLRDDLMTMLIAG-----------HETTAAVLTWALYCLMQ----S
LUT-50  206 KQTRQADD---VEALQARDYSKVRDPS-LLRFLVDMRDADLSDKQLRDDLMTMLIAG-----------HETTAAVLTWTLFALLT----H
LUT-54  206 KQTRQADD---VEALQARDYSKVRDPS-LLRFLVDMRDADLSDKQLRDDLMTMLIAG-----------HETTAAVLTWTLFALLT----H
LUT-32  203 ------------------------DAS-LLRFLVDMRDADLEAKQMRDDLMTMLIAGEWRRAAALRLCHETTAAVCTWTLFCVVQ----D
LUT-36  216 LLTRSEED---IETLQKRDYDALENPS-LLRFLVDMRGADATERQLRDDLMTMMIAG-----------HETTAALLTWTTFCLLT----N
LUT-34  210 VNEKQETD---LDALINKDYDNVNDPS-LLRFLVDLRGADATQKQLRDDLITLLIAG-----------HETTGSMLTWATWLLSQ----H
LUT-35  209 KSLKNEDD---LEALERRDYKSIQDPS-LLRFLVDLRGGDCNDKQLRDDLMTLLVAG-----------HETTGSLLTWTAFELAQ----N
LUT-12  205 VSTRREAS-----IEELEKRENEDDPS-LLRFLVGMRGEDLSSMVLRDDLMTMLIAG-----------HETTAAMLTWTLFELSR---GD
LUT-59  203 VSTRSELT-----VEELEDRDNAEDPS-LLRFLVDMRGEDVSSTVLRDDLMTMLIAG-----------HETTAAMLTWTLFELAQ---GE
LUT-10  205 IETRDEAS-----VEELEDRDVGDDPS-LLRFLADMRGEDLTSKVLRDDLMTMLIAG-----------HETTAAMLTWTVFGLVS---ND
LUT-61  228 FEEQIDDDDM-LEAGQEVTKASTERIS-LLRFLVTIRGEEASTGQLRDDLMTMLVAG-----------HETTAALLTWTLYELFHPSKRA
LUT-13  204 KQTRTVED---IEELENRNYNEVQDPS-LLRFLVDMRGADIDNKQLRDDLMTMLIAG-----------HETTAAVLTWALFELTK----N
LUT-58  204 KRTRTVGD---IEQLESRNYAEAKDPS-MLRFLVDMRGADIDNKQLRDDLMTMLIAG-----------HETTAAVLTWSLFELSK----H
LUT-11  212 KNSRQVED---IEELEKRDYANVKDPS-LLRFLVDMRGADIDNKQLRDDLMTMLIAG-----------HETTAAVLTWALFELTK----H
LUT-57  204 LSSRDEAD---LETIENRDYNAMENPS-LLRFLVDMRDEPTSSRQLRDDLMTMLIAG-----------HETTASALTWALFELMQ----Q
LUT-60  200 LADRVEEA---TEELERRDYATMENPS-LLRFLVDQRGEEATSTQLRDDLMTMLIAG-----------HETTASALTWCLFELAQ----N
LUT-37  147 LDSRNPEE---LDALKSKDYSKVKDPP-SP------------------------TPR-----------HETTAAVLTWLTYALSQ----H

LUT-17  275 PELLKRIQADIDEVF-----GDDD------RTPTYDDIQKLESVR-LCIAEALRLYPEPPILIRR----------CLEDVTLPKGAGD--
LUT-45  275 PELMKRIQADIDEVF-----GDDD------RAPTYEDIQRLESVR-LCVAEALRLYPEPPILIRR----------CLEDVTLPKGAGD--
LUT-22  275 PELMKRIQADIDEVM-----GDDD------RTPTYDDIQKLESVR-LCIAEALRLYPEPPILIRR----------CLEDVTLPKGAGD--
LUT-28  286 KPLMKKVVEEIDSVM-----GPVA-EE--ARAPNYEEIQKLELVR-LCLAEALRLYPEPPILIRR----------CLEDVPLPKGAGD--
LUT-40  278 RELMARVVAEIDDVM-----GPADGET--PTAPNYEQIQKMELVR-LCLAEALRLYPEPPILIRR----------CLEDVPLPKGAGD--
LUT-18  275 PEIADDAVAEINACV-----ENAD------GIPTPEEVRKLEKVR-MILAEGMRLYPAPPILIRR----------AIKDVTLPRGGNG--
LUT-23  275 PEIMEEAVKEIEMCV-----ENAD------GVPTPEEVRKLEKVR-MILAEGMRLYPAPPILIRR----------AIKDVTLPRGGNG--
LUT-46  275 PEIAEEAVEEINACV-----SDSN------GVPTPEEVRKLQKVR-MILAEGMRLYPAPPILIRR----------AIEDVTLPRGGNG--
LUT-41  276 PEEAELARAEVDAIV-----ADPS------GVPTVEEIRKLERTR-LCLAEGMRMYPAPPILIRR----------ALEDVTLPAGGMG--
LUT-8   282 PAALERVLREVDGVE-----RGGN--------PQGETVADLEACKGDPLGESLRMYPQPPILIRR----------ALGEDVLPGGLRG--
LUT-50  277 PSAYAKVLAEIDSVL-----GDKT--------PSIEDMRAMPYVR-CALAESLRMYPQPPILIRR----------ALSDDVLPAPLGG--
LUT-54  277 PSAYAKVLAEIDSVL-----GDKT--------PSIEDMRAMPYVR-CALAESLRMYPQPPILIRR----------ALSDDVLPAPLGG--
LUT-32  265 ERVEGKVLAEIDAAV-----GDRV--------PTWDDFANLPYTR-MTIAEAMRLYPQPPILIRRQAGAGGWVRVALGEDVLPAGLGG--
LUT-36  287 PEEMKKVHQEIEDVL-----GGRR--------ATYEDILKMEKTR-LALAEALRLYPQPPILIRR----------ALDDDVLPLAWGN--
LUT-34  281 PEAQAKMQKEIDDVL-----GGRS--------PTYEDMPRLEQVR-LVITETLRLFPEPPILIRR----------ALDADVLPKASN---
LUT-35  280 PAEMRKVQEEVDRVL-----GGRN--------PTMDDIKKLEYTR-LVLAEGLRLYPQPPILLRR----------ALKETKLPVAHSGSH
LUT-12  275 PGLLKEVQAEVRT-----VLKGKERP-------DYDDIVAMKKLR-YSLIEALRLYPEPPLLIRR----------ARTEDNLPA------
LUT-59  273 PGMFEEIQNEVRT-----VLKDKDRP-------DYDDVVAMKKLR-YALIEGLRLYPEPPVLIRR----------ARTEDILPQ------
LUT-10  275 SGLMKEIQAEVRT-----VMGDKLRP-------DYDDIAKMKKMR-YALIEALRLYPEPPVLIRR----------ARSEDNLPA------
LUT-61  305 AGHLERLRAEVDANFALRKSENRTAT-------AYADVVDCAFAR-LCLAEGLRLYPQPPLLIRR----------ALDSDELP-------
LUT-13  275 PEIMKELQDEIDEVV-----GDRMP--------NYEDIKKMKFLR-LVVAETLRMYPEPPLLIRR----------CRTPDELP-------
LUT-58  275 PEILATVVEEIDRVV-----GDRAP--------NLDDIKEMKQVR-LVIAETLRMYPEPPLLIRR----------CRTEDKLP-------
LUT-11  283 PEQMAKVRAEIDSVL-----GDRTP--------TYDDIKEMQYLR-LVVAETLRLYPEPPLLIRR----------CRTENKLP-------
LUT-57  275 PELLQKIRKEVDEVI-----GDRAP--------TYEDIVNMPLVR-VTIAESLRMYPEPPLLIRR----------ALEETVLP-------
LUT-60  271 RPLLEELRAELDAKLP----GGRPPR-------TLDEVRAVELTR-LTVAESLRMYPQPPLLIRR----------AVDDDAVPTVQLPDT
LUT-37  194 PEALRRVQDEIDTVVG-------------------DRYATVDDIKRMPEVQKADQWPEGGTGVEG-------------------------

LUT-17  340 ------------VEVTLIKGMDIFIS-------VWNLHRSPECW-ENPDEFDPFRFK-----------RPFKNPGVKD-WAGYNPDLL--
LUT-45  340 ------------REITLIKGMDIFIS-------VWNLHRSPECW-ENPDEFDPFRFK-----------RPFANPGVKD-WAGYDPDLL--
LUT-22  340 ------------AEVTLIKGMDIFIS-------VWNLHRSPECW-ENPEEFDPFRFK-----------RPFANPGVKD-WAGYNPELF--
LUT-28  354 ------------ADVTLIKGMDVFIS-------VWNLHRHPDCW-EEPLKFDPFRFK-----------KPYSNPGVKD-WAGYNPDLI--
LUT-40  347 ------------ANVTLIKGMDVFIS-------VWNLHRHPDCW-EEPLKFDPTRFK-----------RPFQNPGVKD-WAGYNPDLI--
LUT-18  340 ------------KEITLKAGTDCFIA-------VWNLHRSPDLW-EDPEKFDPSRFS-----------RRFENPAIEG-WGGLNPELM--
LUT-23  340 ------------KEITLKAGTDCFIA-------VWNLHRSPDLW-ENPEKFDPSRFS-----------RRFTNEAIEG-WGGLNPDLM--
LUT-46  340 ------------KEITLKAGTDCFIA-------VWNLHRSPDLW-ENPEKFDPSRFS-----------RRFENPAIEG-WGGLNPDLM--
LUT-41  341 ------------REITLKKGTDCFVA-------VWNLHRSPDLW-DRPDVFDPARFK-----------REFKNPKIEG-WNGLSPELV--
LUT-8   346 ----------DPAGYPIGTGADLFIS-------VWNLHRSPYLW-KDPDTFRPDRFF-----------ESYSNPDFEGKWAG--AYAVSG
LUT-50  340 ----------DSSGYPIGKGADIFISSSSGTIMFQSLHRSPHLW-KDPDTFRPERFS-----------ETNSNPAFNGAWAGYRPEAQ--
LUT-54  340 ----------DSSGYPIGKGADIFISSSSGTIMFQSLHRSPHLW-KDPDTFRPERFS-----------ETNSNPAFNGAWAGYRPEAQ--
LUT-32  338 ----------DPNGYPIGKGADLFIS-------LWNLHRSPHLW-KDPDTFRPERFTGQLG-------ERFVNAAFGGKWAGYTPGGE--
LUT-36  350 ----------EKQ-VKVFRGTDIFML-------VWNLHRSPVLWGDDADAFRPDRWL-----------SSRSNPDVPG-WEGYKPNMK--
LUT-34  343 -----LDGSVQGNAVKIIKGSDFFLS-------VWNLHRSPLLW-DKPDEFDPDRWRRPTPPELVEKYNAQRRAEGLPEWQGYVPDLK--
LUT-35  346 E--DQASSDMQPSGVSISPGANIFIS-------VWNLHRNPKLW-DNPDSFDPSRWLRPQP-----------ATNGHSSWAGYTPRKD--
LUT-12  335 ------GSSDLKSGVKVLRGTDMFIS-------TWNLHRSPDLW-ENPEVFDPTRWD-----------RPFNNA-GIPGWSGYNPDK--V
LUT-59  333 ------GSSVMKDGIKVLRGTDIFVS-------TWNLHRSPELW-ENPLTFDPTRWE-----------RSFKNP-NVKGWAGYDPDK--V
LUT-10  335 ------GGSGLSGGVKVLRGTDIFIS-------TWNLHRAPEYW-ENPEKYDPTRWE-----------RRFKNP-GVKGWNGYDPEKQSE
LUT-61  369 ------------------------------------------------------------------------------------------
LUT-13  333 --------QGAGREAKVIRGMDIFMA-------VYNIHRDERFW-PSPDTFDPLRFT-----------RSHSNP-DVPGWAGFDPKKWEG
LUT-58  333 --------AGGGREATVIRGMDIFLP-------LYNIHRDERFW-PNPDTFDPQRFT-----------RPYKNP-DIPDWAGFDPEKWSK
LUT-11  341 --------KGGGREATVIRGMDIFLS-------LYNLHHDERFW-PEPNEFKPERWE-----------SKYINP-EVPEWAGYDPAKWIN
LUT-57  333 -------KGGAEQETKLPRGADIFIS-------SYNLHRSPLFW-EEPDRFNPERFF-----------KPFKNPEAAPYWKGYDPKLWKG
LUT-60  339 DELDASGLRARAVDVKVPRACDMFIA-------IYSLHRNPRYW-KNPDSFDPKRWL-----------EKYANP-DEPTWAGYDPAKWKA
LUT-37  239 -------------GFALARANDLFIS-------TYNMGRSPQLW-EEPDVFDPQRWD-----------RPFDNPDVKG-WAGYDPAKRTG

LUT-17  396 ---------TGLYPNEVASDFAFIPFGAGARKCIG------------DQFAMLEATIAMAMTLRRYDFELQKDPK---------------
LUT-45  396 ---------TGLYPNEIASDYAFIPFGAGARKCIG------------DQFAMLESTVAMTMTLRDFDFELQKNPE---------------
LUT-22  396 ---------TGLYPNEVASDFAFIPFGAGARKCIG------------DQFAMLEATIAMAMVLRRYDFELTTDPK---------------
LUT-28  410 ---------SGMYPNEVTSDFAFVPFGAGARKCIGDQARSCLHWSPYDRFAMLEATSCLAMTLQRYDFELDKDAA---------------
```

```
LUT-40  403 ---------SGLYPNEVTSDFAFIPFGAGARKCIG------------DQFAMLEATSCLAMTLRRYDFEMTKDAS---------------
LUT-18  396 ---------TGLYPNEQCTDFSYVPFGGGQRRCAG------------DQFAMLEAVTALSVLLKKFKFELACEPG---------------
LUT-23  396 ---------TGLYPNEQSTDFSYVPFGGGQRRCAG------------DQFAMLEAVTSLSVLLKKFKFELDCPPN---------------
LUT-46  396 ---------TGLYPNEQSTDFSYVPFGGGQRRCAG------------DQFAMLEAVTAMSVLLKKFDFKLACSPS---------------
LUT-41  397 ---------TGLYPNEQSTDFAYVPFGGGQRRCAG------------DMFAMMEATVALSVLLKRFEFELGCDES---------------
LUT-8   406 G--------AALYPNEVGSDFAFIPFGGGARKCVG------------DQFAMFEATVALAVLLRRFSFALEGPPE---------------
LUT-50  407 G--------SSFYPNEVASDFAFIPFGGGARKCVG------------DQFALLEATVALTMLLRRFTFDFVEGPE---------------
LUT-54  407 G--------SSFYPNEVASDFAFIPFGGGARKCVG------------DQFALLEATVALTMLLRRFTFDFVEGPE---------------
LUT-32  402 G--------SSLYPNEVSSDFAFLPFGGGARKCIG------------DQFAVTEAALILVMLLRRFRFRLQD-PQ---------------
LUT-36  408 ----------NLYPNEVSSDFAFCPFGAGPRKCIG------------DQFAFLESVVILSRVLQEFDIQLATSPE---------------
LUT-34  418 ---------T-LYPNEVHADYAFLPFGAGPRKCLG------------DQFALMESVVMLTKIFQRYSFELVGNHDPK-V---------PN
LUT-35  412 ---------MGLYPNENDANYGYIPFGGGQRKCVG------------DQFAMQEAVVILSKLFQRFDIELAGSPE---------------
LUT-12  398 S---------GLYPSENAADFAFLPFGGGQRKCVG------------DQFAMMEATVTMALMIKKYDFDF-----------------AIP
LUT-59  396 S---------GLYPSENAADFAFMPFGGGSRKCVG------------DQFAMLEAAVTFSVIIKNFNFEF-----------------EGS
LUT-10  400 S---------SLYPNEITADYAFLPFGAGKRKCIG------------DQFAMLEASVTLAMIINKFDFTL-----------------VGS
LUT-61  369 -----------------------QPYADGRR--------------------------------------------------------TAP
LUT-13  395 ----------KLYPNEVASDFAFLPFGGGARKCVG------------DEFAILEATVTLAMVLRRFEFSFDESK-FE-GKDDILSSAQGL
LUT-58  395 ----------MLYPNEVASDFAYMPFGAGARKCVG------------DEFAIMEAVVTLSMVLRRFDFEFDLSK-ST-S-VDMYDPPQTA
LUT-11  404 T---------NLYPNEVASDFAYLPFGGGARKCVG------------DEFATLEATVTLAMLLRRFEFEFDSAKLAA-SKIDIMDHPEDL
LUT-57  397 ----------RLYPTEDSTDFAYIPFGGGSRKCVG------------DVFAMLEATVALAMILRRYDFDF--------------TAPTSE
LUT-60  409 GDGM-----GSLYPTETSADFAYLPFGGGARKCVG------------DQFAMMEATVALAGFLQRFDFDF--------------AGPTDT
LUT-37  297 TGKMKPEGGLWVANLEIASDHAMIPFGAGERKCVG------------DQFALLEAAVSVVMLLRRFEFDLEMPDGPVNPAKLDPDNPDKS

LUT-17  450 --DIGMEMGATIHTAGGLPMKIKRRT
LUT-45  450 --DVGMEMGATIHTAGGLPMKVTRRR
LUT-22  450 --DIGMTMGATIHTEKGLPCRVRRRQ
LUT-28  476 --EVGMEMGATIHTAGGLPMRVTRRK
LUT-40  457 --EVGMEMGATIHTAGGLPMKVTRR-
LUT-18  450 --EVEMITGATIHTKKGLPMKLKRR-
LUT-23  450 --DVEMITGATIHTKKGLPMKIKRRE
LUT-46  450 --DVEMITGATIHTKKGLPMKIKRRA
LUT-41  451 --EVEMITGATIHTKAGMPVKLRSRS
LUT-8   460 --KVGMATGATIHTANGLMVRVSRRT
LUT-50  461 --SVGMATGATIHTANGLQVRVQRRD
LUT-54  461 --SVGMATGATIHTANGLQVRVQRRD
LUT-32  455 --GVGMATGATIHTANGLKCTVERR-
LUT-36  461 --EVGMTTGATIHTEKGLKVSLRARK
LUT-34  477 ESDVGMMFGATIHTANGLNVRVKRRQ
LUT-35  466 --EVGMSTGATIHSKNGLMIRLKKRN
LUT-12  450 AEDVGMKTGATIHTMNGLMMRARQVN
LUT-59  448 PEDVGMQTGATIHTMNGLRMRQTRVK
LUT-10  452 PKDVGMKTGATIHTMNGLNLVVSRRS
LUT-61  381 PPPSGPPS--------------RSSP
LUT-13  462 NHPVGMRTGATIHTRNGLHLVVEKRG
LUT-58  461 DHPVGIRTGATIHTKNGLNMLVKKRQ
LUT-11  472 EHAVGMRTGATIHTRKGLHMVIRKRE
LUT-57  452 PSMVGTNTGATIHTRNGLWCKVSPRQ
LUT-60  468 PDKVGTNTGATIHTRNGLWMTVTERA
LUT-37  375 IGTVGMVSAATIHTATGLFCRVKERF
```
