# Supplementary material for: Evolutionary origins, molecular cloning and expression of carotenoid hydroxylases in eukaryotic photosynthetic algae
Source: BMC Genomics. 2013 Jul 8;14:457. doi: 10.1186/1471-2164-14-457 (PMC3728230; doi:10.1186/1471-2164-14-457)
Supplement: Additional file 5: Table S3 — List of primer sequences used for PCR amplification to clone three full-length cDNA of CYP97A/B/C homologs respectively in green alga Haematococcus pluvialis strain Flotow 1844. Note: F, forward; R, reverse; Position, the location of motifs where the primers were designed from protein multiple sequence alignments. [file 1471-2164-14-457-S5.pdf]

Evolutionary origins, molecular cloning and expression of carotenoid hydroxylases in eukaryotic photosynthetic algae

Hongli Cui[1, 2§], Xiaona Yu[3§], Yan Wang[2], Yulin Cui[2], Xueqin Li[4], Zhaopu Liu[3] and Song Qin[1*]

[1]Key Laboratory of Coastal Biology and Biological Resources Utilization, Yantai Institute of Coastal Zone Research, Chinese Academy of Sciences, Yantai 264003, People's Republic of China

[2]University of the Chinese Academy of Sciences, Beijing 100049, People's Republic of China

[3]College of Resources and Environmental Sciences, Key Laboratory of Marine Biology, Nanjing Agricultural University, Nanjing 210095, People's Republic of China

[4]Shenzhen Key Laboratory for Marine Bio-resource and Eco-environment, College of Life Sciences, Shenzhen University, Shenzhen 518060, People's Republic of China

[§]These authors contributed equally to this work.

[*]Corresponding author

E-mail addresses:

HLC: hlcui@yic.ac.cn

XNY: 2011103006@njau.edu.cn

YW: ywang@yic.ac.cn

YLC: yulincui@yic.ac.cn

XQL: 2110180316@email.szu.edu.cn

ZPL: sea@njau.edu.cn

SQ: sqin@yic.ac.cn

Additional file 5 - Table S3 List of primer sequences used for PCR amplification to clone three full-length cDNA of CYP97A/B/C homologues respectively in green alga *Haematococcus pluvial* strain flotow 1844.

Note: F, forward; R, reverse; Position, the location of motifs where the primers were designed from protein multiple sequence alignments. BCHQF and BCHQR primers were designed based on the sequence [GenBank: DQ257289.1].

Primers	5′-3′	Conserved regions (positions)
**Homologous cloning: HaeCYP97A/B/C**		
97AF	CCTGTCCGAGATCCTGGACTTYGTNATGG	SKGLLSEIIDFVMG (60-73)
97AR	GCGCTGGCCGCCNCCRAANGG	LPFGGGXRRCXG (415-426)
97BF	TGACCCTGGGCCTGGAYATHATHGG	GLDIIGLGVFN (5-15)
97BR	GCGCCGGTGGCCATNCCNAC	VGMATGATIHTANGL (368-382)
97CF	CCCTGTTCCAGCCCCTGTWYAARTGGAT	LXKWMXEXGXXYLLPP (40-57)
97CR	CGGGTGGGGGTACAGGCKCATNSWYTC	ESMRLYPXPPV (380-390)
**RACE: HaeCYP97A**		
GSP1-3’	GCGGGACGACCTGATGACC	567-585
GSP2-3’	CTGTGGGAGGAGCCAGAGG	898-916
GSP1-5’	CAACAAACTCGGCGTCCTCCT	465-485
GSP2-5’	TCCTCCTCCTCCACAAGCACC	450-470
**RACE: HaeCYP97B**		
GSP1-3’	CGAGGACGCCAGCAACAAGC	312-332
GSP2-3’	TCTGAGCCGCACAGCAACCC	733-752
GSP1-5’	TGCTTGTTGCTGGCGTCCTC	226-245
GSP2-5’	GCCGTCCAGGCAGTCGTTGAT	175-195
**RACE: HaeCYP97C**		
GSP1-3’	CGCTGAAGGAGACGGAGACAC	496-517
GSP2-3’	AAGGCGATGGTGGATGAAGAA	642-664
GSP1-5’	CAGTGGCTCGTGTCTCCGTCT	505-525
GSP2-5’	CCACCTGAGATGGCAAACCC	191-210
**Real–time PCR**		
BCHQF	GAGCCACCACAAACCCCGCACT	
BCHQR	TTGGGCAACCAGAAGCCGTAGG	
97AQF	CCACCTACCCCATTGCCTAC	

97AQR	GCGTCCTCCTCCTCCACA
97BQF	GGACGCCAGCAACAAGCAG
97BQR	TCCACCTCAGCCAGCACCT
97CQF	AAGGGGTTGGTGGCAGAGG
97CQR	GGACGATGGCACAAAGAAATG
ActinQF	AGCGGGAGATAGTGCGGGACA
ActinQR	ATGCCCACCGCCTCCATGC
