# Supplementary material for: Evolutionary origins, molecular cloning and expression of carotenoid hydroxylases in eukaryotic photosynthetic algae
Source: BMC Genomics. 2013 Jul 8;14:457. doi: 10.1186/1471-2164-14-457 (PMC3728230; doi:10.1186/1471-2164-14-457)
Supplement: Additional file 6: Figure S2 — Nucleotide and the predicted amino acid sequence of HaeCYP97A. [file 1471-2164-14-457-S6.pdf]

Evolutionary origins, molecular cloning and expression of carotenoid hydroxylases in eukaryotic photosynthetic algae

Hongli Cui[1, 2§], Xiaona Yu[3§], Yan Wang[2], Yulin Cui[2], Xueqin Li[4], Zhaopu Liu[3] and Song Qin[1*]

[1]Key Laboratory of Coastal Biology and Biological Resources Utilization, Yantai Institute of Coastal Zone Research, Chinese Academy of Sciences, Yantai 264003, People's Republic of China

[2]University of the Chinese Academy of Sciences, Beijing 100049, People's Republic of China

[3]College of Resources and Environmental Sciences, Key Laboratory of Marine Biology, Nanjing Agricultural University, Nanjing 210095, People's Republic of China

[4]Shenzhen Key Laboratory for Marine Bio-resource and Eco-environment, College of Life Sciences, Shenzhen University, Shenzhen 518060, People's Republic of China

[§]These authors contributed equally to this work.

[*]Corresponding author

E-mail addresses:

HLC: hlcui@yic.ac.cn

XNY: 2011103006@njau.edu.cn

YW: ywang@yic.ac.cn

YLC: yulincui@yic.ac.cn

XQL: 2110180316@email.szu.edu.cn

ZPL: sea@njau.edu.cn

SQ: sqin@yic.ac.cn

Additional file 6 - Figure S4 Nucleotide and the predicted amino acid sequence of HaeCYP97B.

```
   1    M  G  D  G  L  S  R  L  L  T  A  V  S  Q  G  Y  F  Q  P  D  V  G  G  A  S
   1 ACATGGGGGACGGCTTGTCTCGCCTTCTCACTGCCGTGAGCCAGGGCTACTTCCAGCCAGACGTAGGCGGCGCTT
  26    I  P  V  S  Q  G  E  L  S  D  L  A  G  D  E  P  L  F  K  A  L  Y  K  W  F
  76 CCATCCCCGTAAGCCAGGGGGAGCTGTCGGACCTGGCCGGAGATGAGCCGCTGTTCAAGGCCCTGTACAAATGGT
  51    L  D  C  G  G  V  Y  K  L  V  F  G  P  K  A  F  I  V  V  S  D  P  V  V  V
 151 TCCTGGATTGCGGAGGGGTGTACAAGCTGGTGTTTGGGCCCAAGGCCTTCATCGTGGTGTCTGACCCCGTGGTCG
  76    R  H  I  L  K  E  N  A  F  N  Y  D  K  G  V  L  A  E  I  L  E  P  I  M  G
 226 TGCGCCACATCCTGAAGGAGAATGCATTCAACTACGACAAAGGAGTGCTGGCGGAGATCTTGGAGCCCATCATGG
 101    K  G  L  I  P  A  D  L  E  T  W  K  V  R  R  R  A  V  V  P  A  F  H  K  Q
 301 GCAAGGGCCTCATCCCCGCAGACCTGGAGACCTGGAAGGTCCGGCGCCGGGCGGTGGTGCCTGCTTTCCACAAGC
 126    Y  Y  E  A  M  T  R  M  F  V  A  C  T  Q  R  T  A  D  K  L  Q  A  A  V  A
 376 AGTACTACGAGGCCATGACCCGCATGTTTGTGGCCTGCACCCAGCGCACTGCCGACAAGCTGCAGGCCGCGGTGG
 151    S  G  Q  G  S  A  V  L  D  M  E  A  E  F  L  N  L  G  L  D  I  I  G  L  G
 451 CCAGCGGGCAGGGCTCAGCGGTGCTGGACATGGAGGCAGAGTTCCTCAACCTGGGTCTGGACATCATTGGCCTGG
 176    V  F  N  Y  E  F  G  S  I  T  T  E  S  P  V  I  K  S  V  Y  G  V  L  K  E
 526 GTGTGTTCAACTACGAGTTTGGCTCAATCACTACCGAGTCCCCGGTCATCAAGTCAGTGTATGGGGTGCTTAAGG
 201    A  E  H  R  S  T  F  Y  I  P  Y  W  N  L  P  L  A  D  V  L  V  P  R  Q  A
 601 AGGCCGAGCACCGCTCCACCTTCTACATCCCCTACTGGAACCTGCCCCTGGCTGATGTACTGGTACCACGTCAGG
 226    Q  F  R  A  D  L  K  V  I  N  D  C  L  D  G  L  I  R  N  A  R  D  S  R  Q
 676 CCCAGTTCCGGGCCGACCTCAAAGTCATCAACGACTGCCTGGACGGCCTGATCCGCAACGCCAGGGACTCCCGGC
 251    E  D  D  A  E  A  L  Q  A  R  D  Y  S  Q  V  R  D  P  S  L  L  R  F  L  V
 751 AGGAGGACGACGCAGAGGCCCTGCAGGCCAGAGACTACAGCCAGGTGCGAGACCCCTCCCTCCTGCGCTTCTTGG
 276    G  M  R  G  E  D  A  S  N  K  Q  L  R  D  D  L  M  T  M  L  I  A  G  H  E
 826 TGGGCATGCGTGGCGAGGACGCCAGCAACAAGCAGCTGCGCGACGACCTGATGACCATGCTCATCGCGGGACATG
 301    T  T  A  A  V  L  T  W  A  L  Y  C  L  V  Q  H  P  Q  A  M  D  K  V  L  A
 901 AGACCACAGCCGCGGTGCTGACCTGGGCCCTGTATTGCCTGGTGCAGCACCCGCAGGCCATGGACAAGGTGCTGG
 326    E  V  D  A  V  L  G  G  G  R  L  P  G  I  D  D  L  K  A  L  A  F  T  R  A
 976 CTGAGGTGGATGCAGTGCTGGGGGGCGGCAGGCTCCCGGGCATTGACGACCTCAAGGCCCTTGCCTTCACCCGGG
 351    T  L  A  E  S  L  R  L  Y  P  Q  P  P  I  L  I  R  R  A  L  A  P  D  T  L
1051 CCACCCTGGCTGAGTCCCTGCGCCTGTACCCTCAGCCACCCATCCTGATCCGGCGCGCCCTGGCCCCGGACACCC
 376    P  P  G  L  K  G  D  P  A  G  Y  P  I  G  K  G  A  D  P  F  I  S  V  W  N
1126 TCCCCCCTGGCCTCAAGGGGGACCCTGCAGGGTACCCCATAGGCAAGGGCGCAGACCCCTTCATCAGCGTGTGGA
 401    L  H  R  S  P  H  L  W  K  D  P  D  T  F  R  P  E  R  F  S  E  P  H  S  N
1201 ACCTGCACCGCTCCCCACACTTGTGGAAGGACCCGGACACCTTTAGACCGGAGCGCTTCTCTGAGCCGCACAGCA
 426    P  A  F  G  S  A  W  A  G  Y  R  P  D  A  S  P  G  A  L  Y  P  N  E  V  T
1276 ACCCCGCCTTTGGCTCTGCCTGGGCAGGGTACCGGCCTGACGCCTCCCCTGGGGCGCTGTACCCCAACGAGGTCA
 451    S  D  F  A  F  I  P  F  G  G  G  A  R  K  C  I  G  D  Q  F  A  L  F  E  A
1351 CCTCCGACTTCGCCTTCATCCCCTTTGGCGGAGGGGCCCGAAAGTGCATCGGGGACCAGTTTGCGCTGTTTGAGG
 476    T  V  A  L  A  L  L  L  R  D  F  T  F  K  L  A  V  S  P  E  Q  V  G  M  A
1426 CTACGGTGGCGCTGGCCCTGCTGCTGCGCGACTTCACCTTCAAGCTGGCGGTCAGCCCAGAGCAGGTGGGCATGG
 501    T  G  A  T  I  H  T  A  N  G  L  P  M  K  I  T  I  R  R  A  V  N  S  G  P
1501 CTACTGGCGCAACCATCCACACCGCGAATGGCCTGCCGATGAAGATCACAATCCGGCGGGCAGTCAACTCCGGCC
 526    A  S  S  Q  P  A  M  A  G  V  T  K  A  S  *
1576 CTGCCTCCTCCCAGCCTGCTATGGCTGGGGTTACCAAGGCGTCCTGAGTGATGAGCTCTTGACAAGACGTTTGTT

1651 GATGGCAGATGGAGCAACGCCGTCATGCGTGATCAAGACAGCAGCCAAGCTTTTTATCAAGTACTGCCCAGCTGT

1726 TGTTTGCTCCGTGCCTGTGGCATTAACCAGAGAAACCCGCATGACAGCTCTGTGCAATGCCATTGCAATGTGCAT

1801 GCGACTCGGCCGGAACAATCTCTGATGCGATAATGACCAACACAACAAAAAAAAAAAAAAAAAAAAAAAAA
```
