# Supplementary material for: Evolutionary origins, molecular cloning and expression of carotenoid hydroxylases in eukaryotic photosynthetic algae
Source: BMC Genomics. 2013 Jul 8;14:457. doi: 10.1186/1471-2164-14-457 (PMC3728230; doi:10.1186/1471-2164-14-457)
Supplement: Additional file 7: Figure S3 — Nucleotide and the predicted amino acid sequence of HaeCYP97B. [file 1471-2164-14-457-S7.pdf]

Evolutionary origins, molecular cloning and expression of carotenoid hydroxylases in eukaryotic photosynthetic algae

Hongli Cui[1, 2§], Xiaona Yu[3§], Yan Wang[2], Yulin Cui[2], Xueqin Li[4], Zhaopu Liu[3] and Song Qin[1*]

[1]Key Laboratory of Coastal Biology and Biological Resources Utilization, Yantai Institute of Coastal Zone Research, Chinese Academy of Sciences, Yantai 264003, People's Republic of China

[2]University of the Chinese Academy of Sciences, Beijing 100049, People's Republic of China

[3]College of Resources and Environmental Sciences, Key Laboratory of Marine Biology, Nanjing Agricultural University, Nanjing 210095, People's Republic of China

[4]Shenzhen Key Laboratory for Marine Bio-resource and Eco-environment, College of Life Sciences, Shenzhen University, Shenzhen 518060, People's Republic of China

[§]These authors contributed equally to this work.

[*]Corresponding author

E-mail addresses:

HLC: hlcui@yic.ac.cn

XNY: 2011103006@njau.edu.cn

YW: ywang@yic.ac.cn

YLC: yulincui@yic.ac.cn

XQL: 2110180316@email.szu.edu.cn

ZPL: sea@njau.edu.cn

SQ: sqin@yic.ac.cn

Additional file 7 - Figure S5 Nucleotide and the predicted amino acid sequence of HaeCYP97C.

```
                                                M  Q  A  S  R  S  T  F  Q  H
   1 ACATGGGGGCCGTTTCGTTGTTGTTGTTTCGTTAATTGTGCTTCTGATGCAAGCTTCGCGGAGCACCTTCCAACA
  26  R  S  R  A  D  G  C  P  G  P  L  P  R  C  R  P  R  V  Q  R  S  R  G  R  N
  76 TCGTAGCCGAGCTGACGGCTGTCCCGGCCCATTGCCGCGATGCCGGCCGCGGGTACAGCGCAGTCGTGGTCGCAA
  51  C  V  V  L  N  A  I  D  D  E  G  T  S  G  K  S  I  D  A  A  G  A  G  A  T
 151 CTGCGTTGTGCTCAATGCTATAGACGACGAGGGGACGTCTGGGAAGTCAATTGATGCGGCTGGTGCAGGAGCAAC
  76  W  S  S  P  G  W  L  T  Q  L  N  M  L  W  S  G  K  G  N  I  P  V  A  D  A
 226 CTGGTCCAGTCCTGGATGGTTGACTCAGTTGAACATGCTGTGGTCTGGCAAAGGGAACATTCCTGTTGCTGATGC
 101  K  P  D  D  I  K  D  L  L  G  G  A  L  F  Q  A  L  Y  K  W  M  Q  E  S  G
 301 AAAGCCTGACGACATCAAGGACCTGCTGGGGGGGGCCCTCTTCCAGGCGCTGTACAAGTGGATGCAGGAGTCTGG
 126  P  V  Y  L  L  P  T  G  P  V  S  S  F  L  V  I  S  D  P  A  A  A  K  H  V
 376 GCCAGTGTATCTGCTGCCCACTGGGCCAGTGTCGTCCTTCCTGGTCATATCTGATCCTGCTGCAGCCAAGCACGT
 151  L  R  G  T  D  N  P  Q  R  P  L  Y  G  K  G  L  V  A  E  V  A  Q  F  L  F
 451 GCTGCGGGGGACTGACAACCCACAGCGGCCACTGTATGGGAAGGGGTTGGTGGCAGAGGTGGCTCAGTTCCTGTT
 176  G  D  G  F  A  I  S  G  G  D  A  W  R  V  R  R  K  A  V  A  P  S  L  H  R
 526 TGGCGACGGGTTTGCCATCTCAGGTGGAGACGCATGGAGAGTTAGGCGCAAGGCAGTGGCGCCCTCATTGCACAG
 201  A  Y  L  E  T  M  I  S  R  V  F  A  P  S  A  V  F  L  A  D  K  L  H  A  D
 601 AGCCTACTTGGAGACAATGATAAGCCGTGTATTTGCACCCAGTGCTGTGTTCCTGGCAGACAAGCTACACGCCGA
 226  P  A  A  G  N  S  T  Q  P  Q  P  V  N  M  E  A  A  F  S  Q  L  T  L  D  V
 676 CCCAGCTGCCGGTAACAGCACCCAGCCCCAGCCCGTGAACATGGAGGCGGCATTCAGCCAGCTGACGCTGGATGT
 251  I  G  K  A  V  F  N  Y  D  F  N  A  L  T  T  D  S  P  L  I  Q  A  V  Y  T
 751 CATCGGCAAGGCCGTGTTCAACTATGACTTCAACGCGCTCACCACAGACAGCCCACTGATCCAGGCGGTGTACAC
 276  A  L  K  E  T  E  T  R  A  T  D  L  L  P  Y  W  K  V  P  F  L  C  A  I  V
 826 GGCGCTGAAGGAGACGGAGACACGAGCCACTGACCTGCTGCCCTATTGGAAGGTTCCATTTCTTTGTGCCATCGT
 301  P  R  Q  R  K  A  A  A  A  V  Q  L  I  R  D  T  T  T  A  L  I  K  Q  C  K
 901 CCCCCGTCAGCGCAAGGCTGCCGCAGCAGTGCAGCTGATCCGGGACACCACCACCGCCCTCATCAAGCAGTGCAA
 326  A  M  V  D  E  E  E  I  A  A  A  S  A  A  S  A  E  G  K  E  Y  I  N  A  A
 976 GGCGATGGTGGATGAAGAAGAGATCGCTGCAGCCAGCGCCGCCAGTGCGGAGGGCAAGGAGTACATAAATGCAGC
 351  D  P  S  V  L  R  F  L  I  A  A  R  E  E  V  D  S  T  Q  L  R  D  D  L  L
1051 GGACCCCAGCGTGCTGCGCTTTCTCATAGCGGCCCGCGAGGAGGTTGACAGCACGCAGCTCCGCGATGACCTGCT
 376  S  M  L  V  A  G  H  E  T  T  G  S  A  L  T  W  T  L  Y  L  L  A  Q  N  P
1126 ATCCATGTTGGTCGCGGGCCACGAGACCACTGGCAGCGCGCTCACTTGGACATTGTACCTGCTGGCGCAGAACCC
 401  D  K  M  A  I  A  Q  A  E  V  D  A  V  M  G  A  R  R  S  P  T  I  A  D  Y
1201 TGACAAGATGGCCATTGCACAGGCCGAGGTGGACGCTGTGATGGGCGCTCGCCGTTCGCCGACCATTGCGGACTA
 426  M  A  L  R  Y  V  M  R  C  V  C  E  S  M  R  L  Y  P  H  P  P  V  L  L  R
1276 TATGGCCCTGCGCTATGTCATGCGGTGCGTGTGCGAAAGCATGCGCTTGTACCCGCACCCGCCGGTGCTGCTGCG
 451  R  A  F  A  A  D  T  L  P  G  G  Y  K  V  V  K  G  Q  D  V  M  I  S  V  Y
1351 CCGGGCTTTCGCGGCTGACACGCTACCCGGTGGCTATAAGGTGGTGAAGGGCCAGGACGTGATGATCAGCGTGTA
 476  N  I  H  R  S  K  A  V  W  D  S  P  E  A  F  L  P  E  R  F  G  P  L  D  G
1426 CAACATCCATCGCTCCAAAGCGGTGTGGGACAGCCCTGAGGCGTTTTTGCCAGAGCGGTTTGGACCACTGGACGG
 501  P  V  P  S  E  Q  N  T  D  F  R  Y  I  P  F  S  G  G  P  R  K  C  V  G  D
1501 CCCCGTGCCCAGCGAGCAGAACACGGACTTCCGCTACATCCCCTTCAGCGGGGGGCCGCGGAAGTGCGTGGGCGA
 526  Q  F  A  L  M  E  A  V  V  S  L  A  V  L  L  R  E  F  D  L  S  L  V  P  N
1576 TCAGTTCGCGCTGATGGAGGCGGTAGTGTCCCTCGCAGTGCTGCTCCGTGAGTTCGACTTGAGCTTGGTGCCGAA
 551  Q  T  I  G  M  T  T  G  A  T  I  H  T  T  N  G  L  Y  M  Y  A  R  A  R  I
1651 CCAGACCATTGGCATGACCACCGGCGCCACGATCCACACTACCAATGGCCTATACATGTACGCGAGAGCCCGAAT
 576  A  A  A  K  S  V  A  L  V  A  T  *
1726 TGCAGCAGCCAAGTCCGTGGCACTGGTTGCGACATGAGCTTGTGCGAGGAACAATACATTTTCAGCCCTGATTAG

1801 AGAACATTCCCAAGCATAAGCGGCGTTGTCGGTGAAGTGAGATTGGGAGCAGAGGTCTTGAAGGAAGACAGGGGC

1876 GCAGTTGCTCTTTATGACGATCGTCATCGTTATAGCATGCCATGCCGGCAATGGACATGTTGTCCGATCGTAAAC

1951 GTGGCTTGTGGCCTAAAAAAAAAAAAAAAAAAAAAAAAAAAAAAAA
```
