# Supplementary material for: Evolutionary origins, molecular cloning and expression of carotenoid hydroxylases in eukaryotic photosynthetic algae
Source: BMC Genomics. 2013 Jul 8;14:457. doi: 10.1186/1471-2164-14-457 (PMC3728230; doi:10.1186/1471-2164-14-457)
Supplement: Additional file 8: Figure S4 — Nucleotide and the predicted amino acid sequence of HaeCYP97C. [file 1471-2164-14-457-S8.pdf]

Evolutionary origins, molecular cloning and expression of carotenoid hydroxylases in eukaryotic photosynthetic algae

Hongli Cui[1, 2§], Xiaona Yu[3§], Yan Wang[2], Yulin Cui[2], Xueqin Li[4], Zhaopu Liu[3] and Song Qin[1*]

[1]Key Laboratory of Coastal Biology and Biological Resources Utilization, Yantai Institute of Coastal Zone Research, Chinese Academy of Sciences, Yantai 264003, People's Republic of China

[2]University of the Chinese Academy of Sciences, Beijing 100049, People's Republic of China

[3]College of Resources and Environmental Sciences, Key Laboratory of Marine Biology, Nanjing Agricultural University, Nanjing 210095, People's Republic of China

[4]Shenzhen Key Laboratory for Marine Bio-resource and Eco-environment, College of Life Sciences, Shenzhen University, Shenzhen 518060, People's Republic of China

[§]These authors contributed equally to this work.

[*]Corresponding author

E-mail addresses:

HLC: hlcui@yic.ac.cn

XNY: 2011103006@njau.edu.cn

YW: ywang@yic.ac.cn

YLC: yulincui@yic.ac.cn

XQL: 2110180316@email.szu.edu.cn

ZPL: sea@njau.edu.cn

SQ: sqin@yic.ac.cn

Additional file 8 - Figure S6 Changes in total chlorophylls and carotenoids concentrations of *H. pluvialis* under different high light stresses (white or blue).

Cells grown in autotrophic medium were harvested and transferred to fresh medium with different high light intensity. Cells were harvested at different periods of induction and changes in total chlorophylls (a), total chlorophylls (b), lutein (c), α–carotene (d), β–carotene (e), and astaxanthin (f). Values are mean ± SD of three independent determinations.

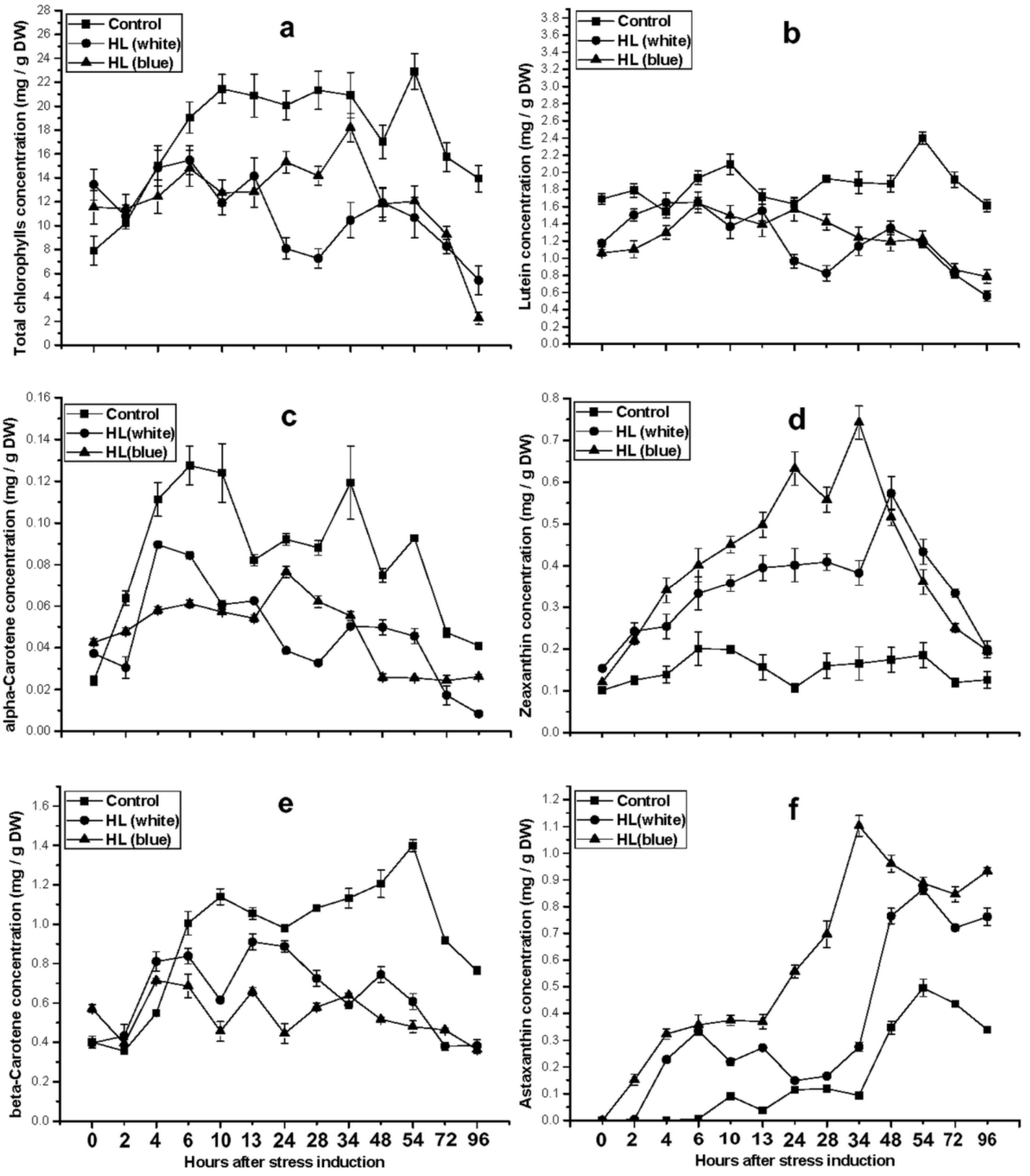
a
Control
HL (white)
HL (blue)
Total chlorophylls concentration (mg / g DW)
b
Lutein concentration (mg / g DW)
c
HL(white)
HL(blue)
alpha-Carotene concentration (mg / g DW)
d
Zeaxanthin concentration (mg / g DW)
e
beta-Carotene concentration (mg / g DW)
f
Astaxanthin concentration (mg / g DW)
0 2 4 6 10 13 24 28 34 48 54 72 96
Hours after stress induction
