# Supplementary material for: Evolutionary origins, molecular cloning and expression of carotenoid hydroxylases in eukaryotic photosynthetic algae
Source: BMC Genomics. 2013 Jul 8;14:457. doi: 10.1186/1471-2164-14-457 (PMC3728230; doi:10.1186/1471-2164-14-457)
Supplement: Additional file 10: Figure S6 — The transcriptional level of actin gene under high light stress of different wavelength (blue and white). [file 1471-2164-14-457-S10.pdf]

Evolutionary origins, molecular cloning and expression of carotenoid hydroxylases in eukaryotic photosynthetic algae

Hongli Cui[1, 2§], Xiaona Yu[3§], Yan Wang[2], Yulin Cui[2], Xueqin Li[4], Zhaopu Liu[3] and Song Qin[1*]

[1]Key Laboratory of Coastal Biology and Biological Resources Utilization, Yantai Institute of Coastal Zone Research, Chinese Academy of Sciences, Yantai 264003, People's Republic of China

[2]University of the Chinese Academy of Sciences, Beijing 100049, People's Republic of China

[3]College of Resources and Environmental Sciences, Key Laboratory of Marine Biology, Nanjing Agricultural University, Nanjing 210095, People's Republic of China

[4]Shenzhen Key Laboratory for Marine Bio-resource and Eco-environment, College of Life Sciences, Shenzhen University, Shenzhen 518060, People's Republic of China

[§]These authors contributed equally to this work.

[*]Corresponding author

E-mail addresses:

HLC: hlcui@yic.ac.cn

XNY: 2011103006@njau.edu.cn

YW: ywang@yic.ac.cn

YLC: yulincui@yic.ac.cn

XQL: 2110180316@email.szu.edu.cn

ZPL: sea@njau.edu.cn

SQ: sqin@yic.ac.cn

Additional file 10 – Table S3 The sequences information of BCHs from higher plants and CrtRs from cyanobacteria was downloaded from NCBI database or Cyanobase respectively.

Enzymes	Species	GenBank/cyanobase
BCH1	*Arabidopsis thaliana*	sp|Q9SZZ8.1
BCH2	*Arabidopsis thaliana*	sp|Q9LTG0.1]
BCH1	*Solanum lycopersicum*	NP_001234348.1
BCH2	*Solanum lycopersicum*	gb|ADF28628.1
BCH1	*Glycine max*	NP_001237423.1
BCH2	*Glycine max*	NP_001239934.1
BCH1	*Zea mays*	ACX49356.1
BCH1	*Brassica napus*	ABM54182.1
BCH2	*Solanum tuberosum*	gb|ADF28628.1
CrtZ	*Novosphingobium* sp. PP1Y	YP_004533851.1
CrtZ	*Paracoccus* sp. N81106	dbj|BAE47466.1
CrtZ	*Pedobacter* sp. BAL39	gb|EDM36688.1
beta-carotene hydroxylase	*Prochlorococcus marinus* str. NATL1A	NATL1_03151
beta-carotene hydroxylase	*Prochlorococcus marinus* str. NATL2A	PMN2A_1603
beta-carotene ketolase	*Synechococcus* sp. JA–2–3B'a (2-13)	CYB_0102
fatty acid desaturase	*Synechococcus* sp. JA–3–3Ab	CYA_1931
beta-carotene hydroxylase	*Arthrospira platensis* NIES-39	NIES39_R00430
fatty acid desaturase	*Trichodesmium erythraeum* IMS101	Tery_2925
beta-carotene hydroxylase	*Prochlorococcus marinus* SS120	Pro0266
beta-carotene hydroxylase	*Synechococcus* sp. RCC307	SynRCC307_2209
beta-carotene hydroxylase	*Microcystis aeruginosa* NIES–843	MAE07370
beta-carotene hydroxylase	*Synechococcus elongatus* PCC 6301	syc1667_c
beta-carotene hydroxylase	*Synechocystis* sp. PCC 6803	sll1468
beta-carotene oxygenase CrtR	*Synechococcus* sp. PCC 7002	SYNPCC7002_A0915
hypothetical protein	*Anabaena* sp. PCC 7120	alr4009
fatty acid desaturase	*Cyanothece* sp. PCC 7424	PCC7424_2363
fatty acid desaturase	*Cyanothece* sp. PCC 7425	Cyan7425_1008
beta-carotene hydroxylase	*Synechococcus* sp. WH 7803	SynWH7803_0337
beta-carotene hydroxylase	*Synechococcus elongatus* PCC 7942	Synpcc7942_2439
beta-carotene hydroxylase	*Synechococcus* sp. WH8102	SYNW0291
fatty acid desaturase	*Cyanothece* sp. PCC 8801	PCC8801_1623
beta-carotene hydroxylase	*Prochlorococcus marinus* str. MIT 9211	P9211_02591
beta-carotene hydroxylase	*Prochlorococcus marinus* str. MIT 9215	P9215_02581

beta-carotene hydroxylase	*Prochlorococcus marinus* str. MIT 9301	P9301_02581
beta carotene hydroxylase	*Prochlorococcus marinus* str. MIT 9303	P9303_24321
beta-carotene hydroxylase	*Synechococcus* sp. CC9311	sync_0336
beta-carotene hydroxylase	*Prochlorococcus marinus* MIT9312	PMT9312_0238
beta carotene hydroxylase	*Prochlorococcus marinus* MIT9313	PMT1816
Beta-carotene hydroxylase	*Prochlorococcus marinus* str. MIT 9515	P9515_02681
Beta-carotene hydroxylase	*Prochlorococcus marinus* str. AS9601	A9601_02571
beta-carotene hydroxylase	*Synechococcus* sp. CC9605	Syncc9605_0286
beta-carotene hydroxylase	*Synechococcus* sp. CC9902	Syncc9902_2058
beta-carotene hydroxylase	*Acaryochloris marina* MBIC11017	AM1_3637
fatty acid desaturase	*Nostoc punctiforme* ATCC 29133	Npun_R4276
fatty acid desaturase	*Anabaena variabilis* ATCC 29413	Ava_1693
beta-carotene hydroxylase	*Cyanothece* sp. ATCC 51142	cce_4622
beta-carotene hydroxylase	*Thermosynechococcus elongatus* BP–1	tlr1900
